# Modulation of Apoptosis Controls Inhibitory Interneuron Number in the Cortex

**DOI:** 10.1016/j.celrep.2018.01.064

**Published:** 2018-02-13

**Authors:** Myrto Denaxa, Guilherme Neves, Adam Rabinowitz, Sarah Kemlo, Petros Liodis, Juan Burrone, Vassilis Pachnis

**Affiliations:** 1 Nervous System Development and Homeostasis Laboratory, Francis Crick Institute, 1 Midland Road, London NW1 1AT, UK; 2 Centre for Developmental Neurobiology, Institute of Psychiatry, Psychology and Neuroscience, King’s College London, London SE1 1UL, UK; 3 MRC Centre for Neurodevelopmental Disorders, King’s College London, London SE1 1UL, UK; 4 Bioinformatics and Biostatistics Laboratory, Francis Crick Institute, 1 Midland Road, London NW1 1AT, UK; 5 Molecular Neurobiology, National Institute for Medical Research, the Ridgeway, Mill Hill, London NW7 1AA, UK

**Keywords:** cortical interneurons, Lhx6, interneuron cell death, interneuron transplantations, DREADS, homeostatic plasticity, interneuron development, activity-dependent plasticity

## Abstract

Cortical networks are composed of excitatory projection neurons and inhibitory interneurons. Finding the right balance between the two is important for controlling overall cortical excitation and network dynamics. However, it is unclear how the correct number of cortical interneurons (CIs) is established in the mammalian forebrain. CIs are generated in excess from basal forebrain progenitors, and their final numbers are adjusted via an intrinsically determined program of apoptosis that takes place during an early postnatal window. Here, we provide evidence that the extent of CI apoptosis during this critical period is plastic and cell-type specific and can be reduced in a cell-autonomous manner by acute increases in neuronal activity. We propose that the physiological state of the emerging neural network controls the activity levels of local CIs to modulate their numbers in a homeostatic manner.

## Highlights

• *Lhx6* is required for survival of CIs generated in the MGE
• MGE-derived CI loss is compensated for by a decrease in CGE-derived interneuron apoptosis
• Increases in cortical network activity are correlated with improved CI survival
• Transient, cell-autonomous depolarization improves the survival of grafted CIs

## Graphical Abstract

**Figure:**
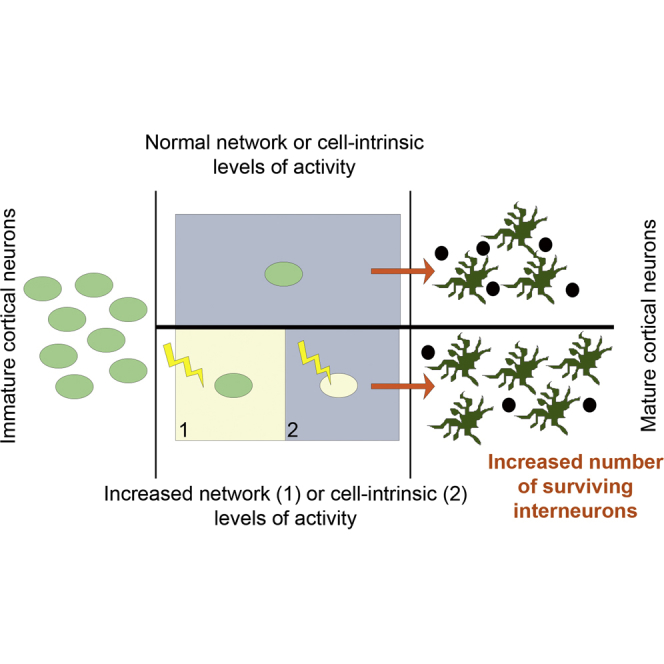

## Teaser

Denaxa et al. address how the number of interneurons in the cortex is regulated. They show that apoptosis of developing interneurons can be modulated by activity in the forebrain of young mice so that increases in activity can rescue interneurons from apoptosis. This feedback loop provides a mechanism for fine-tuning the number and repertoire of interneurons in the brain.

## Introduction

The balance between excitation and inhibition (E-I balance) is essential for the generation of optimal neural circuit activity and brain function. Cortical interneurons (CIs) represent the main source of γ-amino butyric acid (GABA)-mediated inhibition for excitatory projection neurons (PNs) in the pallium, and changes in the number or activity of CIs have been associated with neurodevelopmental and neuropsychiatric disorders such as epilepsy, schizophrenia, and autism spectrum disorders (Marín, 2012, Rubenstein and Merzenich, 2003). In contrast to cortical PNs, which are generated in the germinal zones of the dorsal telencephalon, CIs originate from progenitors in the subpallium (medial ganglionic eminence [MGE], caudal ganglionic eminence [CGE], and preoptic area [POA]) and, following stereotypic migration routes, reach the dorsal telencephalon, where they integrate into local circuits (Bartolini et al., 2013, Marín and Rubenstein, 2001, Wonders and Anderson, 2006). The disparate origin of PNs and CIs raises questions regarding the mechanisms that co-ordinate the size of these functionally interdependent neuronal populations of the cortex. A recent report has shown that CIs are generated in excess from basal forebrain progenitors and that BAX-dependent developmental cell death occurring over a critical postnatal period adjusts the final number of inhibitory neurons (Southwell et al., 2012). However, it is unclear whether postnatal apoptosis of CIs is controlled by an invariable cell-intrinsic program or can be modulated by the cellular composition and physiological state of the postnatal brain. We sought to address this question using genetic fate mapping to assess the survival of different CI populations in mutant mice characterized by reproducible changes in the cortical microenvironment.

Systematic gene expression analysis, genetic cell lineage tracing, and phenotypic characterization of mouse mutants have demonstrated that CI subtypes are specified by region-specific transcriptional programs operating within progenitor domains of the subpallium (Fishell and Rudy, 2011). *Lhx6* encodes a LIM homeodomain transcription factor that is specifically expressed by MGE-derived precursors and their derivative CIs expressing somatostatin (Sst) and parvalbumin (Pv). Consistent with its expression pattern, *Lhx6* mutations are characterized by a severe reduction in the number of Sst^+^ and Pv^+^ CIs but a normal complement of GABA-producing cells (Liodis et al., 2007, Zhao et al., 2008). These cellular phenotypes are associated with reduced inhibitory synaptic input on PNs, brain hyperactivity, and epilepsy-like phenotypes in postnatal animals (Neves et al., 2013). Here, we have combined phenotypic analysis of null and cell lineage-specific mutants, genetic lineage tracing, cell transplantation, and chemogenetic activation to query the specific responses of CI sub-lineages following the deletion of *Lhx6* activity. We find that *Lhx6* is required to maintain the normal complement of MGE-derived CIs and that reduction of this subpopulation in *Lhx6* mutants results in a surprising increase in the number of *Lhx6*-independent CGE-derived CIs and re-balancing of CI networks. The compensatory increase of CGE-derived CIs is due to a reduction in apoptosis that can be modulated cell-autonomously by neuronal excitability during a critical postnatal period. Our results provide fundamental insight into the mechanisms that match the size of CI populations to the physiological requirements of cortical circuits and pave the way for better understanding the effect of neuronal activity on cell transplantation-based therapies.

## Results

### Loss of MGE CIs Results in a Compensatory Increase in the Number of CGE CIs

**Figure 1:**
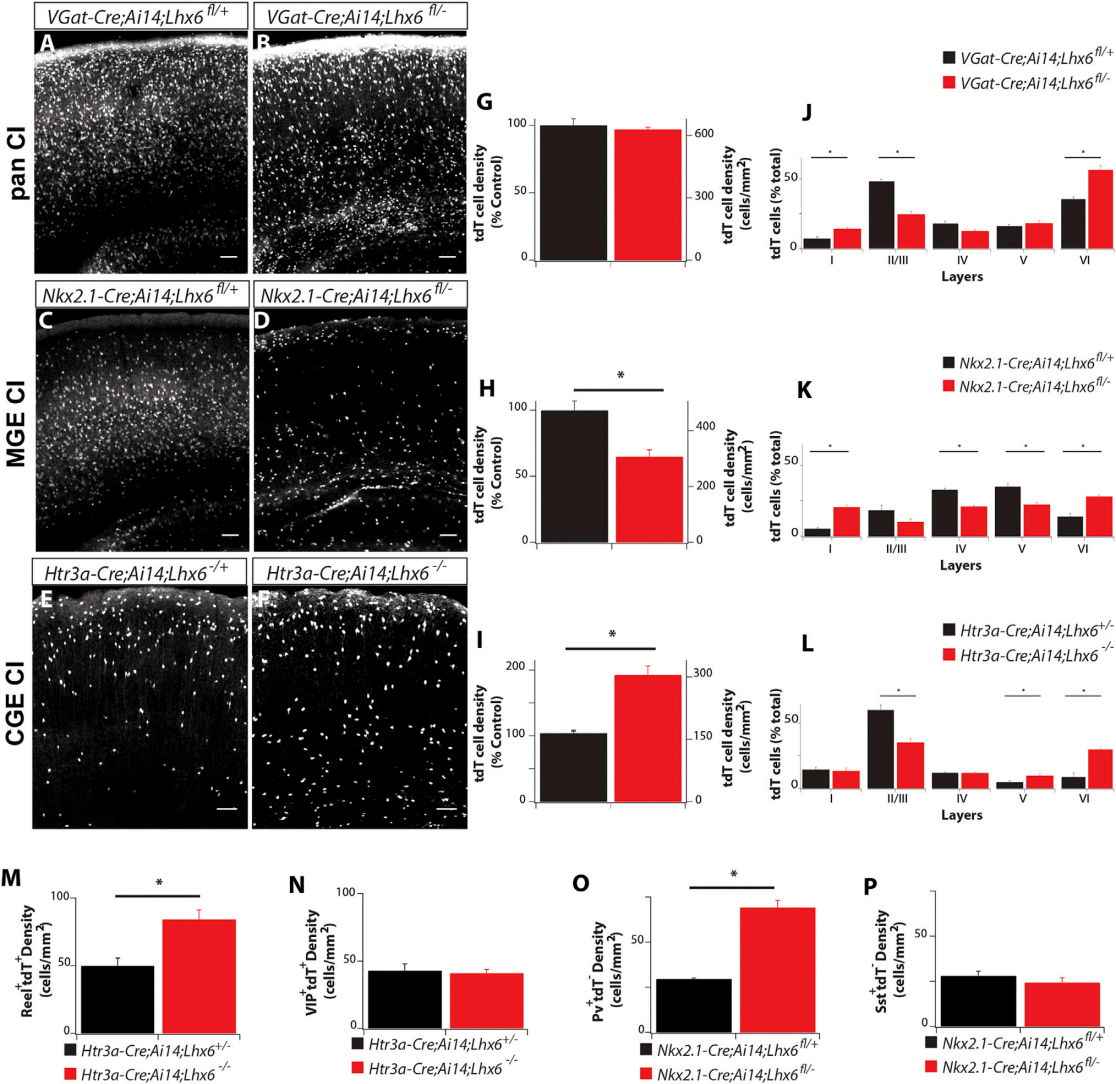
Fate Mapping Reveals Reduced Viability of *Lhx6*-Deficient MGE CIs and Increased Survival of CGE CIs (A–F) tdT^+^ (white signal) CIs in sections from the somatosensory cortex of *Lhx6* control (Ctrl; A, C, and E) and mutant (Mut; B, D, and F) P18 mice fate-mapped using different Cre driver lines, as indicated. Shown are the entire CI population (A and B), MGE-derived (C and D), and CGE-derived (E and F). (G–I) Quantification (raw densities, right axis; normalized to the average control level, left axis) of tdT^+^ cell density in somatosensory cortices for all CIs (G), MGE (H), and CGE (I) derived CIs. (J–L) Quantification of the distribution of tdT^+^ cells across cortical layers for all CIs (J), MGE (K), and CGE (L) derived CIs. (M–P) Quantification of cell densities of CGE-derived Reelin^+^ (M), CGE-derived VIP^+^ (N), non-MGE-derived Pv^+^ (O), and non-MGE-derived Sst^+^ cells (P). See also Figure S1 for characterization of the *Lhx6*^*fl*^ allele. Similar results were observed in the motor cortex (Figure S2). See Figure S3 for characterization of non-MGE derived Pv^+^ cells in *Lhx6* mutants. In all figures, data are expressed as mean ± SEM. Statistical significance was evaluated using Student’s t test, unless otherwise stated. ^∗^p < 0.05. Scale bars are 100 μm.

Using general (*Gad1*) and subtype-specific (Pv and Sst) markers for cortical inhibitory neurons, we and others have reported that mice homozygous for null mutations of *Lhx6* have a reduced number of MGE-derived Sst^+^ and Pv^+^ CI subpopulations but that the total number of GABAergic neurons in the neocortex and hippocampus remains unchanged (Liodis et al., 2007, Zhao et al., 2008). We reasoned that this could result from two different scenarios: either *Lhx6* activity is necessary for the expression of late differentiation MGE markers, or *Lhx6* is required for the survival of MGE-derived CIs, and a compensatory increase of non-MGE-derived CIs in mutants maintains the total number of CIs. To unequivocally distinguish between these two possibilities, we combined gene targeting with genetic fate mapping. Generation of a novel conditional allele of *Lhx6* (*Lhx6*^*fl*^) in the mouse (Supplemental Experimental Procedures; Figures S1A and S1B) and introduction into the *Lhx6*^*fl/−*^ genetic background of the Cre-dependent fluorescent reporter *Rosa26-tdTomato* (tdT, Ai14; Madisen et al., 2010) allowed us to use Cre drivers for cell-type-specific *Lhx6* ablation and simultaneous fate mapping of the mutant lineages. To validate the novel *Lhx6*^*fl*^ allele, we first used *VgatCre* (Vong et al., 2011) to delete *Lhx6* from all CI precursors. Consistent with the phenotype of *Lhx6*-null mutants, the population of Pv^+^ and Sst^+^ CIs was dramatically reduced in post-natal day 18 (P18) *VgatCre;Ai14;Lhx6*^*fl/−*^ mice relative to *VgatCre;Ai14;Lhx6*^*fl/+*^ controls (Figures S1C–S1F), whereas the total number of tdT^+^ cells remained unchanged (Figures 1A, 1B, and 1G). Next we used the *Nkx2.1Cre* transgenic driver (Kessaris et al., 2006) to specifically track Lhx6-deficient MGE-derived CIs. As expected, the percentage of tdT^+^ CIs co-labeled with antibodies against Lhx6, Pv, Sst, and Reelin was dramatically reduced in *Nkx2.1Cre;Ai14;Lhx6*^*fl/−*^ mice relative to *Nkx2.1Cre;Ai14;Lhx6*^*fl/+*^ controls (Figures S1G–S1L). Furthermore, we observed that the cortex of *Nkx2.1Cre;Ai14;Lhx6*^*fl/−*^ mice contained a small number of tdT^+^ cells co-expressing VIP or Sp8, markers that normally are associated with CGE-derived CIs and are absent from their MGE-derived counterparts (Figures S1M and S1N; Vogt et al., 2014). Notably, the overall number of tdT-expressing cells in the cortex of P18 *Nkx2.1Cre;Ai14;Lhx6*^*fl/−*^ mice was significantly smaller relative to controls (Figures 1H and S2A), suggesting that, in addition to its well-established role in CI subtype specification, *Lhx6* is also required for the survival of MGE-derived CIs.

The reduced number of tdT^+^ MGE-derived CIs observed in *Nkx2.1Cre;Ai14;Lhx6*^*fl/−*^ mice (Figures 1C, 1D, and 1H and S2A) in conjunction with the nearly normal number of GABAergic interneurons in the cortex of either *Lhx6*-null mice (Liodis et al., 2007, Zhao et al., 2008) or mice with pan-CI deletion of *Lhx6* (*VgatCre;Ai14;Lhx6*^*fl/–*^; Figure 1G), suggests that non-MGE-derived CI lineages compensate for the specific loss of MGE-derived CIs. To examine this possibility we fate-mapped CGE-derived CIs in *Lhx6*^*−/−*^ mice using the CGE-specific Cre driver *Htr3aCre* (http://www.gensat.org; Supplemental Experimental Procedures) and the Ai14 (tdT) reporter. We observed increased representation of CGE-derived tdT^+^ cells in the cortex of *Lhx6* mutant mice (*Htr3aCre;Ai14;Lhx6*^*−/−*^) relative to controls (*Htr3aCre;Ai14;Lhx6*^*+/−*^) (Figures 1E, 1F, and 1I and S2D). Therefore, the size of the CGE-derived CI population in the mammalian cortex is not pre-determined but can be modulated to compensate for the loss of MGE-derived interneurons in the cortex. Although the total number of CIs is not affected by the *Lhx6* deletion, the radial distribution of CIs is severely disrupted in *Lhx6* mutants, with CIs congregating along the upper and deeper layers of the cortex (Figure 1J; Liodis et al., 2007, Zhao et al., 2008). Specifically, MGE-derived CIs in *Lhx6* mutants are lost in the middle layer (IV), whereas the most obvious increase in CGE-derived CIs occurs in deeper layers (V and VI) (Figures 1K and 1L). This observation argues against a local survival signal that compensates for CI loss within cortical layers but, rather, suggests that CI number is modulated by a more global signal that operates across the cortex.

The majority of CGE-derived CIs can be accounted for by two functionally and molecularly distinct sub-populations marked by the expression of either VIP or Reelin (Fishell and Rudy, 2011, Kepecs and Fishell, 2014, Lee et al., 2010). Immunostaining of *Lhx6* mutant brain sections from CGE-labeled CI mice (*Htr3aCre;Ai14;Lhx6*^*−/−*^) using these subtype markers showed that only the Reelin^+^ subset increased in number, whereas the VIP^+^ subpopulation remained unchanged (Figures 1M and 1N and S2E and S2F). Interestingly, ablation of *Lhx6* from MGE lineages also resulted in altered representation of CI subtypes originating outside of the ganglionic eminences (Gelman et al., 2011). Thus, in *Nkx2.1Cre;Ai14;Lhx6*^*fl/−*^ mice, the number of tdT^−^Pv^+^ interneurons (which partly originate from the POA; Figures S2G–S2N) increased relative to controls (*Nkx2.1Cre;Ai14;Lhx6*^*fl/+*^), whereas the number of tdT^−^Sst^+^ CIs remained unchanged (Figures 1O and 1P and S2B and S2C). Based on a previous characterization of the Nkx2.1Cre line used here, some MGE-derived CIs fail to express Cre recombinase (mostly a subpopulation expressing Sst (Fogarty et al., 2007)) and can, therefore, represent MGE-derived cells that still express Lhx6 (Figures 1O and 1P and S2B and S2C). However, we found no differences in the number of GFP^−^Lhx6^+^ cells in control and mutant brains (Figure S1O), suggesting that the size of this “escapee” population does not increase in mutant brains and, therefore, cannot be responsible for the increase in tdT^−^Pv^+^ cells in mutants. These findings suggest that the compensatory responses of CIs to *Lhx6* ablation are subtype-specific and occur across different lineages.

### Reciprocal Changes in Apoptosis of CGE and MGE CIs in *Lhx6* Mutants

**Figure 2:**
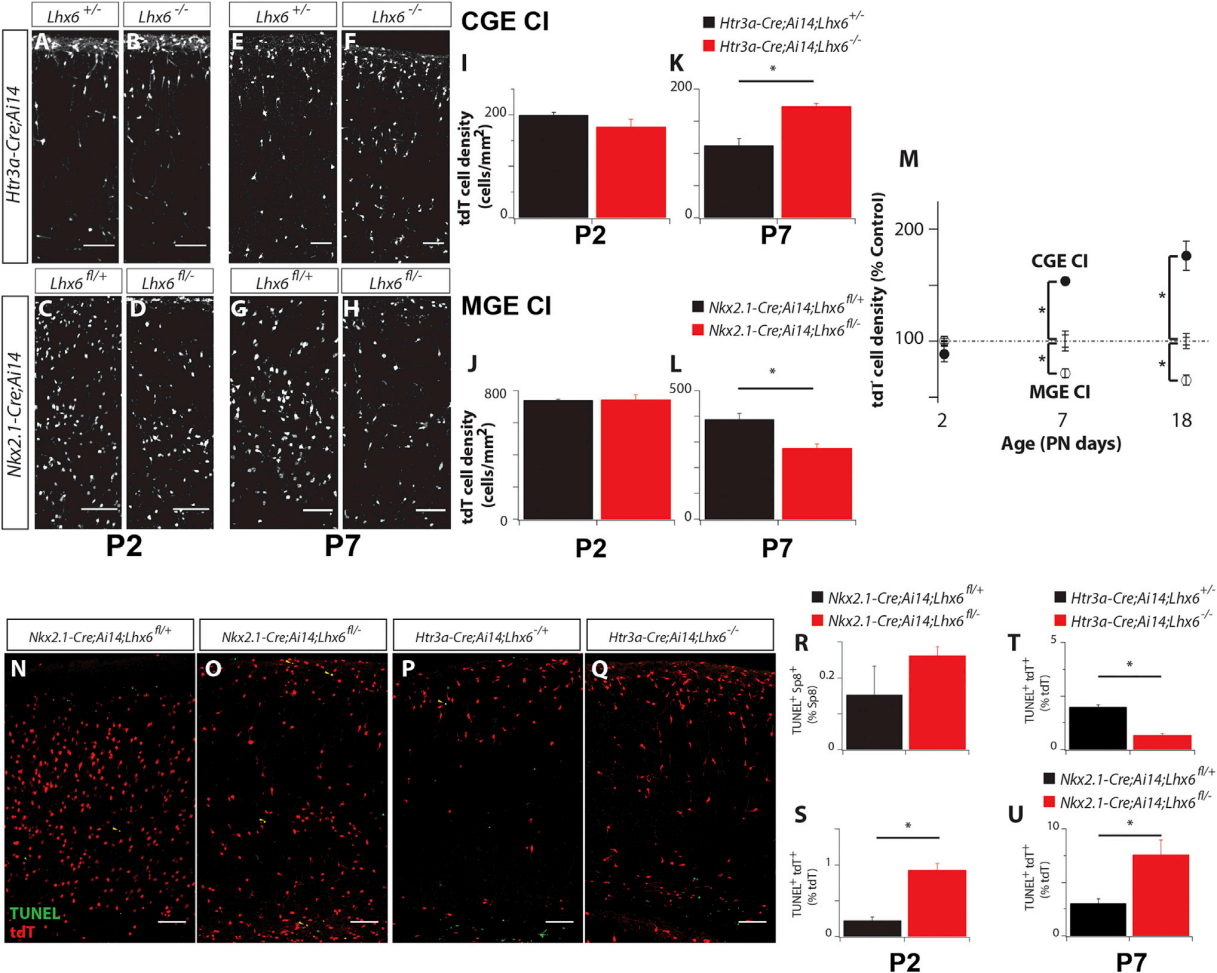
Reduced Apoptosis of CGE CIs Compensates for the Loss of *Lhx6*-Deficient MGE Counterparts Shown are changes in the number of CI subtypes in *Lhx6* control and mutant mice during early post-natal development. (A–H) tdT-expressing CIs in cortical sections from P2 (A–D) and P7 (E–H) *Lhx6* control (A, C, E, and G) and mutant (B, D, F, and H) mice. tdT expression identifies CGE-derived (A, B, E, and F) and MGE-derived CIs (C, D, G, and H), respectively. (I–L) Quantification of the distribution of tdT^+^ cell density in the cortex of P2 (I and J) and P7 (K and L) for CGE (I and K) and MGE (J and L) derived CIs. (M) Summary of changes in density of CGE (closed circles) and MGE (open circles) CIs in *Lhx6* mutant cortices relative to *Lhx6* controls (dotted line) at P2, P7, and P18. (N–Q) Representative cortical sections from P7 somatosensory cortices showing fate-mapped CIs (tdT^+^ in red) and TUNEL^+^ (green) cells. Yellow arrowheads indicate TUNEL^+^ fate mapped CIs. In (N) and (O), tdT expression represents MGE-derived CIs, whereas in (P) and (Q), tdT represents CGE-derived CIs. (R and S) Quantification of TUNEL^+^ cells for CGE-derived CIs (R; Sp8^+^TUNEL^+^) and MGE-derived CIs (S; tdT^+^TUNEL^+^) at P2 for control and *Lhx6* mutant cortices. (T and U) Quantification of TUNEL^+^ cells for CGE-derived (T) and MGE-derived (U) CIs undergoing apoptosis (tdT^+^TUNEL^+^) at P7 for control and *Lhx6* and mutant cortices. See Figure S4 for characterization of proliferation and migration of CI precursors in embryonic *Lhx6* mutant brains. Scale bars are 100 μm.

The increased representation of CIs originating outside of the MGE in the cortex of *Lhx6*-deficient mice could result from enhanced proliferation of their progenitors or reduced neuronal cell death during the critical postnatal window of apoptosis (Southwell et al., 2012, Yamaguchi and Miura, 2015). To distinguish between these possibilities, we first compared the number of proliferating progenitors (identified by pH3 immunostaining and 5-ethynyl-2'-deoxyuridine [EdU] uptake) within the ganglionic eminences of *Lhx6* mutant and control embryonic day 14.5 (E14.5)–E16.5 embryos. The number of pH3^+^ and EdU^+^ progenitors was similar between the two genotypes, suggesting that the mechanism(s) responsible for the increased representation of CGE-derived CIs in *Lhx6* mutants operates on post-mitotic interneuron precursors at later developmental stages (Figures S3A–S3J). In agreement with this hypothesis, there was no difference in the number of cells expressing Sp8 (a transcription factor expressed by non-MGE-derived CIs; Ma et al., 2012) in E16 control and mutant cortices (Figures S3K–S3N). However, we note that a small proportion of Sp8-expressing cells in mutant cortices is derived from the MGE (Figure S1N). In further support of this idea, the number of fate-mapped MGE- and CGE-derived CIs at P2 (a developmental stage that follows the completion of CI tangential migration but precedes the onset of apoptosis; Miyoshi and Fishell, 2011, Southwell et al., 2012) was similar between control and *Lhx6* mutant mice (Figures 2A–2D, 2I, and 2J). In contrast, 5 days later (at P7), we observed a decreased representation of MGE-derived CIs and a reciprocal increase of their CGE-derived counterparts (Figures 2E–2H, 2K, and 2L). The changes in the size of MGE- and CGE-derived CIs populations observed at P7 foreshadow those observed at P18 (Figures 1H and 1I and 2M) and, together, suggest lineage-specific modulation of the apoptotic programs of CIs by the *Lhx6* mutation.

To confirm this, we used terminal deoxynucleotidyl transferase dUTP nick end labeling (TUNEL) staining to quantify the extent of developmental cell death of MGE- and CGE-derived CIs at P2, to obtain an early readout of apoptosis in *Lhx6* mutants, and at P7, when expression of apoptotic markers in CIs is at its highest (Figure 2J; Southwell et al., 2012). TUNEL analysis at P2 revealed a marked increase in apoptosis of MGE-derived CIs (but not CGE-derived CIs) in *Lhx6*-null mutants even before the expected developmental apoptosis program begins (Figures 2R and 2S). A similar analysis in the cortex of *Nkx2.1Cre;Ai14;Lhx6*^*fl/−*^ mice at P7 also showed a significant increase in the number of tdT^+^TUNEL^+^ cells relative to control animals (*Nkx2.1Cre;Ai14;Lhx6*^*fl/+*^; Figure 2U). However, although CGE-derived CIs do not express *Lhx6*, the number of tdT^+^TUNEL^+^ double-positive CIs in the cortex of P7 *Htr3aCre;Ai14;Lhx6*^*−/−*^ mice was significantly reduced relative to control littermates (*Htr3aCre;Ai14;Lhx6*^*+/−*^; Figure 2U). We suggest that ablation of *Lhx6* during embryogenesis reduces the viability of MGE-derived CIs from an early stage in development. This reduction in the number of MGE-derived CIs triggers a compensatory and non-cell-autonomous decrease in the rate of developmental cell death in CGE-derived CIs during the normal period of CI apoptosis.

### Enhanced Survival of Wild-Type CIs Grafted into the *Lhx6* Mutant Cortex

**Figure 3:**
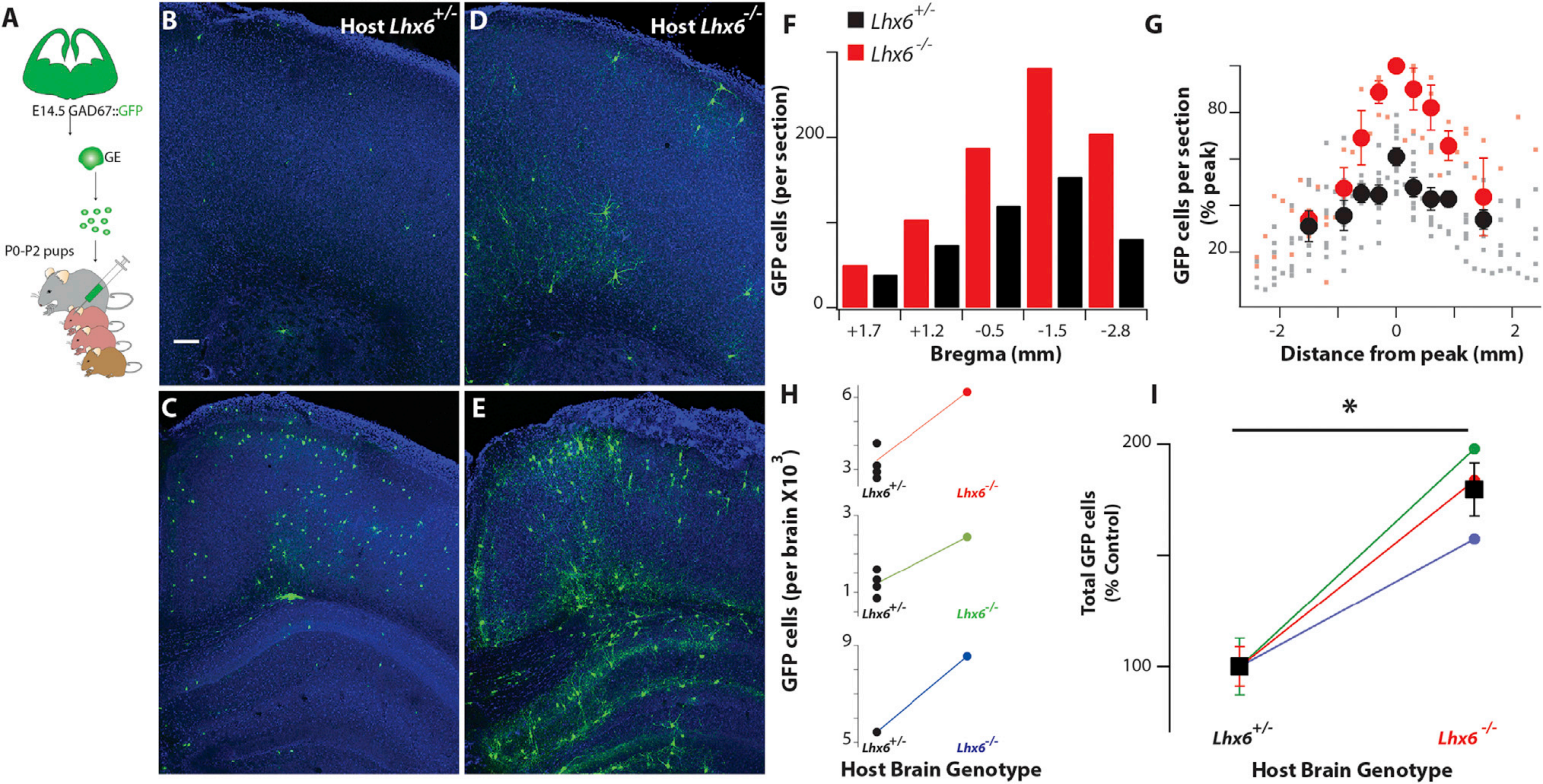
Cellular Microenvironment of *Lhx6* Mutant Forebrain Promotes Grafted CI Progenitor Survival (A) Schematic representation of the CI progenitor transplantation into the cortex of P0–P2 pups. (B–E) Coronal brain sections of Lhx6^+/−^ (B and C) and Lhx6^−/−^ (D and E) P16 mice transplanted at P0–P2 with wild-type GFP^+^ CI progenitors. The sections shown in (B) and (D) are more anterior to those shown in (C) and (E). (F) Distribution of surviving GFP^+^ CIs in the cortex of an *Lhx6*^*+/−*^ (black bars) and an *Lhx6*^*−/−*^ (red bars) littermate pair of P16 mice grafted with CI precursors at P0–P2 at different rostro-caudal levels. (G) Distribution of GFP^+^ cells was similar in all cortices analyzed, with a peak in the presumed injection site. Regardless of the rostro-caudal level quantified, more GFP^+^ cells were present in *Lhx6* mutant host cortices (red) than in their control littermates (black). (H and I) Quantification of surviving GFP^+^ CIs in the entire cortex of *Lhx6*^*+/−*^ and *Lhx6*^*−/−*^ P16 mice. In 3 independent experiments (H), the number of surviving GFP^+^ cells in the *Lhx6* mutant host cortices was higher than that observed in control littermates grafted with the same cell suspension. (I) Values (taken from H) for the mutants (*Lhx6*^*−/−*^, circles) are normalized to the average value in control littermates (*Lhx6*^*+/−*^; colored bars represent SEM for individual grafting experiments that correspond to those in H). The number of GFP^+^ cells found in *Lhx6*^*−/−*^ was 181% ± 13% higher in comparison with control littermates (p = 0.02, one sample t test, mean 100, n = 3 *Lhx6*^*−/−*^ and 10 *Lhx6*^*+/−*^, minimum of 500 cells counted per brain). See Figure S5 for morphological characterization of wild-type GFP^+^ CI progenitors grafted in *Lhx6* mutant brains. Scale bars are 100 μm.

To explore the possibility that the cortical microenvironment of *Lhx6* mutants can rescue CIs from apoptosis, we transplanted GFP^+^ CI precursors isolated from the basal forebrain of GAD-GFP E14.5 embryos (Tamamaki et al., 2003) into the pallium of neonatal (P0–P1) *Lhx6*^*−/−*^ pups and their control (*Lhx6*^*+/−*^) littermates (Figure 3A). Multiple morphologically mature GFP^+^ CIs were observed throughout the cortex at P16 in both control and mutant animals (Figures 3B–3F). No differences were observed in the spatial distribution of grafted CIs in the host cortices (Figures 3F and 3G). However, a subset of grafted CIs in mutant cortices showed striking morphologies, with consistently larger somata and dendritic arbors, a finding that mirrored the unusually large size of endogenous POA-derived Pv^+^ CIs observed in *Lhx6* mutant mice (Figure S4).

The majority of grafted CI precursors are eliminated by BAX-dependent apoptosis within 2 weeks of transplantation (Southwell et al., 2012), a feature that recapitulates the timeline of programmed cell death of endogenous CIs. Consistent with this idea, we found that the number of transplanted GFP^+^ CIs in the cortex of P16 *Lhx6*^*−/−*^ mice was higher relative to that in *Lhx6*^*+/−*^ littermate controls. These results were consistent across three independent experiments, regardless of the number of grafted interneurons (181% ± 13% of control, p = 0.02, n = 3 litters; Figures 3H and 3I), confirming the notion that the microenvironment of the host brain can modulate the survival of interneurons in the cortex.

### Transcriptomic Analysis of Lhx6-Deficient Brains/Lineages

**Figure 4:**
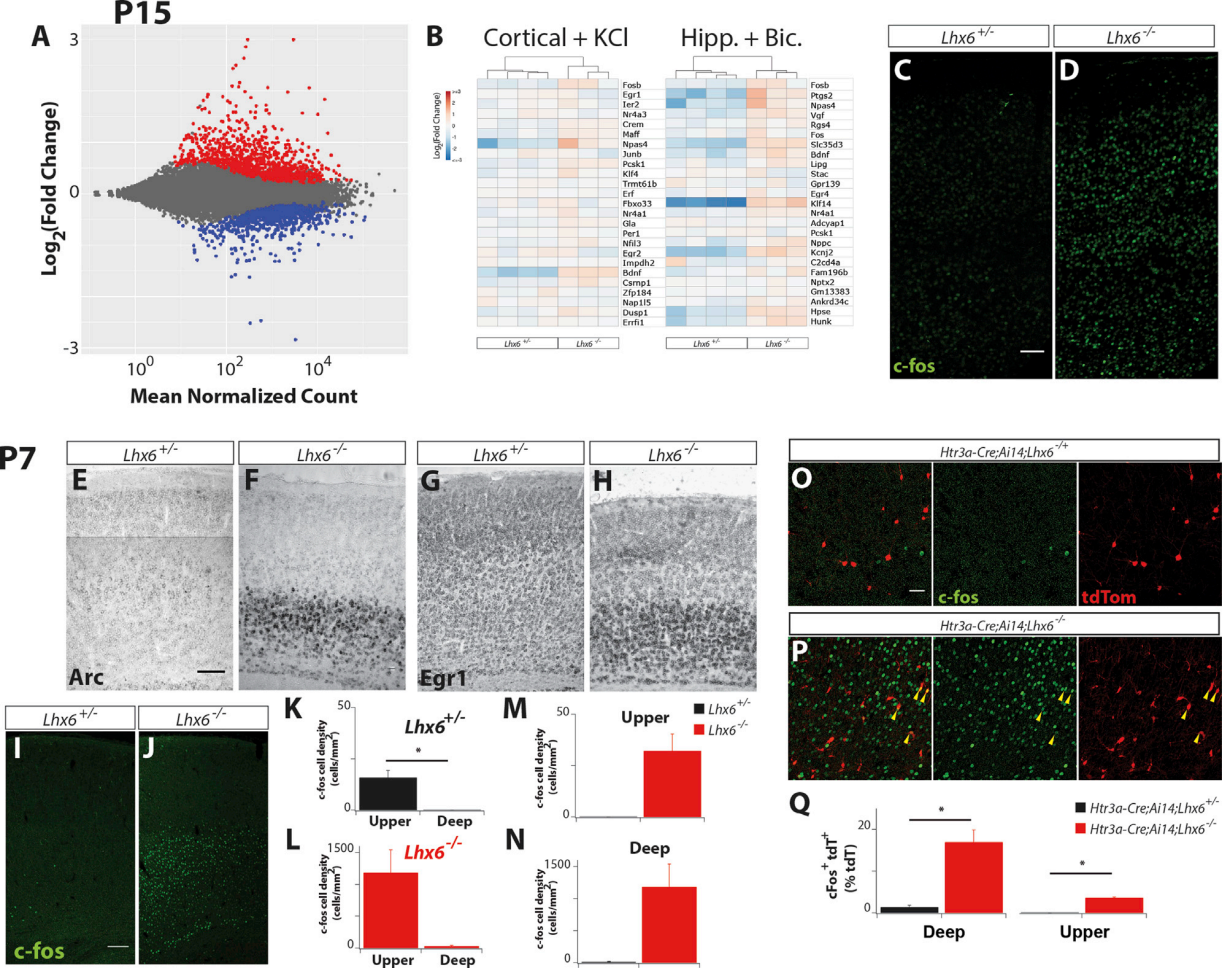
Molecular Analysis of *Lhx6* Mutant Forebrains Reveal Widespread Upregulation of Activity-Dependent Genes (A) MA plot summarizing the results of the differential expression analysis between *Lhx6*^*+/−*^ and *Lhx6*^*−/−*^ P15 forebrains. Significantly upregulated genes are shown in red (977 genes), whereas significantly downregulated genes are shown in blue (730 genes). Significance was set as a false discovery rate of ≤ 0.05. (B) Hierarchical clustering of *Lhx6*^*+/−*^ and *Lhx6*^*−/−*^ forebrain samples using activity-regulated genes. Clusters were generated using the expression levels of the 25 most significantly upregulated genes in either cortical cultures treated with KCL + FPL64176 (Qiu et al., 2016) or hippocampal cultures treated with bicuculline (Yu et al., 2015). Genes upregulated by either treatment are similarly upregulated upon deletion of *Lhx6*. (C and D) Coronal sections from the somatosensory cortex of *Lhx6*^*+/−*^ (C) and *Lhx6*^*−/−*^ (D) P15 mice immunostained for cfos (green). (E–H) *In situ* hybridization of somatosensory cortex sections from *Lhx6*^*+/−*^ (E and G) and *Lhx6*^*−/−*^ (F and H) P7 mice with either *Arc* (E and F) or *Egr1* (G and H) riboprobes shows immediately early gene upregulation in the bottom layers of mutant brains. (I and J) Coronal sections of the somatosensory cortex of *Lhx6*^*+/−*^ (I) and *Lhx6*^*−/−*^ (J) P7 mice immunostained for cfos (green); note the upregulation of cfos expression, particularly in the lower half of the cortex. (K–M) Quantification and distribution of cfos^+^ cell density in upper (M) and deep (N) areas of *Lhx6*^*+/−*^ (K) and *Lhx6*^*−/−*^ (L) cortices. (O and P) High-magnification images of the bottom half of the somatosensory cortex of *Htr3a-Cre;Ai14;Lhx6*^*+/−*^ (O) and *Htr3a-Cre;Ai14;Lhx6*^*−/−*^ (P) P7 brains immunostained for cfos (green). Yellow arrowheads show examples of cfos-expressing CGE-derived tdT^+^ cells. (Q) Quantification of the distribution of cFos+ tdT+ (CGE-derived CIs) in the cortex. See Table S2 for a summary description of mRNA sequencing results for the genes highlighted in (A) and Table S3 for a summary of results of the RT^2^ profiler PCR array for CGE-derived CIs isolated by fluorescence-activated cell sorting (FACS) from P7 cortices. Scale bars are 100 μm for (C)–(J) and 50 μm for (O) and (P).

To provide insight into the mechanisms by which the cortical microenvironment of *Lhx6* mutants controls the survival of grafted CI progenitors, we used RNA sequencing (RNA-seq) to compare the global transcriptome of the forebrain dissected from control (*Lhx6*^*+/−*^) and mutant (*Lhx6*^*−/−*^) mice at P15, a stage at which apoptosis of grafted CIs is at its highest. Differential gene expression analysis identified 1,707 genes that were significantly upregulated (977) or downregulated (730) (Figure 4A) in mutant relative to control littermates (summary results are presented in Table S2). In addition to *Lhx6* (which was absent from mutant samples), *Sst* and *Pv* were among the top downregulated genes in *Lhx6* mutants, in agreement with our immunocytochemistry data (Figures S1J and S1K). We also detected increased expression of genes normally expressed in CGE CIs in mutant cortices, such as Vip and Npy. Interestingly, inspection of the list of upregulated genes identified several—including *Bdnf* (Hartmann et al., 2001), *Npas4* (Bloodgood et al., 2013), *Fosb* (Eagle et al., 2015), and *Npy* (Gall et al., 1990)—whose expression is induced by neuronal activity. Gene set enrichment analysis using the hypergeometric test showed that the genes significantly modulated in *Lhx6*-deficient forebrains significantly overlap with genes altered in pyramidal neurons following chronic increases in activity (either through 48 hr inhibition of ionotropic GABA receptors in hippocampal neurons [p < 10^−59^; Yu et al., 2015] or through activation of L-type voltage-dependent Ca^2+^ channels in cortical neurons [p = 2.3 × 10^−3^; Qiu et al., 2016]). In fact, hierarchical clustering based on the expression of the top 25 genes upregulated by chronic activity clearly distinguished between control and mutant samples (Figure 4B). Finally, the expression of the activity-dependent gene *cfos* (Cohen and Greenberg, 2008) was highly upregulated throughout the cortex of *Lhx6* mutants (Figures 4C and 4D). These transcriptomic results provide a molecular confirmation of increased network activity in the cortex, as would be expected for brains where the development of MGE-derived CIs is compromised (Batista-Brito et al., 2009, Neves et al., 2013).

Next we compared the expression of neuronal activity markers between control and *Lhx6*-deficient cortices at P7, a postnatal stage characterized by the highest rate of interneuron cell death (Southwell et al., 2012). This analysis showed a dramatic upregulation of a number of immediate-early genes, including the activity-regulated cytoskeleton-associated protein *Arc* (Tzingounis and Nicoll, 2006), the early growth response protein *Egr1* (French et al., 2001), and *cfos*, in the cortex of *Lhx6*-deficient mice relative to controls (Figures 4E–4J). Interestingly, overexpression of the activity-dependent markers was observed mostly in the deeper layers of the cortex (Figures 4K and 4L), which also showed the highest increase in the number of CGE-derived CIs (Figures 1E, 1F, and 1L). Our gene expression analysis demonstrates a correlation between increased immediate-early gene expression, which is reflective of enhanced network activity, and enhanced survival of CIs in the cortex of *Lhx6* mutant mice.

Next we compared the expression of immediate-early gene markers specifically in CGE-derived CIs labeled with tdT in *Htr3aCre;Ai14;Lhx6*^*−/−*^ versus *Htr3aCre;Ai14;Lhx6*^*+/−*^ mice at P7. First, *cfos* immunostaining showed that the number of cFos^+^tdT^+^ neurons (yellow arrowheads in Figure 4P) was increased in the *Lhx6*-deficient cortex relative to controls, suggesting increases in the activity of CGE CIs (Figures 4O–4Q). To further characterize such changes, we employed RT^2^ profiler PCR array technology (Supplemental Experimental Procedures) to compare the expression of a panel of known activity-associated genes in CGE-derived CIs isolated by flow cytometry from the brain of P7 *Htr3aCre;Ai14;Lhx6*^*−/−*^ and *Htr3aCre;Ai14;Lhx6*^*+/−*^ mice (Table S3). Among the genes upregulated (>1.5-fold change) in CGE CIs from *Lhx6*-deficient brains were a number of genes associated with increased activity levels, including two members of the EGR family (*Egr2* and *Egr3;* DeSteno and Schmauss, 2008, Li et al., 2007), the neurotrophic factor *bdnf* (Hartmann et al., 2001), as well as genes implicated in growth factor signaling, such as insulin-like growth factor 1 (*Igf1*; Mardinly et al., 2016) and the nerve growth factor receptor (*Ngfr*; Meeker and Williams, 2015). These factors and their receptors play a crucial role in the control of neuronal numbers and dendritic growth. Together, these findings identify increased activity of CIs as a potential mechanism that drives their enhanced survival in hyperactive cortical networks.

### Cell-Autonomous Increase in the Activity of CIs Enhances Survival

**Figure 5:**
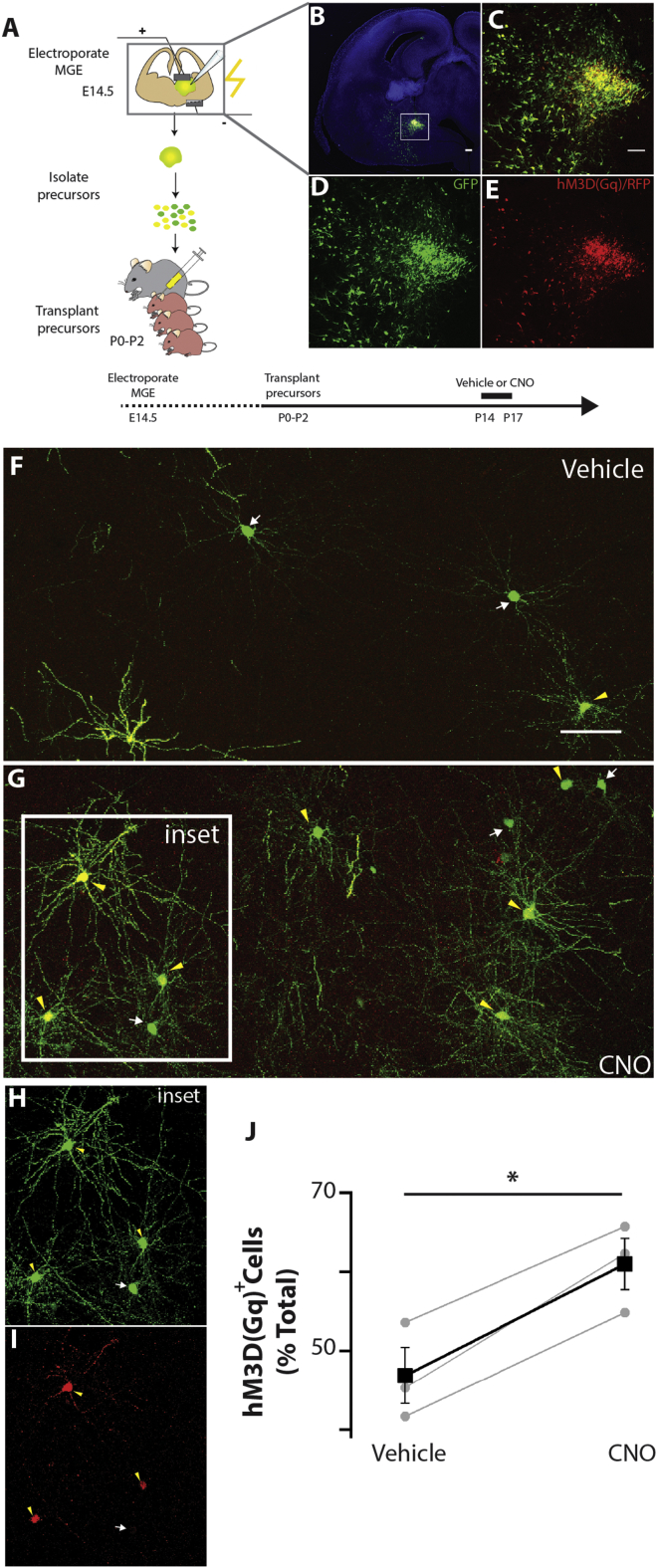
Cell-Autonomous Depolarization of CIs Enhances Their Survival (A) Schematic representation of the brain acute slice electroporation, grafting, and vehicle/CNO administration protocol. Drug administration was targeted to coincide with the peak of apoptosis of transplanted CI progenitors. (B–E) Coronal section from an E14.5 embryo brain transfected with the CAG:IRES:GFP (pGFP) and CAG:hM3D(Gq):IRES:RFP (pDREADRFP) plasmids and cultured for 12 hr (B). The boxed area is magnified to show the expression of both fluorescent reporters (C), GFP only (D), and RFP only (E). (F–I) Representative sections from the somatosensory cortex of P17 mice grafted at P0–P2 with CI precursors transfected with the pGFP and pDREADDRFP plasmids and injected with either vehicle (F) or CNO (G). Yellow arrowheads identify cells expressing both plasmids, whereas green arrows indicate cells expressing GFP only (H and I). The boxed region in (G) is magnified to reveal the expression of GFP (H) and RFP (I). (J) Quantification of RFP^+^ cells found in the forebrain of P17 mice transplanted at P0–P2 (normalized to the total GFP^+^ population). RFP^+^ (vehicle] = 47% ± 3%, CNO = 61% ± 3%, p = 0.01, Student’s paired samples t test, n = 3 vehicle and 3 CNO, minimum of 150 cells counted per brain. See Figure S6 for analysis of the effects of CNO administration in hM3D(Gq)-expressing cells. Scale bars are 200 μm for (B) and 100 μm for (C)–(I).

To directly test whether CI survival is regulated by neuronal activity in a cell-autonomous manner, we transplanted CI precursors expressing designer receptors exclusively activated by designer drugs (DREADDs) and modulated their activity by administering the appropriate ligands (Urban and Roth, 2015). Specifically, the MGE of E14.5 embryos was co-electroporated with a bi-cistronic expression vector encoding the hM3D(Gq) DREADD and red fluorescent protein (RFP) and a control plasmid encoding GFP. Transfected CIs were mechanically dissociated, and the resulting cell suspension was grafted in the cortex of P0–P1 wild-type mice. Because only a fraction of electroporated (GFP^+^) neurons co-expressed hM3D(Gq) (RFP^+^) (Figures 5A–5E), the GFP^+^RFP^–^ population served as an internal control for the effect of DREADD ligands. Indeed, administration of the DREADD ligand clozapine-N-oxide (CNO) selectively increased the activity of transfected GFP^+^RFP^+^ cells (Figure S5). Importantly, CNO treatment (administered twice daily from P14–P17) resulted in an increase in the fraction of GFP^+^RFP^+^ (yellow arrowheads) relative to GFP^+^RFP^–^ (white arrows) cells compared with vehicle-administered littermates (Figures 5F–5J), suggesting that enhanced activity is sufficient to protect CIs from programmed cell death in an otherwise normal brain. Our data provide evidence that neuronal activity modulates the number of CIs in the cortex in a cell-autonomous manner.

## Discussion

Distinct physiological mechanisms, collectively referred to as homeostatic plasticity, operate in the nervous system to maintain or restore the balance between excitation and inhibition, even after considerable disruption of network dynamics (Turrigiano, 2012). For such “acute” mechanisms to be effective, it is essential that all physiologically relevant cellular compartments achieve a critical size and correct composition during development. How the output of developmental programs that specify the number and subtypes of neurons matches the functional requirements of mature neuronal circuits remains unclear. Here we provide evidence that modulation of programmed cell death during a critical early postnatal period is a regulatory mechanism that controls, in a homeostatic manner, the number of GABAergic interneurons in the mammalian cortex. Our experiments highlight a critical interplay between the physiological state of the network and its cellular units and suggest a feedback mechanism that fine-tunes the size of the CI population to stabilize brain activity.

Early stages of neural development are often characterized by proliferative expansion of progenitors that create a surplus number of neurons that are later eliminated by apoptosis. For example, the size of motor neuron and sympathetic neuron pools is largely determined during development by the availability of limiting amounts of retrograde pro-survival signals supplied by relevant peripheral targets (Davies, 2003, Oppenheim, 1991). However, the neurotrophic factor paradigm cannot adequately explain the regulation of apoptosis in most regions of the CNS where alternative pathways have been implicated (Dekkers et al., 2013). *In vivo* and *in vitro* studies have demonstrated that survival of cortical PNs is enhanced by network activity and that N-methyl-D-aspartate (NMDA) receptor-mediated synaptic currents modulate rates of apoptosis (Blanquie et al., 2017, Ikonomidou et al., 1999; reviewed by Bell and Hardingham, 2011). In addition, apoptosis of adult-generated neurons, such as olfactory bulb interneurons and dentate gyrus granule cells, can be dramatically influenced by the activity of the mature networks into which they integrate (Bovetti et al., 2009, Corotto et al., 1994, Mu et al., 2015, Petreanu and Alvarez-Buylla, 2002, Rochefort et al., 2002, Tashiro et al., 2006), probably through a cell-autonomous mechanism (Lin et al., 2010). Contrasting these studies, Southwell et al. (2012) demonstrated recently that, in rodents, a large fraction of CIs (∼40%) are eliminated by a program of apoptosis that is intrinsic to this cell lineage. Our genetic lineage tracing experiments in *Lhx6* mutants argue against a rigid and intrinsically determined program of apoptosis of CI progenitors and suggest a considerable degree of developmental plasticity driven ultimately by the physiological state of the network. This view is supported by the preferential survival of either wild-type CI progenitors grafted into the hyperactive cortex of *Lhx6*-deficient animals (Figure 3) or by chemogenetically activated MGE-derived CIs grafted into the cortex of wild-type animals (Figure 5). Together with the modulation of endogenous CI numbers in the *Lhx6* mutant cortex, our findings suggest an overarching mechanism for the control CI number in the pallium during development, whereby inhibitory interneurons monitor the activity of their local environment and adjust the level of apoptosis in a cell-autonomous manner. Interestingly, the enhanced apoptosis of immature CIs observed in response to pharmacological inhibition of NMDA receptors (Roux et al., 2015) suggests that excitatory glutamatergic neurotransmission may play a role in this regulatory pro-survival response. In line with this view, cell autonomous increases in the activity of CIs improves their survival through a calcineurin-mediated pathway (Priya et al., 2018).

Other studies have also shown apparent compensatory forms of plasticity in response to the loss of CI subtypes. Deletion of the *Lhx6*-dependent effector gene *Sox6* in postmitotic immature interneurons was associated with a dramatic decrease in the number of Pv^+^ and Sst^+^ interneurons but no change in the total number of CIs (Azim et al., 2009, Batista-Brito et al., 2009). Although an increase of NPY^+^ interneurons was reported in these studies, the mechanisms that maintain the total number of CIs in *Sox6* mutants remain unclear. Also, conditional inactivation of the orphan nuclear receptor *Nrf1* (*COUP-TFI*) in interneuron progenitors resulted in a decreased number of CR^+^ and VIP^+^ CIs and a concomitant increase of PV- and NPY-expressing subtypes without affecting the total number of GABAergic interneurons in the cortex (Lodato et al., 2011). Although this compensation was thought to result from enhanced proliferation of CI progenitors, it is possible that changes in apoptosis also contribute to this phenotype. Our data, in agreement with Priya et al. (2018), show that regulation of CI apoptosis is subtype-specific. For example, among the *Lhx6*-independent CGE-generated CIs, only Reelin^+^ neurons increase in number following *Lhx6* deletion (Figures 1M and S2). The subtype-specific response of CIs to activity is not solely restricted to apoptosis because recent studies have shown that silencing CGE-derived interneurons results in defects in radial migration, cell morphology, and synaptic development of Reelin^+^ but not VIP^+^ CIs (De Marco García et al., 2011, De Marco García et al., 2015). Although it has also been suggested that MGE-derived CIs are more sensitive to hyperpolarization than CGE-derived CIs (Close et al., 2012), we find that CGE-derived CIs strongly modulate their levels of apoptosis in *Lhx6* mutants (Figure 2T). However, whether this is a result of increases in high network activity (Figures 4E–4L) or by a different molecular pathway still remains to be established. Further analysis of the set of candidate genes we identified in CGE-derived CIs isolated by flow cytometry may help clarify the mechanisms behind the cell-specific modulation of apoptosis in CIs. Our results also shed new light on the many functions of *Lhx6* in the development of MGE-derived CIs. Here, we show that MGE CIs lacking *Lhx6* have increased rates of apoptosis from early post-natal stages (certainly by P2; Figures 2S and 2U), with only a small fraction of CIs undergoing a previously observed change to a CGE fate (Figures S1M and S1N; Vogt et al., 2014). However, it is unclear what role the remaining MGE-derived mutant cells play during development. Our results suggest that the majority of these cells fail to integrate into a functional network, as evidenced by their abnormal accumulation in the cortical margins and high rates of apoptosis.

Our results provide evidence for a simple mechanism that controls the number of inhibitory interneurons in the cortex. We propose that the temporal overlap between developmental programs that dictate the size (and thus the functional output) of the CI complement and the emerging activity of cortical networks allows for the engagement and cross-regulation of the two processes until an optimal activity set point is attained. Several preclinical models of CI-based cell therapies have been established for the treatment of epileptic seizures (Alvarez Dolado and Broccoli, 2011, Southwell et al., 2014, Tyson and Anderson, 2014). Our present data argue that increased activity levels in the host brain, typically observed in epileptic encephalopathy mouse models (Batista-Brito et al., 2009, Hedrich et al., 2014), or increased activity in transplanted CIs will provide favorable conditions for the survival of grafted CI progenitors. Characterizing the pro-survival patterns of neuronal activity and identifying the CI subtypes best suited for transplantation may improve the effectiveness of these nascent therapies.

## Experimental Procedures

### Animals

All procedures involving mice were approved by the ethical review panel at King’s College London (KCL) and National Institute for Medical Research in accordance with the United Kingdom Animals (Scientific Procedures) Act (1986). A conditional *Lhx6* allele (*Lhx6*^*fl*^) was generated via homologous recombination using a targeting construct in which loxP sites were placed in non-coding regions just 5′ to coding exon 1b and 3′ to coding exon 3 (Figure S1A). The Tg(Nkx2-1-cre)1Wdr (Mouse Genome Informatics [MGI]:3761164, shortened here as *Nkx2.1-Cre*; Kessaris et al., 2006), Slc32a1^tm2(cre)Lowl^ (MGI:5141270, shortened here as *VGat-Cre*; Vong et al., 2011), Tg(Htr3a-cre)NO152Gsat (MGI:5435492, shortened here as *Htr3a-Cre*, generated by The Gene Expression Nervous System Atlas [GENSAT] Project, The Rockefeller University, New York), and Gt(ROSA)26Sor^tm14(CAG-tdTomato)Hze^ (MGI:3809524, shortened here as Ai14; Madisen et al., 2010), Lhx6^tm2Vpa^ (MGI:3702518, shortened here as *Lhx6*^*−*^; Liodis et al., 2007), and *Lhx6*^*fl*^ animals were maintained on a mixed background and genotyped as described previously. Both male and female mice were used in all experiments.

### GE Cell Transplantations

Both medial and caudal ganglionic eminences were dissected from E14.5 Gad1^tm1.1Tama^ (MGI:3590301, shortened here as *GAD67-GFP*; Tamamaki et al., 2003) heterozygote embryos, dissociated as previously described (Du et al., 2008), and the resulting cell suspension was grafted into the cortices of control (*Lhx6*^*+/−*^) and mutant (*Lhx6*^*−/−*^) neonatal pups (P0–P1). One single injection was made into the cortex of each pup. The same needle was used for all injections to pups of the same litter, and between injections, the needle was inspected to verify that the same cell suspension volume was injected. Grafted animals were transcardially perfused at P16. Only litters containing at least one mutant and one control mouse were analyzed, and values for mutants were normalized to the average number found in the control littermates, injected with the same cell suspension.

### MGE Electroporation and Cell Transplantations

*Ex vivo* electroporation of MGE in embryonic brain slices (E14) was conducted as described previously (Stühmer et al., 2002). Twelve hours after electroporation of a mixture of pCAGGS-hM3D(Gq)-IRES (internal ribosome entry site)-RFP and pCAGGS-IRES-GFP plasmids, the MGE regions with the strongest GFP signal were dissociated as described previously (Du et al., 2008). The resulting cell suspension was grafted into neonatal (P0–P1) cortices of wild-type mice (6 injections per brain/3 per hemisphere) as described above. Cohorts of littermates grafted with the same cell suspension were divided in two groups: one group was injected intraperitoneally twice per day (every 12 hr) with 1 mg/kg CNO (Tocris Bioscience) (diluted in vehicle: 0.5% DMSO containing saline), whereas a control group was injected with vehicle only from P14 until P17 (one injection only at P17). Mice where then transcardially perfused within 1 hr from the last injection, and dissected brains were processed for immunohistochemistry. Only experiments where GFP^+^ cells were identified in at least one animal from each group were analyzed.

### Statistical Methods

All mean, SE, and statistical tests were calculated using standard statistical routines in Igor Pro or Excel. n is taken as the number of animals. In Table S1, we present detailed information of the statistical tests used to assess significance.

## Data and Software Availability

The accession number for the RNA-seq results reported in this paper is GEO: GSE108161.

## Supplemental Information

Document S1. Supplemental Experimental Procedures, Figures S1–S5, and Tables S1 and S3

Table S2. Summary Information for Genes with Significantly Upregulated and Downregulated Expression in Lhx6-Null Mutant Forebrains at P15 by mRNA Sequencing Analysis, Related to Figure 4

Document S2. Article plus Supplemental Information
