# Supplementary material for: Modulation of Apoptosis Controls Inhibitory Interneuron Number in the Cortex
Source: Cell Rep. 2018 Feb 13;22(7):1710–21. doi: 10.1016/j.celrep.2018.01.064 (PMC6230259; doi:10.1016/j.celrep.2018.01.064)
Supplement: Document S1. Supplemental Experimental Procedures, Figures S1–S5, and Tables S1 and S3 [file mmc1.pdf]

Cell Reports, Volume *22*

Supplemental Information

Modulation of Apoptosis Controls Inhibitory Interneuron Number in the Cortex

Myrto Denaxa, Guilherme Neves, Adam Rabinowitz, Sarah Kemlo, Petros Liodis, Juan Burrone, and Vassilis Pachnis

Supplemental Experimental Procedures:

Lhx6 conditional mice and genotyping: A conditional *Lhx6* allele was generated via homologous recombination using a targeting construct in which loxP sites were placed in non-coding regions just 5' to coding exon 1b, and 3' to coding exon 3 (Figure S1 A). The 5' homology is made up from a 3.1 kb *XbaI–Sac*I fragment containing the 5' upstream region and the first exon (1a) of *Lhx6*, whereas the 3' homology is made up from a 5.4 kb *ApaI–NheI* fragment. For the *Lhx6* targeting vector, the 2 kb genomic fragment between the homology regions is replaced by a 4 kb cassette, containing the following: (1) the 1b, 2 and 3 coding exons flanked by loxP sites (2) the neomycin resistance gene under the control of the phosphoglycerate kinase (PGK) promoter (PGK-Neo) flanked by FRT sites. Targeting constructs were linearized and electroporated into E14Tg2A embryonic stem (ES) cells. Targeted clones were identified and analyzed in detail by Southern blotting using the 5' and 3' external probes indicated in Figure S1B. Germline transmission of the mutant alleles was achieved using standard protocols. The phenotypic analysis presented was performed on animals from which the PGK-Neo cassette was removed by crossing founder $Lhx6^{Fl/+}$ animals with the *Tg(ACTFLPe)9205Dym* transgenic line (MGI:2448985, Rodriguez et al., 2000). Deletion was confirmed using Southern blotting analysis. For the maintenance of the $Lhx6^{Fl}$ colony, we performed PCR using the following primers: F:5'-CTCGAGTGCTCCGTGTGTC-3', R:5'-GGAGGCCCAAAGTTAGAACC-3'.

Animals: Animals were bred and housed in accordance with the United Kingdom Animals (Scientific Procedures) Act (1986). The *Tg(Nkx2-1-cre)1Wdr* (MGI:3761164, shortened here as *Nkx2.1Cre,* Kessaris et al., 2006), $Slc32a1^{tm2(cre)Lowl}$ (MGI:5141270, shortened here as *VGatCre*, Vong et al., 2011), *Tg(Htr3a-cre)NO152Gsat* (MGI:5435492, shortened here as *Htr3aCre*, generated by The Gene Expression Nervous System Atlas –GENSAT - Project, The Rockefeller University - New York), $Gt(ROSA)26Sor^{tm14(CAG-tdTomato)Hze}$ (MGI:3809524, shortened here as *Ai14*, Madisen et al., 2010). $Lhx6^{tm2Vpa}$ (MGI:3702518, shortened here as $Lhx6^{-}$, Liodis et al., 2007) and $Lhx6^{fl}$ animals were maintained on a mixed background and genotyped as described previously. To generate productive crosses, *Cre-positive;*$Lhx6^{+/-}$ males were mated with $Lhx6^{fl/fl}$*;Ai14* or $Lhx6^{+/-}$*;Ai14* females and the resulting littermates were analyzed.

Illumina RNA-Seq library preparation, sequencing and analysis: Illumina RNA-Seq libraries were made with 1 μg of total RNA according to the manufacturer's protocol. The only deviation from the protocol was to use the e-gel clone well system (Invitrogen) for fragment size selection. Total RNA was extracted from the forebrain of P15 mice. Each sample was composed of two animals. Four samples for $Lhx6^{+/-}$ and three samples for $Lhx6^{-/-}$ mouse brains were collected and further analysed. 36 base pair single end sequencing was undertaken using an Illumina GA IIx DNA sequencer. FASTQ reads were trimmed with CutAdapt (http://journal.embnet.org/index.php/embnetjournal/article/view/200/479) to remove adapter contamination and low quality sequence. Reads were aligned to the Ensembl GRCm38.84 version of the mouse transcriptome using Bowtie2 (Langmead and Salzberg, 2012). Expected gene counts were extracted from the alignments using RSEM (Li and Dewey, 2011). Differential expression analysis was then perfomed using DESeq2 package (Love et al., 2014). Significance was assigned to genes with an adjusted p-value of <= 0.05. Summary information on differentially epressed genes is provided in Table S2. Significance enrichment of differentially expressed genes with gene lists obtained from other studies were identified using the hypergeometric test. To correct for multiple-hypothesis-testing a false-discovery rate was calculated from the resultant p-values. Heatmaps: The log2-fold-change expression, of each gene in each sample, was calculated relative to the mean expression of the gene across all samples. Hierarchical clustering of the samples was performed on the euclidean distances between the log2-fold-change values for each sample.

Quantification of CGE-derived CI transcripts: For quantification of transcripts in CGE-derived CIs, P7 brains were dissociated (papain dissociation system, Worthington, Lorne) and tdT^{+} cells were isolated by flow cytometry (FACS ARIAII, Becton-Dickinson; typically, a yield of 60,000 cells). Total RNA was purified (RNeasy microKit, Qiagen) and subjected to reverse transcription (RT^{2} First Strand, Qiagen) and quantitative PCR according to manufacturer's instructions (RT^{2} Profiler PCR plasticity pathway-focused gene expression array, Qiagen). Results presented in Table S3 represent were obtained from two *Htr3aCre;Ai14;*$Lhx6^{-/-}$ and two *Htr3aCre;Ai14;*$Lhx6^{+/-}$ samples.

Expression constructs: Full length cDNA for hM3D(Gq) from plasmid pAAV-hSyn-HA-hM3D(Gq)-IRES-mCitrine (Addgene, 50463), was cloned into a modified pCAGGS-IRES-RFP vector (a gift from Francois Guillemot - CRICK), resulting in pCAGGS-hM3D(Gq)-IRES-RFP. The pCAGGS-IRES-GFP was a gift from James Briscoe (CRICK).

GE cell transplantations: Both medial and caudal ganglionic eminences were dissected from E14.5 $Gad1^{tm1.1Tama}$ (MGI:3590301, shortened here as GAD67::GFP, Tamamaki et al., 2003) heterozygote embryos,

dissociated as previously described (Du et al., 2008), and the resulting cell suspension was grafted into the cortices of *Lhx6* control and mutant neonatal pups (P0-P1). One single injection has been performed into the cortex of each pup, by using the micro-injector unit set-up from the VEVO injection system (VisualSonics). Each injection (69 nl) has been performed at a slow injection rate (23nL/sec) with needles according to the recommended dimensions of the manufacturer. The same needle has been used for all injections to pups of the same litter, and between each injection the needle has been inspected to verify the same cell suspension volume was injected. Grafted animals were transcardially perfused at P16, and dissected brains were processed for immunohistochemistry. Only litters containing at least one $Lhx6^{-/-}$ (mutant) and one $Lhx6^{+/-}$ (control) mouse were analysed, and values for mutants were normalized to the average number found in the control littermates, injected with the same cell suspension.

MGE electroporation and cell transplantations: *Ex vivo* electroporation of MGE in embryonic brain slices (E14) was conducted as described previously (Stuhmer et al., 2002). Twelve hours after electroporation of a mixture of pCAGGS-hM3D(Gq)-IRES-RFP and pCAGGS-IRES-GFP plasmids, MGE regions with the strongest GFP signal were dissociated as previously described (Du et al., 2008). The resulting cell suspension was grafted into neonatal (P0-P1) cortices of wild type mice (6 injections per brain/3 per hemisphere) as described above. Cohorts of littermates grafted with the same cell suspension were divided in two groups: one group was injected intraperitoneally twice per day (every 12 hrs) with 1mg/kg CNO (Tocris Bioscience) (diluted in vehicle - 0.5% DMSO containing saline); while a control group was injected with vehicle only, from P14 until P17 (one injection only at P17). Mice where then transcardially perfused within 1 hr from the last injection, and dissected brains were processed for immunohistochemistry. Only experiments where GFP^+ cells were identified in at least one animal from each group were analysed.

In utero elecroporations: In utero electroporation was carried out using a protocol adapted from (Saito and Nakatsuji, 2001). Briefly, timed-pregnant wild type CD-1 female mice were anesthetized with a mix of oxygen-isoflurane before the abdomen was opened and the uterine horns exposed. The DNA solution was injected into the lateral ventricle of E14.5 embryos using a glass micropipette. Approximately 0.5 microliters of a solution containing Tris-HCl (10 mM), ethylenediaminetetraacetic acid (EDTA, 1 mM), Fast green dye (0.5% w/v) and a mixture (3:1 molar ratio) of pCAGGS-hM3D(Gq)-IRES-RFP and pCAGGS-IRES-GFP plasmids (total DNA concentration ≈ 1 mg/ml) was injected. Five square electric pulses (40 V, 50 ms) were passed at 1 s intervals using a square-wave electroporator (CUY21EDIT, NEPA GENE Co.). At P21 a group of mice received an intraperitoneal injection of 1mg/kg CNO (Tocris Bioscience) (diluted in vehicle - 0.5% DMSO containing saline) and were transcardially perfused and processed for immunohistochemichestry with GFP, RFP and cfos antibodies. A group of control mice did not receive CNO injections.

Immunostaining: For immunostaining on brain sections from P16 mice or older, animals were transcardially perfused with 4% PFA and brains were post-fixed overnight (O/N). Vibratome (60 or 100 μm) sections were permeabilized with PBT [0.5% Triton X-100 in PBS (0.5% PBT)] for 1 hr at room temperature (RT), blocked in 10% FCS in PBT (0.3% Triton X-100 in PBS; 2 hrs; RT), and incubated with primary antibodies diluted in blocking solution at 4°C, O/N. After 3 washes with PBT, sections were incubated with secondary antibodies diluted in blocking solution at RT for 2 hrs, washed in PBT, and mounted using Vectashield (Vector) medium. For immunostaining on brain sections from embryos (E14.5, E16.5) or P2/P7 mouse pups, dissected brains were fixed in 4% PFA in PBS at 4°C, O/N. Cryostat sections (14 μm) were permeabilized in 0.1% Triton X-100 in PBS (0.1%PBT) for 5 min, and processed as above. The following antibodies were used: rabbit polyclonal anti-cFos (Calbiochem; 1/10.000), mouse monoclonal anti-cFOS (Santa-Cruz, sc-166940; 1/500), rabbit polyclonal anti-GFP (Invitrogen; 1/1000), rat monoclonal anti-GFP (Invitrogen; 1/1000), mouse monoclonal anti-GFP (Invitrogen; 1/1000), rabbit polyclonal anti-Lhx6 (Lavdas et al., 1999; 1/250–1000), rabbit polyclonal anti-Parvalbumin (Swant, PV25; 1/1000), goat polyclonal anti-Parvalbumin (Swant, PV213; 1/1000), rabbit anti-PH3 (Millipore, 05-636; 1/200), rabbit polyclonal anti-RFP (Abcam; 1/500), mouse monoclonal anti-Reelin (Millipore, MAB5364; 1/500), rabbit polyclonal anti-Sox6 (Abcam, AB30455; 1/4000), goat polyclonal anti-Sp8 (Santa-Cruz, sc-104661; 1/1000), rat monoclonal anti-SST (Millipore, MAB354; 1/1000), rabbit polyclonal anti-VIP (Immunostar, 20077; 1/1000). Secondary antibodies used were as follows: Alexa Fluor 488-conjugated donkey anti-mouse, anti-rat, anti-goat and anti-rabbit and Alexa Fluor 568-conjugated donkey anti-mouse, anti-rat, anti-goat and anti-rabbit (all from Invitrogen; all 1/500).

In situ hybridization histochemistry (ISHH): ISHH was carried out essentially as described (Schaeren-Wiemers and Gerfin-Moser, 1993). The Arc-specific and Egr1-specific riboprobes were a gift from Dr. Tahebayashi.

EdU (5-ethynyl-2'-deoxyuridine) injection/staining: A stock solution of 10mg/ml EdU (Invitrogen, E10187) was prepared in DPBS (Life technologies, 14190-094). Pregnant females were injected intraperitoneally with 3μl/g weight of each mouse. To study the fraction of cells in the S-phase of the cell cycle, pregnant females were

injected 1 hour prior to embryo harvest. Brain cryosections from E14.5 or E16.5 embryos were first processed for immunohistaining (PH3) and then EdU detection according to the manufacturer's protocol.

Tunel (terminal deoxynucleotidyl transferase-mediated dUTP nick end-labelling) assay: In order to detect cells that are undergoing apoptotic cell death, we used the ApopTag Green *In Situ* Apoptosis Detection Kit (Invitrogen, S7111) and followed manufacturer's instructions. Briefly, immunostained sections were fixed for 10 minutes with 4% PFA, washed with PBS and post-fixed further with an EtOH/Acetic Acid (2:1) mix. Upon equilibration with the appropriate buffer, sections were incubated with TdT enzyme mix for 1hour at 37°C. The reaction was then stopped with STOP buffer, sections were washed and slides were incubated with fluorescein conjugated anti-DIG mix in a dark humidified chamber, for 30 minutes at room temperature. Slides were then washed thoroughly with PBS and mounted with Vectashield-DAPI mounting medium.

Image analysis: Cell counting for all post-natal stages was performed from coronal sections in the cortical region. Images were acquired using a confocal microscope (×20 magnification), taking care for all pairwise comparisons to be acquired in the same imaging session with the same acquisition settings. Cells were manually identified in columnar regions spanning the pial-white matter extent of the cortex across different bregma levels between +2 and -3 mm (as defined in Paxinos et al., 2001) or equivalent regions in the early post-natal stages (shown in Figures 2A-2L). Bregma regions were closely matched for each pairwise comparison. No obvious changes in the reported changes were detected between different bregma regions (e.g. results in Figures 1H-1P are similar to Figure S2). tdT^{+} cell numbers in Figures 1G-1I, S2A and S2D were divided by the total surface area counted (measured in ImageJ) to obtain cell densities (expressed as cells per mm^2). For layer distribution analysis (Figure 1J- 1L), the cortical column was divided in the different cortical layers, identified by DAPI nuclear staining. Numbers for each layer are expressed as a fraction of all the cells counted. Some measurements were normalized to the average density observed for control animals in each pairwise comparison. For analysis of specific CI subpopulations (Figures 1M-1P, S2B, S2C, S2E and S2F, separate channels were acquired and markers were initially assessed independently, and were later combined to assess co-localization using the ImageJ software.. Essentially similar methods were used for analysis of apoptosis of fate mapped CI populations (Figures 2S-2U) and characterization of the MGE CI population in *Lhx6* conditional mutants (Figures S1I-S1N), except that cell numbers were normalized to the size of the tdT^{+} population. For Figures 1G-1P analysis was restricted to the primary somatosensory cortex (as defined in Paxinos et al., 2001), while for Figure S2 countings were performed in the primary motor cortex. For Figures 2I-2M, and S1G-S1M countings were performed for at least 4 different matched bregma levels and measurements were averaged to obtain cell densities for each animal. For analysis of survival of grafted GAD67::GFP^{+} CIs (Figure 3I), cells present in the cortex in all sections were counted. Numbers for each litter (independently shown in Figure 3 H) were normalized to the average number found in the $Lhx6^{+/-}$ animals, a minimum of 500 cells were counted per brain. For analysis of grafted cell dispersion (Figure 3G), cell counts in each section (for all littermates transplanted with that same cell suspension) to the maximum number of cells observed in the the litter (always a mutant section), We then sorted the sections for each brain by distance (in the anterior-posterior axis) to the section with maximum observed cell count for that brain. For analysis of survival of grafted CIs transfected with hM3D(Gq) expressing constructs (Figure 5J) GFP^{+} cells present in the cortex in all sections were counted and assessed for RFP expression. Numbers of double positive cells ($RFP^{+}GFP^{+}$) were expressed as a fraction of total GFP^{+} population for each brain, a minimum of 150 cells were counted per brain. In the graph lines connect markers corresponding to animals grafted with the same cell suspension and given different treatments (vehicle or CNO). For analyses of soma size (Figure S4), an oval shaped region of interest (ROI) was drawn just inside the soma region of cells that were judged to be in focus. The area size of the ROIs was measured. Area sizes were measured from at least three different brains in matched bregma levels. For analysis of nuclear cfos expression (Figure S5) images were acquired at 40X magnification. The cfos channel was analysed independently, by drawing oval shaped ROIs inside the nuclear region of all nuclei present in the image, at the Z slice with highest fluorescent intensity. Average fluorescent intensity was measured for all ROIs in both the cfos channel and the RFP channel. Measurements in the cfos channel were divided in two groups based on RFP intensity. All cfos intensity measurements were normalized to the median intensity of the group with RFP expression below threshold. All imaging and analysis were done by an experimenter blind to the experimental condition. All values are presented as mean ± standard error of the mean. All results presented in Figures are presented in the Table S1, accompanied by the statistical test used to assess significance.

Supplemental References:

Du, T., Xu, Q., Ocbina, P.J., and Anderson, S.A. (2008). NKX2.1 specifies cortical interneuron fate by activating Lhx6. Development 135, 1559-1567.

Kessaris, N., Fogarty, M., Iannarelli, P., Grist, M., Wegner, M., and Richardson, W.D. (2006). Competing waves of oligodendrocytes in the forebrain and postnatal elimination of an embryonic lineage. Nat Neurosci 9, 173-179.

Langmead, B., and Salzberg, S.L. (2012). Fast gapped-read alignment with Bowtie 2. Nat Methods 9, 357-359.

Lavdas, A.A., Grigoriou, M., Pachnis, V., and Parnavelas, J.G. (1999). The medial ganglionic eminence gives rise to a population of early neurons in the developing cerebral cortex [In Process Citation]. J Neurosci 19, 7881-7888.

Li, B., and Dewey, C.N. (2011). RSEM: accurate transcript quantification from RNA-Seq data with or without a reference genome. BMC Bioinformatics 12, 323.

Liodis, P., Denaxa, M., Grigoriou, M., Akufo-Addo, C., Yanagawa, Y., and Pachnis, V. (2007). Lhx6 activity is required for the normal migration and specification of cortical interneuron subtypes. J Neurosci 27, 3078-3089.

Love, M.I., Huber, W., and Anders, S. (2014). Moderated estimation of fold change and dispersion for RNA-seq data with DESeq2. Genome Biol 15, 550.

Madisen, L., Zwingman, T.A., Sunkin, S.M., Oh, S.W., Zariwala, H.A., Gu, H., Ng, L.L., Palmiter, R.D., Hawrylycz, M.J., Jones, A.R., *et al.* (2010). A robust and high-throughput Cre reporting and characterization system for the whole mouse brain. Nat Neurosci 13, 133-140.

Paxinos, G., Franklin, K.B.J., and Franklin, K.B.J. (2001). The mouse brain in stereotaxic coordinates, 2nd edn (San Diego: Academic Press).

Rodriguez, C.I., Buchholz, F., Galloway, J., Sequerra, R., Kasper, J., Ayala, R., Stewart, A.F., and Dymecki, S.M. (2000). High-efficiency deleter mice show that FLPe is an alternative to Cre-loxP. Nat Genet 25, 139-140.

Saito, T., and Nakatsuji, N. (2001). Efficient gene transfer into the embryonic mouse brain using in vivo electroporation. Dev Biol 240, 237-246.

Schaeren-Wiemers, N., and Gerfin-Moser, A. (1993). A single protocol to detect transcripts of various types and expression levels in neural tissue and cultured cells: in situ hybridization using digoxigenin-labelled cRNA probes. Histochemistry 100, 431-440.

Stuhmer, T., Puelles, L., Ekker, M., and Rubenstein, J.L. (2002). Expression from a Dlx gene enhancer marks adult mouse cortical GABAergic neurons. Cereb Cortex 12, 75-85.

Tamamaki, N., Yanagawa, Y., Tomioka, R., Miyazaki, J., Obata, K., and Kaneko, T. (2003). Green fluorescent protein expression and colocalization with calretinin, parvalbumin, and somatostatin in the GAD67-GFP knock-in mouse. J Comp Neurol 467, 60-79.

Vong, L., Ye, C., Yang, Z., Choi, B., Chua, S., Jr., and Lowell, B.B. (2011). Leptin action on GABAergic neurons prevents obesity and reduces inhibitory tone to POMC neurons. Neuron 71, 142-154.

Figure S1

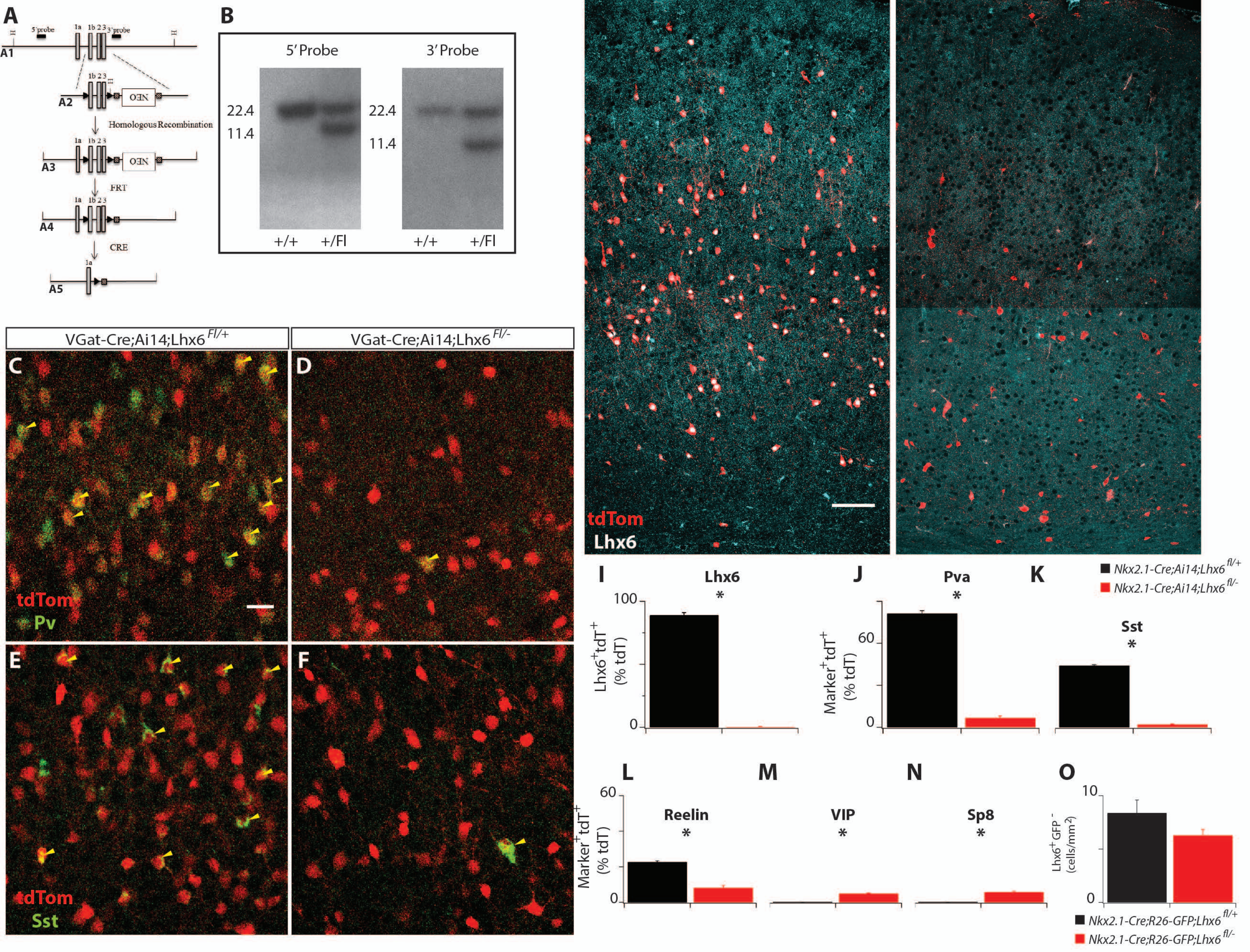
A
5'probe
3'probe
1a
1b
2
3
H
A1
A2
NEO
Homologous Recombination
A3
FRT
A4
CRE
A5
B
5' Probe
3' Probe
22.4
11.4
+/+
+/Fl
VGat-Cre;Ai14;Lhx6 Fl/+
VGat-Cre;Ai14;Lhx6 Fl/-
C
D
E
F
tdTom
Pv
Sst
Nkx2.1-Cre;Ai14;Lhx6 Fl/+
Nkx2.1-Cre;Ai14;Lhx6 Fl/-
G
H
Lhx6
I
J
K
Pva
Sst
Lhx6+tdT+ (% tdT)
Marker+tdT+ (% tdT)
100
60
0
Nkx2.1-Cre;Ai14;Lhx6 fl/+
Nkx2.1-Cre;Ai14;Lhx6 fl/-
L
M
N
O
Reelin
VIP
Sp8
10
Lhx6+GFP- (cells/mm2)
Nkx2.1-Cre;R26-GFP;Lhx6 fl/+
Nkx2.1-Cre;R26-GFP;Lhx6 fl/-

Figure S1: Generation and characterization of a conditional *Lhx6* allele (**A**) Schematic representation of the strategy used to generate the *Lhx6* ^{fl} allele by homologous recombination. A1 shows the targeted region of the wild-type *Lhx6* locus. Exons 1a, 1b (generated by alternative splicing), 2 and 3 as well as the probes used for Southern blot analysis are indicated. H, HindIII. A2 shows the targeting construct including exons 1b, 2 and 3 flanked by loxP sites and PGK-Neo positive selection cassette flanked by FRT sites. The targeted *Lhx6* allele following homologous recombination and removal of the PGK-Neo cassette (by FlpE-induced recombination of the FRT sites) is shown in A3 and A4, respectively. A5, following CRE-mediated recombination exons 1b, 2 and 3 are deleted. (**B**) Southern Blot analysis of HindIII digested genomic DNA from wild type (*Lhx6*$^{+/+}$) and *Lhx6*$^{fl/+}$ animals hybridized with 5' and 3' homology probes verifying the generation of the *Lhx6*^{fl} allele. (**C,F**) Coronal sections from the somatosensory cortex of P18 control *VGat-Cre;Ai14;Lhx6*$^{+/fl}$ (Ctrl; C,E) or mutant *VGat-Cre;Ai14;Lhx6*$^{fl/-}$ (Mut; **D,F**) littermates were immunostained with polyclonal antibodies against Pv (**C,D**) or Sst (**E,F**). Note a large fraction of CIs (tdT^{+}, in red) express Pv and Sst (arrowheads) in control, but not in mutant sections. (**G,H**) Cryosections from the somatosensory cortex of P18 control *Nkx2.1-Cre;Ai14;Lhx6*$^{+/fl}$ (Ctrl; **G**) or mutant *Nkx2.1-Cre;Ai14;Lhx6*$^{fl/-}$ (Mut; **H**) littermates were immunostained with a polyclonal Lhx6 antibody. The *Lhx6*^{fl} allele was recombined with the *Nkx2.1::Cre* transgene and recombination was followed using the Cre-dependent Ai14 (tdT) reporter. Lhx6 expression (blue) is undetectable in MGE CIs (red) in *Lhx6*$^{fl/-}$ mice. (**I**) Quantification of Lhx6^{+} MGE-derived CIs in Ctrl and Mut mice. (**J-N**) Quantification of the co-localization of MGE and CGE markers in Ctrl and Mut mice. Note the dramatic reduction of MGE specific CI markers and the appearance of residual expression of CGE-specific markers. (**O**) Quantification of Lhx6^{+} not expressing GFP (reporter of Cre recombination) in Ctrl and Mut mice. Scale bars: 50 microns (**C-F**), Data expressed as mean ± SEM. Statistical significance evaluated using Student's t-test, * denotes $p < 0.05$.

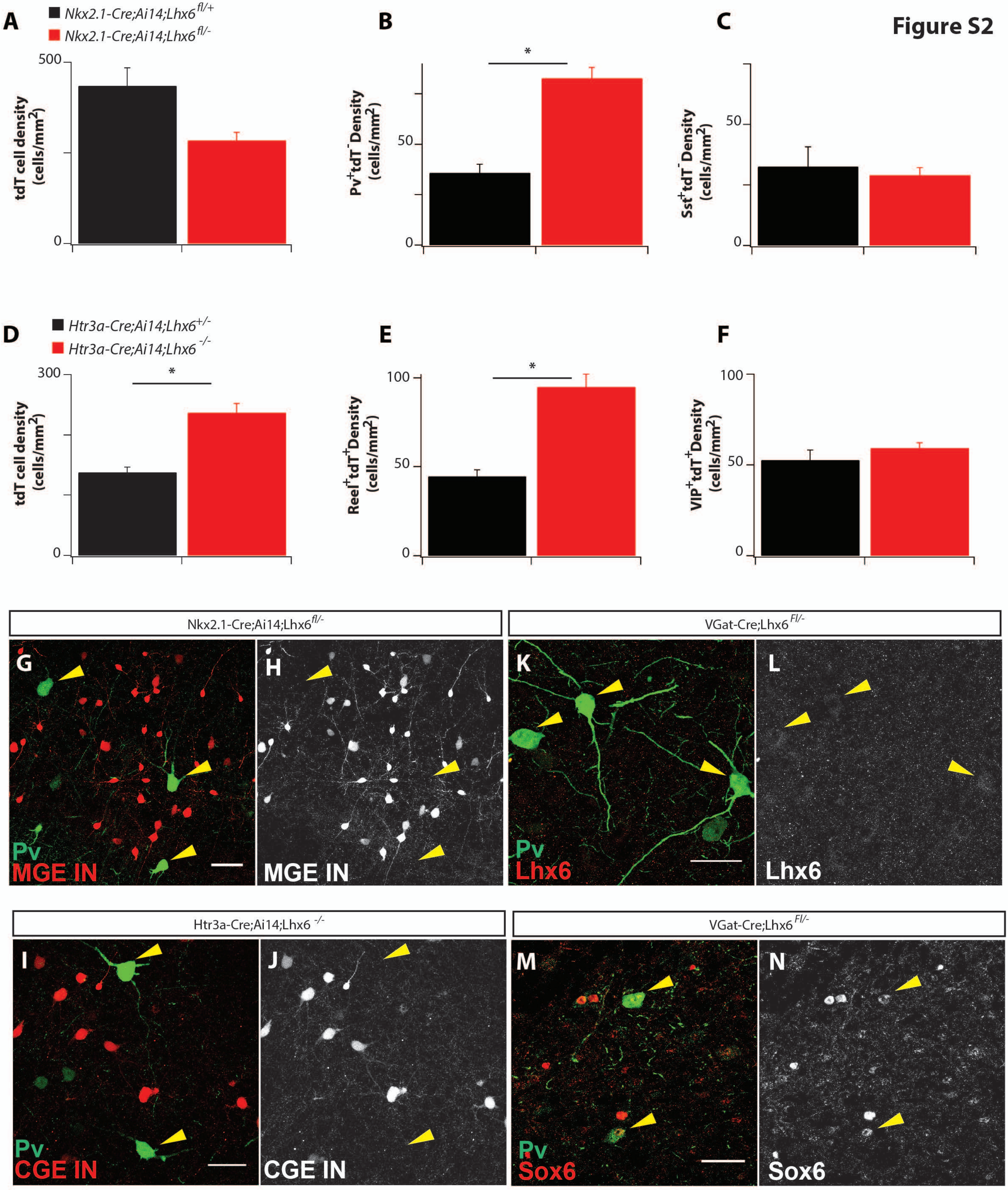

Figure S2
A
B
C
D
E
F
Nkx2.1-Cre;Ai14;Lhx6 fl/+
Nkx2.1-Cre;Ai14;Lhx6 fl/-
tdT cell density (cells/mm2)
Pv+tdT- Density (cells/mm2)
Sst+tdT- Density (cells/mm2)
Htr3a-Cre;Ai14;Lhx6+/-
Htr3a-Cre;Ai14;Lhx6 -/-
Reel+tdT+Density (cells/mm2)
VIP+tdT+Density (cells/mm2)
Nkx2.1-Cre;Ai14;Lhx6fl/-
VGat-Cre;Lhx6 Fl/-
Htr3a-Cre;Ai14;Lhx6 -/-
G
H
K
L
I
J
M
N
Pv
MGE IN
Lhx6
CGE IN
Sox6

Figure S2: Characterisation of CIs in mature (P18) Lhx6 control and mutant brains, related to Figure 1. Quantification of CI subtypes in the motor cortex of *Lhx6*$^{fl/+}$ (Ctrl) and *Lhx6*$^{fl/-}$ (Mut) mice. The data in panels **A-F** are from the same animals used for Figure 1. Specifically, **A, B, C, D, E** and **F** correspond respectively to panels **H, O, P, I, M** and **N** of Figure 1. Note that the changes observed in motor cortex are similar to those observed in the somatosensory cortex. Pv expressing cells in *Lhx6* null mutants do not derive from the MGE (**G-H**) or the CGE (**I**-**J**). (**G-J**) Coronal sections from the cortex of P18 *Nkx2.1-Cre;Ai14;Lhx6*$^{fl/-}$ (**G**) and *Htr3a-Cre;Ai14;Lhx6*$^{-/-}$ (**I**) mice immunostained for Pv (green). CIs derived from the MGE (**G**) or CGE (**I**) are tdT^{+} (red). In panels **H** and **J**, which correspond to **G** and **I** respectively, the lineage tracer is shown in white. It has been shown that a fraction of MGE derived CIs do not express Cre in the Nkx2.1-Cre line used in this study (Kessaris et al., 2006). However, Pv expressing in *Lhx6* mutants do not express Lhx6 (E-F), but express Sox6 at low levels, suggesting they originate in the POA (Gelman et al., 2011). (**K-N**) Coronal sections from P18 *Lhx6*$^{-/-}$ mice double immunostained for Pv (green) and Lhx6 (red in **K**) and Sox6 (red in **M**). In panels **L** and **N**, which correspond to **K** and **M** respectively, the Lhx6 and Sox6 signal is shown in white. Yellow arrowheads indicate Pv-expressing cells. Scale bars: 50 microns.

Figure S3

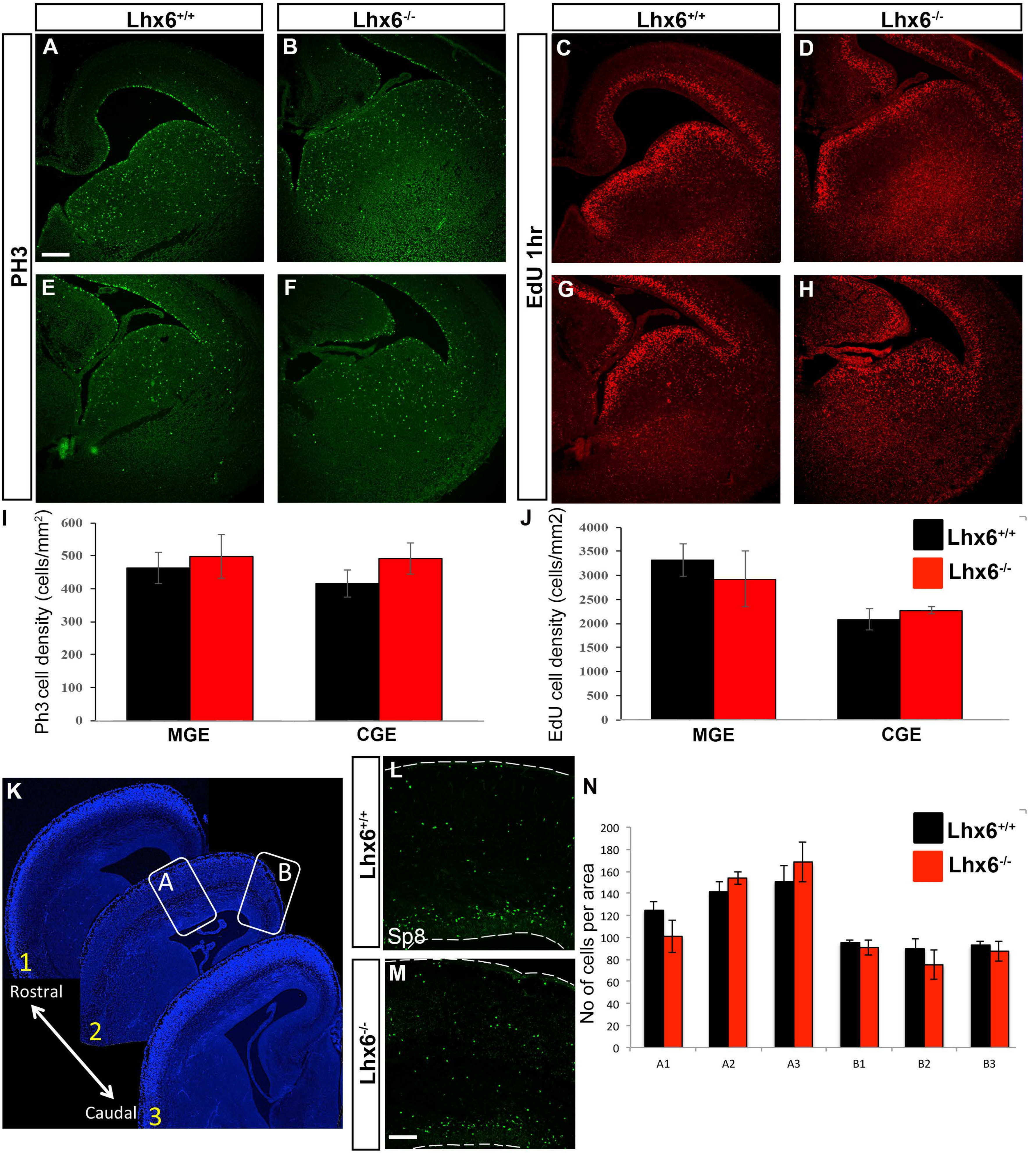
Lhx6+/+
Lhx6-/-
Lhx6+/+
Lhx6-/-
PH3
EdU 1hr
A
B
C
D
E
F
G
H
I
J
Ph3 cell density (cells/mm2)
600
500
400
300
200
100
0
MGE
CGE
EdU cell density (cells/mm2)
4000
3500
3000
2500
2000
1500
1000
500
0
Lhx6+/+
Lhx6-/-
K
A
B
1
2
3
Rostral
Caudal
L
M
Sp8
Lhx6+/+
Lhx6-/-
N
No of cells per area
200
180
160
140
120
100
80
60
40
20
0
A1
A2
A3
B1
B2
B3
Lhx6+/+
Lhx6-/-

Figure S3: No changes in proliferation or migration of CI precursors in *Lhx6* mutant embryos, related to Figure 2. (**A,B,E,F**) Coronal brain sections from E14.5 wild-type (**A,E**) and *Lhx6* mutant (**B**,**F**) mouse embryos immunostained for pH3. Sections shown in **A** and **B** are from the MGE level while sections shown in **E** and **F** are from the CGE level. (**C,D,G,H**) The set of sections shown in **A,B,E** and **F** imaged for EdU. (**I-J**) Quantification of results shown in (**A-H**). (**K**) DAPI staining of 3 different sections across the rostro-caudal axis of an E16.5 wild-type mouse brain, representing the areas analysed for Sp8 immunostaining. Boxes indicate the two areas (A and B) along the latero-medial axis used for $Sp8^+$ CI quantification shown in **N**. (**L, M**) $Sp8^+$ migrating interneurons (green) in cortical sections of the A2 brain area (**K**) from wild-type (**L**) and *Lhx6* mutant (**M**) E16.5 embryos. (**N**) Quantification of $Sp8^+$ CIs along different regions of $Lhx6^{+/+}$ (black) and $Lhx6^{-/-}$ (red) E16.5 mouse brains. Scale bars: 200 microns (A-H), 100 microns (L-M).

Figure S4

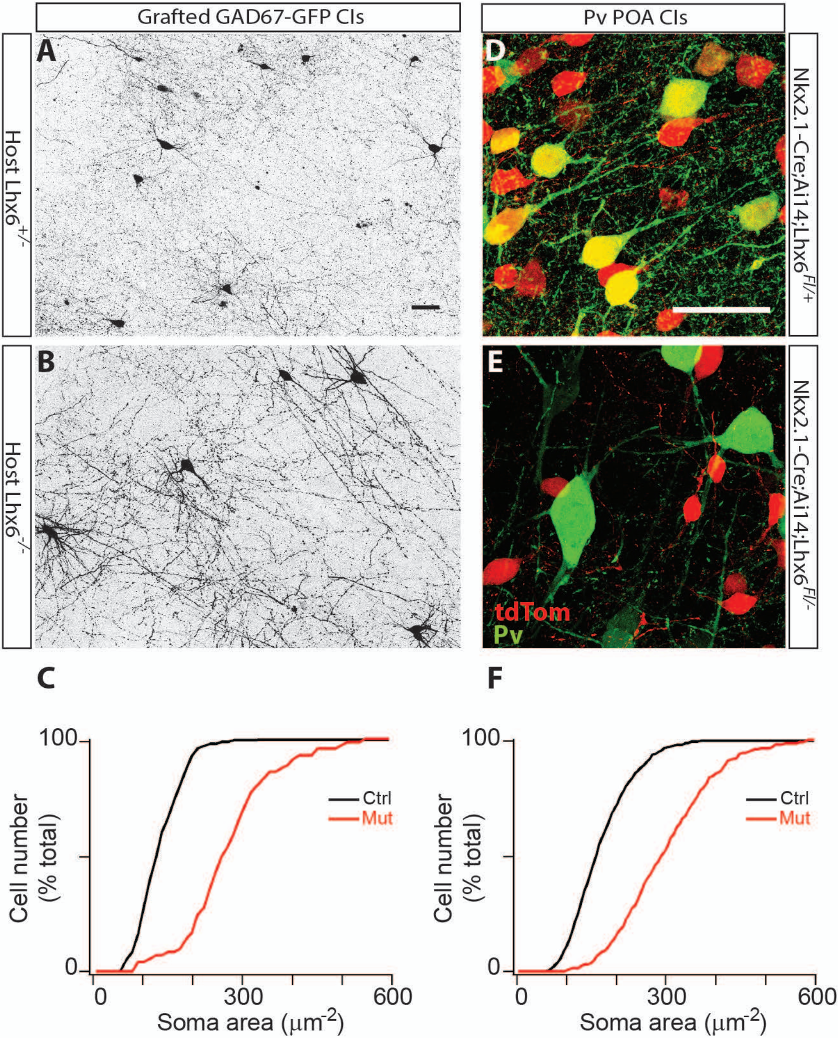

Grafted GAD67-GFP CIs
Pv POA CIs
A
B
C
D
E
F
Host Lhx6+/-
Host Lhx6-/-
Nkx2.1-Cre;Ai14;Lhx6Fl/+
Nkx2.1-Cre;Ai14;Lhx6Fl/-
tdTom
Pv
Cell number (% total)
Soma area (μm-2)
Ctrl
Mut
100
0
0
300
600

Figure S4: Large soma area observed in both grafted CIs and POA derived CIs in *Lhx6* mutant brains, Related to Figure 3. (**A-B**) Coronal sections from the somatosensory cortex of P16 wild type (**A**) and *Lhx6* mutant (**B**) mice grafted with wild type GAD67:GFP CI progenitors. Grafted CI morphology is revealed by GFP expression (converted to greyscale). (**D-E**) Coronal sections from the somatosensory cortex of P18 *Nkx2.1-Cre;Ai14;Lhx6*$^{fl+-}$ (**D**) and *Nkx2.1-Cre;Ai14;Lhx6*$^{fl/-}$ (**E**) immunostained for Pv (green). MGE derived CIs can be identified by tdT expression (red). Scale bars: 50 microns. (**C,F**) Quantification of soma size. Cumulative histograms shown in **C** and **F** correspond to the animals shown in **A/B** and **D/E**, respectively. Black: Ctrl; Red: Mut.

Figure S5

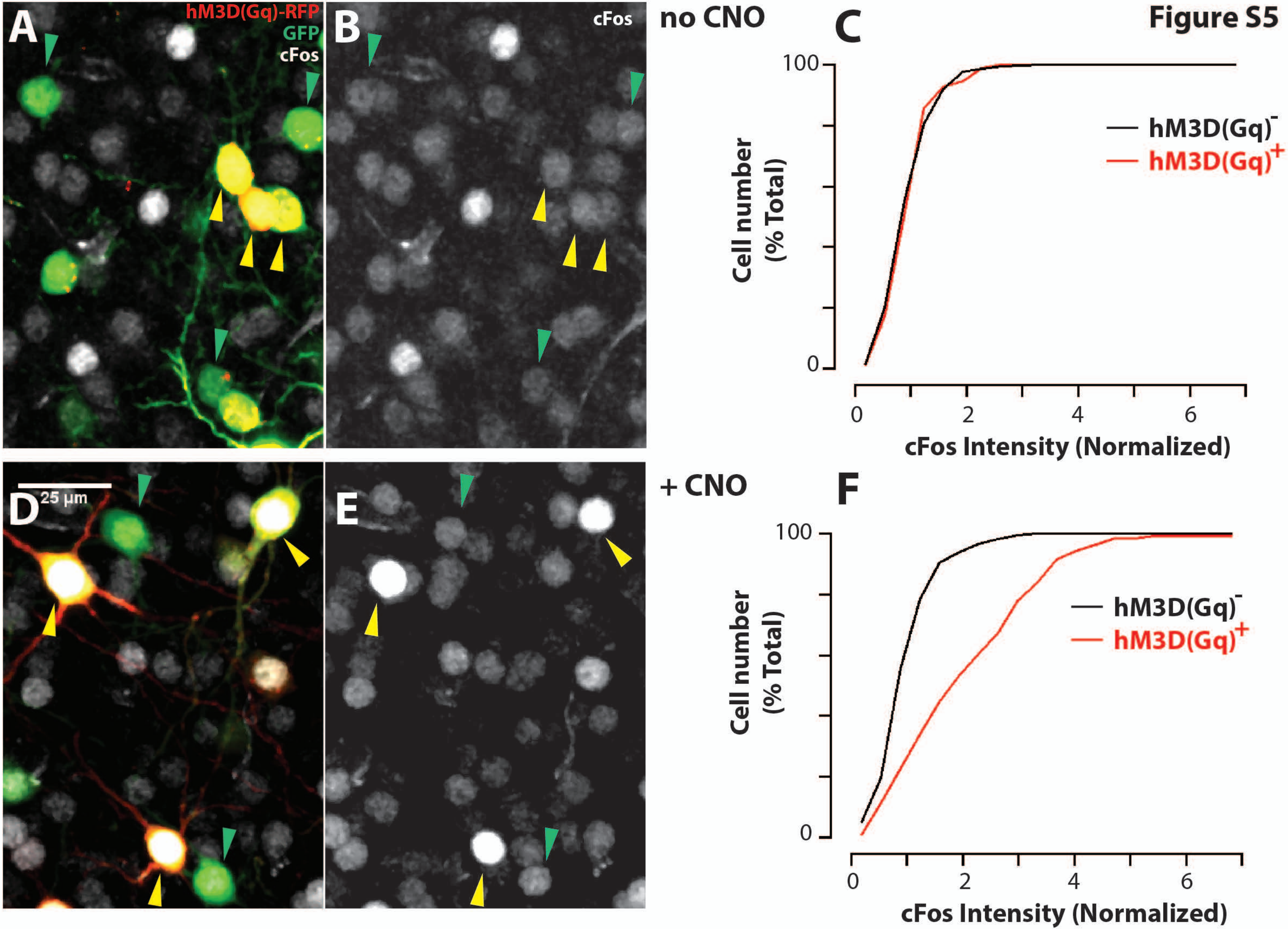

A
hM3D(Gq)-RFP
GFP
cFos
B
cFos
no CNO
C
100
0
Cell number (% Total)
hM3D(Gq)-
hM3D(Gq)+
0
2
4
6
cFos Intensity (Normalized)
D
25 μm
E
+ CNO
F
100
0
Cell number (% Total)
hM3D(Gq)-
hM3D(Gq)+
0
2
4
6
cFos Intensity (Normalized)

Figure S5: **Nuclear c-fos expression increases in hM3D(Gq) expressing CIs following CNO administration, Related to Figure 5**. (**A,B,D,E**) Confocal micrographs of coronal sections from the brain of mice transplanted at P0-P2 with CI precursors transfected with CAG:hM3D(Gq):IRES:RFP and CAG:IRES:GFP expression plasmids and immunostained for GFP (green), RFP (red) and c-fos (white). Sections are from vehicle only (**A**) or CNO (**D**) treated animals. Panels B and E are identical to those in A and D respectively, but show only the c-fos signal (white). (**C,F**) Cumulative histogram of normalized c-fos intensity in nuclei from cells that are either negative (black) or positive (red) for RFP (hM3D(Gq)). For CNO treated animals (**F**) the mean c-fos intensity of DREADD-Gq Positive nuclei was 2.17 ± 0.11 (n = 141 cells) while for DREADD-Gq Negative nuclei it was only 1.11 ± 0.04 (n = 209 cells, 3 mice ; significant by Wilcoxon rank test p<10-10). In contrast, for untreated animals (**C**) the mean cfos intensity of DREADD-Gq Positive nuclei was 1.1 ± 0.04 (n = 111 cells) and for DREADD-Gq Negative nuclei was 1.07 ± 0.02 (n = 338 cells, 3 mice ; not significant by Wilcoxon rank test p=0.5). Scale bars: 25 microns.

Table S1

Fate Mapping (Figures 1, 2, S1, S2)

**Cell Type**	**Average Control**	**SEM Control**	**Average Mutant**	**SEM Mutant**	**p**	**Test Used**
MGE CI SSC (cells/mm^2)	471	34	308	23	**0.02**	Student's t test
CGE CI SSC (cells/mm^2)	165	12	305	35	**0.01**	Student's t test
pan CI SSC (cells/mm^2)	650	31	631	9	0.6	Student's t test
MGE CI Layer I (% total)	5.3	0.6	20.4	1.3	**0.002**	Student's t test
MGE CI Layer II/III (% total)	18.3	3.5	10.1	1.9	0.12	Student's t test
MGE CI Layer IV (% total)	33	0.9	21	1.2	**0.001**	Student's t test
MGE CI Layer V (% total)	35.2	1.8	22.4	1.2	**0.006**	Student's t test
MGE CI Layer VI (% total)	13.9	2	28	0.9	**0.01**	Student's t test
CGE CI Layer I (% total)	14.4	1.8	13.4	2.1	0.7	Student's t test
CGE CI Layer II/III (% total)	60.2	3.6	35.5	2.9	**0.007**	Student's t test
CGE CI Layer IV (% total)	11.9	0.4	11.8	0.3	0.7	Student's t test
CGE CI Layer V (% total)	4.8	1	9.7	1.2	**0.03**	Student's t test
CGE CI Layer VI (% total)	8.7	2.8	29.7	1.3	**0.007**	Student's t test
pan CI Layer I (% total)	7.2	1.1	14.2	0.9	**0.01**	Student's t test
pan CI Layer II/III (% total)	48.3	1.3	24.6	1.8	**0.0006**	Student's t test
pan CI Layer IV (% total)	17.9	1.5	12.6	0.8	0.05	Student's t test
pan CI Layer V (% total)	16.1	1	18.1	1.8	0.4	Student's t test
pan CI Layer VI (% total)	10.5	0.8	30.4	2.2	**0.006**	Student's t test
$Reelin^+$ CGE^+ SSC (cells/mm^2)	49.9	5.7	84.3	7	**0.02**	Student's t test
VIP^+ CGE^+ SSC (cells/mm^2)	43	5	41.2	2.7	0.7	Student's t test

Pv^+ MGE^- SSC (cells/mm^2)	29.7	0.4	69.2	3.8	**0.009**	Student's t test
Sst^+ MGE^- SSC (cells/mm^2)	28	2.5	24.3	2.6	0.4	Student's t test
P2 MGE CI (cells/mm^2)	742	3	745	29	0.9	Student's t test
P2 CGE CI (cells/mm^2)	200	4.8	177.3	14	0.2	Student's t test
P7 MGE CI (cells/mm^2)	390	21	278	15	**0.01**	Student's t test
P7 CGE CI (cells/mm^2)	113	10	174	3.8	**0.02**	Student's t test
P2 $TUNEL^+MGE^+$ (% MGE^+)	0.22	0.04	0.93	0.09	0.07	Student's t test
P2 $TUNEL^+Sp8^+$ (% $Sp8^+$)	0.15	0.08	0.26	0.02	0.07	Student's t test
P7 $TUNEL^+MGE^+$ (% MGE^+)	3.1	0.4	7.6	1.3	**0.01**	Student's t test
P7 $TUNEL^+CGE^+$ (% CGE^+)	2	0.1	0.7	0.1	**0.0006**	Student's t test
MGE CI MC (cells/mm^2)	436	49	285	22	0.07	Student's t test
CGE CI MC (cells/mm^2)	138	9	237	15	**0.002**	Student's t test
$Reelin^+$ CGE^+ MC (cells/mm^2)	44.7	3.5	94.9	7.1	**0.009**	Student's t test
VIP^+ CGE^+ MC (cells/mm^2)	52.8	5.3	59.4	2.8	0.5	Student's t test
Pv^+ MGE^- MC (cells/mm^2)	36	4.1	82.8	5.3	**0.003**	Student's t test
Sst^+ MGE^- MC (cells/mm^2)	32.6	8.1	29.1	3	0.7	Student's t test
$Lhx6^+MGE^+$ (% MGE^+)	89.3	4.9	0.5	0.3	**0.003**	Student's t test
Pv^+MGE^+ (% MGE^+)	54.2	1.7	4.7	0.5	**0.001**	Student's t test
Sst^+MGE^+ (% MGE^+)	29	1.1	0.3	0.2	**0.001**	Student's t test
$Reelin^+MGE^+$ (% MGE^+)	22.9	1	8	1.9	**0.005**	Student's t test
VIP^+MGE^+ (% MGE^+)	0	0	5.1	0.4	**0.005**	Student's t test
$Sp8^+MGE^+$ (% MGE^+)	0	0	5.8	1	**0.03**	Student's t test

Grafting GAD67:GFP CIs (Figure 3)

Individual	Average Control Host	SEM Control Host	Mutant Host	N animals Control	n Cells Control	n Cells Mutant
Experiment 1 (% Control Average)	100	13	198	4	4933	2441
Experiment 2 (% Control Average)	100	9	184	5	16927	6223
Experiment 3 (% Control)	100		157	1	5434	8554
**Average**	**Average Mutant Host**	**SEM Mutant Host**	**p**	**Test Used**		
(% Control Average)	181	13	**0.02**	Student's t test single sample (mean 100)		

Grafting hM3D(Gq) expressing CI (Figure 5)

	Average Vehicle	SEM Vehicle	Average CNO	SEM CNO	p	test
RFP^+ (% GFP^+)	47	3	61	3	**0.01**	Student's t test (paired samples)
**Individual Experiments**	**Fraction Vehicle**	**Fraction CNO**	**N Cells Vehicle**	**N Cells CNO**		
Experiment 1 RFP^+ (% GFP^+)	53.63	65.76	330	444		
Experiment 2 RFP^+ (% GFP^+)	45.34	62.34	397	308		
Experiment 3 RFP^+ (% GFP^+)	41.67	54.86	228	175		

Soma Cell Size (Figure S4)

Cell Type	Average Control	SEM Control	Average Mutant	SEM Mutant	p	Test	n Cells Control	n Cells Mutant
Pv^+ MGE^- (μm^2)	171	2	300	7	**$<10^{-10}$**	Wilcoxon rank	701	199
	**Average Control Host**	**SEM Control Host**	**Average Mutant Host**	**SEM Mutant Host**	**p**	**Test**	**n Cells Control Host**	**n Cells Mutant Host**
Grafted CI (μm^2)	138	4	275	11	**$<10^{-10}$**	Wilcoxon rank	119	72

Table S1, Related to all Figures: Summary of statistical analysis.

Table S3:

Up-regulated genes	$2^{(-DDCT)}$	Down-regulated genes	$2^{(-DDCT)}$
Adam10	1.003	*Adcy1*	0.904
Adcy8	1.309	*Akt1*	0.809
Arc	1.257	*Camk2a*	0.768
Bdnf	**2.070**	*Camk2g*	0.889
Dlg4	1.053	*Cdh2*	0.750
Egr1	1.158	*Cebpb*	0.500
Egr2	**2.942**	*Cnr1*	0.865
Egr3	**1.983**	*Creb1*	0.766
Fos	1.172	*Crem*	**0.419**
Gabra5	1.447	*Egr4*	0.927
Grin1	1.086	*Ephb2*	0.919
Grin2a	1.104	*Gnai1*	0.783
Grin2d	1.024	*Gria1*	0.666
Grm4	1.341	*Gria2*	0.733
Igf1	**2.334**	*Gria3*	0.730
Kif17	1.437	*Gria4*	0.800
Mmp9	**1.522**	*Grin2b*	0.806
Nfkb1	1.243	*Grin2c*	0.556
Ngfr	**3.418**	*Grip1*	0.912
Nptx2	1.081	*Grm1*	0.597
Pick1	1.082	*Grm2*	**0.300**
Prkca	1.140	*Grm3*	0.546
Prkcg	1.189	*Grm5*	0.832
Prkg1	**5.387**	*Grm7*	0.759
Rab3a	1.176	*Grm8*	0.947
Rgs2	1.029	*Homer1*	0.740
Rheb	1.353	*Inhba*	0.701
Synpo	**2.349**	*Jun*	0.634
		Junb	0.845

		Klf10	0.685
		Mapk1	0.837
		Ncam1	0.758
		Ngf	0.694
		Nos1	0.968
		Nr4a1	0.626
		Ntf3	**0.221**
		Ntrk2	0.699
		Pcdh8	0.969
		Pim1	**0.469**
		Plat	0.781
		Pclg1	0.749
		Ppp1ca	0.916
		Ppp1cc	0.792
		Ppp2ca	0.910
		Ppp3ca	0.796
		Rela	0.827
		Reln	0.807
		Sirt1	**0.461**
		Srf	0.872
		Timp1	0.519
		Ywhaq	0.949

Table S3: Expression changes of activity associated genes in *Htr3aCre;Ai14;Lhx6*$^{-/-}$ compared to *Htr3aCre;Ai14;Lhx6*$^{+/-}$ mice at P7, Related to Figure 4. List of genes analysed (see also Supplemental Experimental Procedures). Red highlighted genes: transcript up-regulation >1.5, green highlighted genes: transcript down-regulation <0.5, orange highlighted genes: up-regulation of transcripts has been previously observed by RNA *in situ* hybridization (Arc, Egr1; Figures 4 E-H), while in the case of *cfos* increased protein levels have been observed by immunohistochemistry (Figures 4 I-Q).
