# Supplementary material for: Modulation of Apoptosis Controls Inhibitory Interneuron Number in the Cortex
Source: Cell Rep. 2018 Feb 13;22(7):1710–21. doi: 10.1016/j.celrep.2018.01.064 (PMC6230259; doi:10.1016/j.celrep.2018.01.064)
Supplement: Document S2. Article plus Supplemental Information [file mmc3.pdf]

Cell Reports

Article

Modulation of Apoptosis Controls Inhibitory Interneuron Number in the Cortex

Graphical Abstract

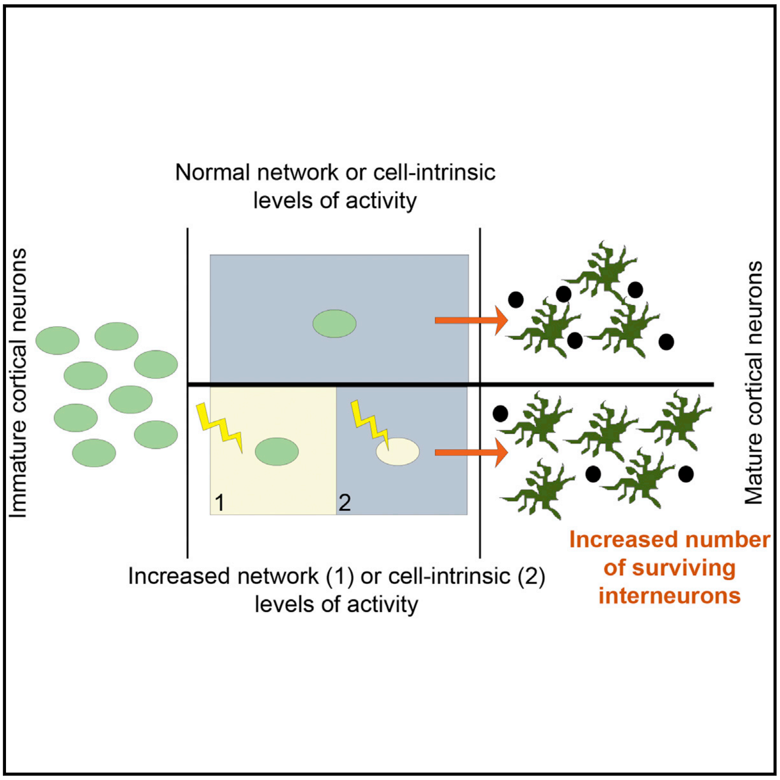

Highlights

- *Lhx6* is required for survival of CIs generated in the MGE
- MGE-derived CI loss is compensated for by a decrease in CGE-derived interneuron apoptosis
- Increases in cortical network activity are correlated with improved CI survival
- Transient, cell-autonomous depolarization improves the survival of grafted CIs

Authors

Myrto Denaxa, Guilherme Neves, Adam Rabinowitz, Sarah Kemlo, Petros Liodis, Juan Burrone, Vassilis Pachnis

Correspondence

denaxa@fleming.gr (M.D.), guilherme.neves@kcl.ac.uk (G.N.), juan.burrone@kcl.ac.uk (J.B.), vassilis.pachnis@crick.ac.uk (V.P.)

In Brief

Denaxa et al. address how the number of interneurons in the cortex is regulated. They show that apoptosis of developing interneurons can be modulated by activity in the forebrain of young mice so that increases in activity can rescue interneurons from apoptosis. This feedback loop provides a mechanism for fine-tuning the number and repertoire of interneurons in the brain.

Data and Software Availability

GSE108161

Denaxa et al., 2018, Cell Reports *22*, 1710–1721
February 13, 2018 © 2018
https://doi.org/10.1016/j.celrep.2018.01.064

OPEN ACCESS
CellPress

Cell Reports
Article

Modulation of Apoptosis Controls Inhibitory Interneuron Number in the Cortex

Myrto Denaxa,[1,6,7,*] Guilherme Neves,[2,3,7,*] Adam Rabinowitz,[4] Sarah Kemlo,[2,3] Petros Liodis,[5] Juan Burrone,[2,3,8,*] and Vassilis Pachnis[1,*]
[1]Nervous System Development and Homeostasis Laboratory, Francis Crick Institute, 1 Midland Road, London NW1 1AT, UK
[2]Centre for Developmental Neurobiology, Institute of Psychiatry, Psychology and Neuroscience, King's College London, London SE1 1UL, UK
[3]MRC Centre for Neurodevelopmental Disorders, King's College London, London SE1 1UL, UK
[4]Bioinformatics and Biostatistics Laboratory, Francis Crick Institute, 1 Midland Road, London NW1 1AT, UK
[5]Molecular Neurobiology, National Institute for Medical Research, the Ridgeway, Mill Hill, London NW7 1AA, UK
[6]Neuroscience Centre, Biomedical Sciences Research Centre "Alexander Fleming," Vari 16672, Greece
[7]These authors contributed equally
[8]Lead Contact
*Correspondence: denaxa@fleming.gr (M.D.), guilherme.neves@kcl.ac.uk (G.N.), juan.burrone@kcl.ac.uk (J.B.), vassilis.pachnis@crick.ac.uk (V.P.)
https://doi.org/10.1016/j.celrep.2018.01.064

SUMMARY

Cortical networks are composed of excitatory projection neurons and inhibitory interneurons. Finding the right balance between the two is important for controlling overall cortical excitation and network dynamics. However, it is unclear how the correct number of cortical interneurons (CIs) is established in the mammalian forebrain. CIs are generated in excess from basal forebrain progenitors, and their final numbers are adjusted via an intrinsically determined program of apoptosis that takes place during an early postnatal window. Here, we provide evidence that the extent of CI apoptosis during this critical period is plastic and cell-type specific and can be reduced in a cell-autonomous manner by acute increases in neuronal activity. We propose that the physiological state of the emerging neural network controls the activity levels of local CIs to modulate their numbers in a homeostatic manner.

INTRODUCTION

The balance between excitation and inhibition (E-I balance) is essential for the generation of optimal neural circuit activity and brain function. Cortical interneurons (CIs) represent the main source of γ-amino butyric acid (GABA)-mediated inhibition for excitatory projection neurons (PNs) in the pallium, and changes in the number or activity of CIs have been associated with neurodevelopmental and neuropsychiatric disorders such as epilepsy, schizophrenia, and autism spectrum disorders (Marín, 2012; Rubenstein and Merzenich, 2003). In contrast to cortical PNs, which are generated in the germinal zones of the dorsal telencephalon, CIs originate from progenitors in the subpallium (medial ganglionic eminence [MGE], caudal ganglionic eminence [CGE], and preoptic area [POA]) and, following stereotypic migration routes, reach the dorsal telencephalon, where they integrate into local circuits (Bartolini et al., 2013; Marín and Rubenstein, 2001; Wonders and Anderson, 2006). The disparate origin of PNs and CIs raises questions regarding the mechanisms that co-ordinate the size of these functionally interdependent neuronal populations of the cortex. A recent report has shown that CIs are generated in excess from basal forebrain progenitors and that BAX-dependent developmental cell death occurring over a critical postnatal period adjusts the final number of inhibitory neurons (Southwell et al., 2012). However, it is unclear whether postnatal apoptosis of CIs is controlled by an invariable cell-intrinsic program or can be modulated by the cellular composition and physiological state of the postnatal brain. We sought to address this question using genetic fate mapping to assess the survival of different CI populations in mutant mice characterized by reproducible changes in the cortical microenvironment.

Systematic gene expression analysis, genetic cell lineage tracing, and phenotypic characterization of mouse mutants have demonstrated that CI subtypes are specified by region-specific transcriptional programs operating within progenitor domains of the subpallium (Fishell and Rudy, 2011). *Lhx6* encodes a LIM homeodomain transcription factor that is specifically expressed by MGE-derived precursors and their derivative CIs expressing somatostatin (Sst) and parvalbumin (Pv). Consistent with its expression pattern, *Lhx6* mutations are characterized by a severe reduction in the number of Sst^+ and Pv^+ CIs but a normal complement of GABA-producing cells (Liodis et al., 2007; Zhao et al., 2008). These cellular phenotypes are associated with reduced inhibitory synaptic input on PNs, brain hyperactivity, and epilepsy-like phenotypes in postnatal animals (Neves et al., 2013). Here, we have combined phenotypic analysis of null and cell lineage-specific mutants, genetic lineage tracing, cell transplantation, and chemogenetic activation to query the specific responses of CI sub-lineages following the deletion of *Lhx6* activity. We find that *Lhx6* is required to maintain the normal complement of MGE-derived CIs and that reduction of this subpopulation in *Lhx6* mutants results in a surprising increase in the number of *Lhx6*-independent CGE-derived CIs and re-balancing of CI networks. The compensatory increase

 © 2018
This is an open access article under the CC BY-NC-ND license (http://creativecommons.org/licenses/by-nc-nd/4.0/).

of CGE-derived CIs is due to a reduction in apoptosis that can be modulated cell-autonomously by neuronal excitability during a critical postnatal period. Our results provide fundamental insight into the mechanisms that match the size of CI populations to the physiological requirements of cortical circuits and pave the way for better understanding the effect of neuronal activity on cell transplantation-based therapies.

RESULTS

Loss of MGE CIs Results in a Compensatory Increase in the Number of CGE CIs

Using general (*Gad1*) and subtype-specific (Pv and Sst) markers for cortical inhibitory neurons, we and others have reported that mice homozygous for null mutations of *Lhx6* have a reduced number of MGE-derived Sst^+ and Pv^+ CI subpopulations but that the total number of GABAergic neurons in the neocortex and hippocampus remains unchanged (Liodis et al., 2007; Zhao et al., 2008). We reasoned that this could result from two different scenarios: either *Lhx6* activity is necessary for the expression of late differentiation MGE markers, or *Lhx6* is required for the survival of MGE-derived CIs, and a compensatory increase of non-MGE-derived CIs in mutants maintains the total number of CIs. To unequivocally distinguish between these two possibilities, we combined gene targeting with genetic fate mapping. Generation of a novel conditional allele of *Lhx6* (*Lhx6^{fl}*) in the mouse (Supplemental Experimental Procedures; Figures S1A and S1B) and introduction into the *Lhx6$^{fl/-}$* genetic background of the Cre-dependent fluorescent reporter *Rosa26-tdTomato* (tdT, Ai14; Madisen et al., 2010) allowed us to use Cre drivers for cell-type-specific *Lhx6* ablation and simultaneous fate mapping of the mutant lineages. To validate the novel *Lhx6^{fl}* allele, we first used *VgatCre* (Vong et al., 2011) to delete *Lhx6* from all CI precursors. Consistent with the phenotype of *Lhx6*-null mutants, the population of Pv^+ and Sst^+ CIs was dramatically reduced in post-natal day 18 (P18) *VgatCre;Ai14;Lhx6$^{fl/-}$* mice relative to *VgatCre;Ai14;Lhx6$^{fl/+}$* controls (Figures S1C–S1F), whereas the total number of tdT^+ cells remained unchanged (Figures 1A, 1B, and 1G). Next we used the *Nkx2.1Cre* transgenic driver (Kessaris et al., 2006) to specifically track Lhx6-deficient MGE-derived CIs. As expected, the percentage of tdT^+ CIs co-labeled with antibodies against Lhx6, Pv, Sst, and Reelin was dramatically reduced in *Nkx2.1Cre;Ai14;Lhx6$^{fl/-}$* mice relative to *Nkx2.1Cre;Ai14;Lhx6$^{fl/+}$* controls (Figures S1G–S1L). Furthermore, we observed that the cortex of *Nkx2.1Cre;Ai14;Lhx6$^{fl/-}$* mice contained a small number of tdT^+ cells co-expressing VIP or Sp8, markers that normally are associated with CGE-derived CIs and are absent from their MGE-derived counterparts (Figures S1M and S1N; Vogt et al., 2014). Notably, the overall number of tdT-expressing cells in the cortex of P18 *Nkx2.1Cre;Ai14;Lhx6$^{fl/-}$* mice was significantly smaller relative to controls (Figures 1H and S2A), suggesting that, in addition to its well-established role in CI subtype specification, *Lhx6* is also required for the survival of MGE-derived CIs.

The reduced number of tdT^+ MGE-derived CIs observed in *Nkx2.1Cre;Ai14;Lhx6$^{fl/-}$* mice (Figures 1C, 1D, and 1H and S2A) in conjunction with the nearly normal number of GABAergic interneurons in the cortex of either *Lhx6*-null mice (Liodis et al., 2007; Zhao et al., 2008) or mice with pan-CI deletion of *Lhx6* (*VgatCre;Ai14;Lhx6$^{fl/-}$*; Figure 1G), suggests that non-MGE-derived CI lineages compensate for the specific loss of MGE-derived CIs. To examine this possibility we fate-mapped CGE-derived CIs in *Lhx6$^{-/-}$* mice using the CGE-specific Cre driver *Htr3aCre* (http://www.gensat.org; Supplemental Experimental Procedures) and the Ai14 (tdT) reporter. We observed increased representation of CGE-derived tdT^+ cells in the cortex of *Lhx6* mutant mice (*Htr3aCre;Ai14;Lhx6$^{-/-}$*) relative to controls (*Htr3aCre;Ai14;Lhx6$^{+/-}$*) (Figures 1E, 1F, and 1I and S2D). Therefore, the size of the CGE-derived CI population in the mammalian cortex is not pre-determined but can be modulated to compensate for the loss of MGE-derived interneurons in the cortex. Although the total number of CIs is not affected by the *Lhx6* deletion, the radial distribution of CIs is severely disrupted in *Lhx6* mutants, with CIs congregating along the upper and deeper layers of the cortex (Figure 1J; Liodis et al., 2007; Zhao et al., 2008). Specifically, MGE-derived CIs in *Lhx6* mutants are lost in the middle layer (IV), whereas the most obvious increase in CGE-derived CIs occurs in deeper layers (V and VI) (Figures 1K and 1L). This observation argues against a local survival signal that compensates for CI loss within cortical layers but, rather, suggests that CI number is modulated by a more global signal that operates across the cortex.

The majority of CGE-derived CIs can be accounted for by two functionally and molecularly distinct sub-populations marked by the expression of either VIP or Reelin (Fishell and Rudy, 2011; Kepecs and Fishell, 2014; Lee et al., 2010). Immunostaining of *Lhx6* mutant brain sections from CGE-labeled CI mice (*Htr3aCre;Ai14;Lhx6$^{-/-}$*) using these subtype markers showed that only the $Reelin^+$ subset increased in number, whereas the VIP^+ subpopulation remained unchanged (Figures 1M and 1N and S2E and S2F). Interestingly, ablation of *Lhx6* from MGE lineages also resulted in altered representation of CI subtypes originating outside of the ganglionic eminences (Gelman et al., 2011). Thus, in *Nkx2.1Cre;Ai14;Lhx6$^{fl/-}$* mice, the number of tdT^-Pv^+ interneurons (which partly originate from the POA; Figures S2G–S2N) increased relative to controls (*Nkx2.1Cre;Ai14;Lhx6$^{fl/+}$*), whereas the number of tdT^-Sst^+ CIs remained unchanged (Figures 1O and 1P and S2B and S2C). Based on a previous characterization of the Nkx2.1Cre line used here, some MGE-derived CIs fail to express Cre recombinase (mostly a subpopulation expressing Sst (Fogarty et al., 2007)) and can, therefore, represent MGE-derived cells that still express Lhx6 (Figures 1O and 1P and S2B and S2C). However, we found no differences in the number of GFP^-Lhx6^+ cells in control and mutant brains (Figure S1O), suggesting that the size of this ‘‘escapee’’ population does not increase in mutant brains and, therefore, cannot be responsible for the increase in tdT^-Pv^+ cells in mutants. These findings suggest that the compensatory responses of CIs to *Lhx6* ablation are subtype-specific and occur across different lineages.

Reciprocal Changes in Apoptosis of CGE and MGE CIs in *Lhx6* Mutants

The increased representation of CIs originating outside of the MGE in the cortex of *Lhx6*-deficient mice could result from enhanced proliferation of their progenitors or reduced neuronal

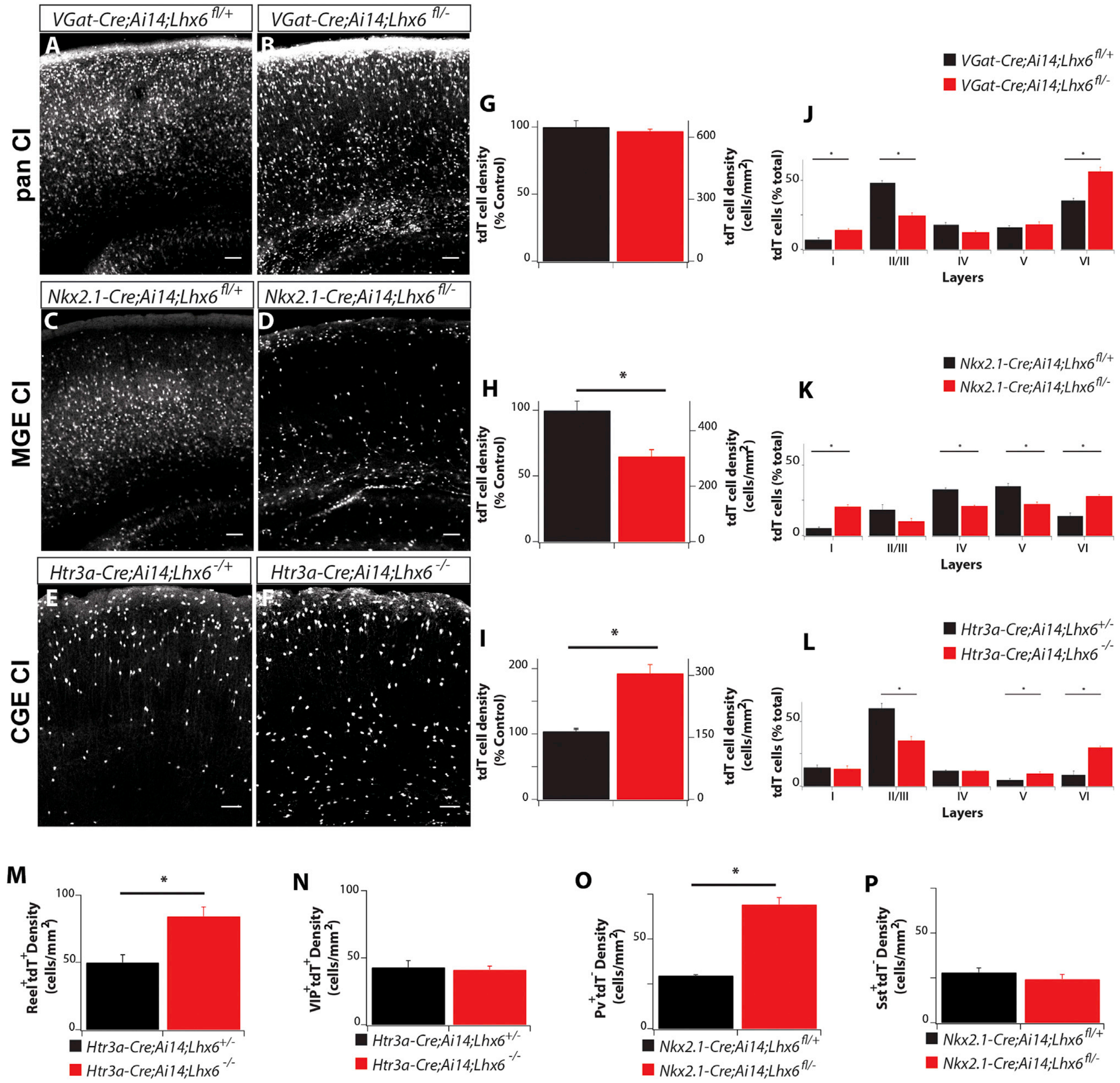

Figure 1. Fate Mapping Reveals Reduced Viability of *Lhx6*-Deficient MGE CIs and Increased Survival of CGE CIs

(A–F) tdT^+ (white signal) CIs in sections from the somatosensory cortex of *Lhx6* control (Ctrl; A, C, and E) and mutant (Mut; B, D, and F) P18 mice fate-mapped using different Cre driver lines, as indicated. Shown are the entire CI population (A and B), MGE-derived (C and D), and CGE-derived (E and F).

(G–I) Quantification (raw densities, right axis; normalized to the average control level, left axis) of tdT^+ cell density in somatosensory cortices for all CIs (G), MGE (H), and CGE (I) derived CIs.

(J–L) Quantification of the distribution of tdT^+ cells across cortical layers for all CIs (J), MGE (K), and CGE (L) derived CIs.

(M–P) Quantification of cell densities of CGE-derived $Reelin^+$ (M), CGE-derived VIP^+ (N), non-MGE-derived Pv^+ (O), and non-MGE-derived Sst^+ cells (P).

See also Figure S1 for characterization of the $Lhx6^{fl}$ allele. Similar results were observed in the motor cortex (Figure S2). See Figure S3 for characterization of non-MGE derived Pv^+ cells in *Lhx6* mutants. In all figures, data are expressed as mean ± SEM. Statistical significance was evaluated using Student's t test, unless otherwise stated. $^*p < 0.05$. Scale bars are 100 μm.

cell death during the critical postnatal window of apoptosis (Southwell et al., 2012; Yamaguchi and Miura, 2015). To distinguish between these possibilities, we first compared the number of proliferating progenitors (identified by pH3 immunostaining and 5-ethynyl-2'-deoxyuridine [EdU] uptake) within the ganglionic eminences of *Lhx6* mutant and control embryonic day

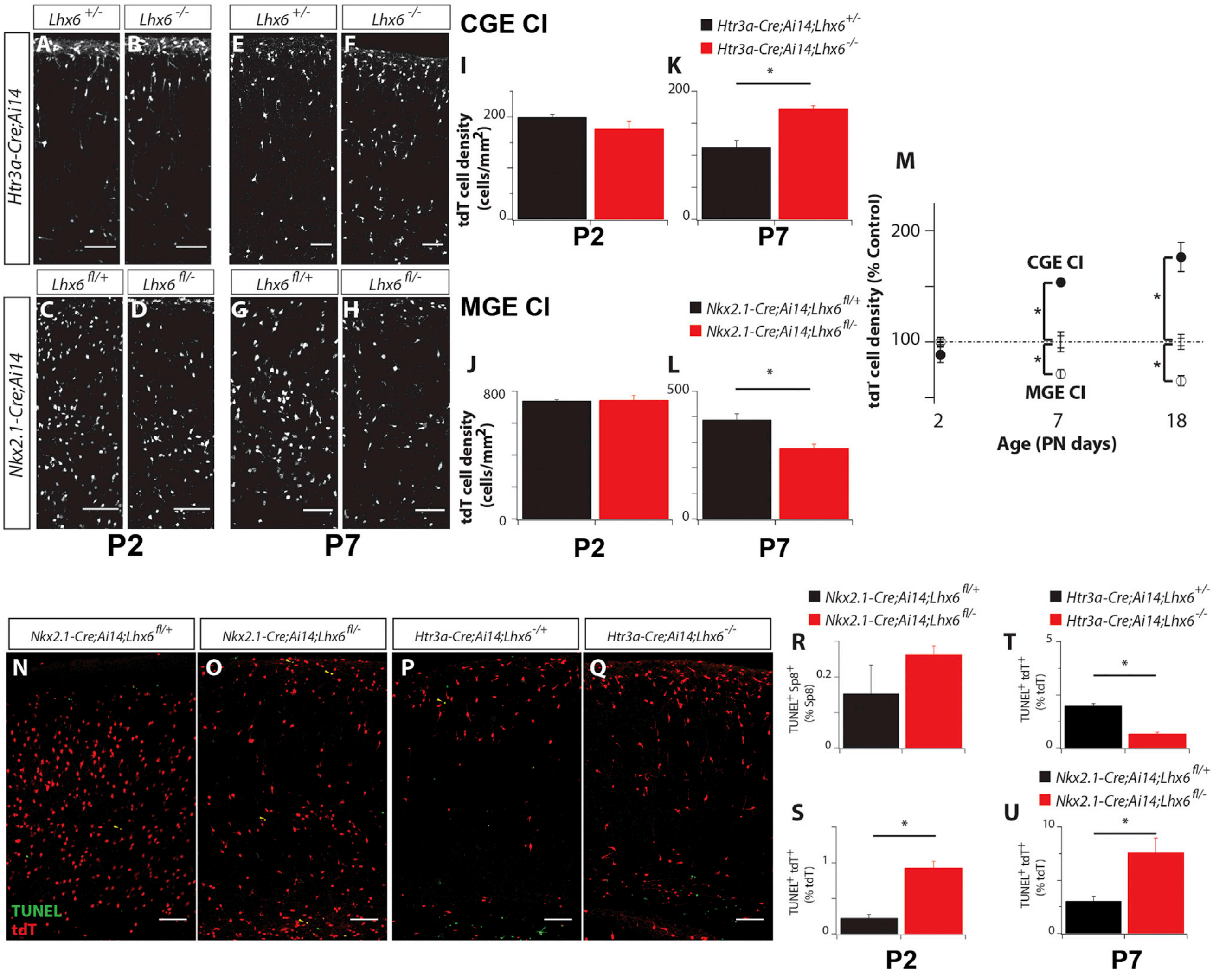

Figure 2. Reduced Apoptosis of CGE CIs Compensates for the Loss of *Lhx6*-Deficient MGE Counterparts

Shown are changes in the number of CI subtypes in *Lhx6* control and mutant mice during early post-natal development.

(A–H) tdT-expressing CIs in cortical sections from P2 (A–D) and P7 (E–H) *Lhx6* control (A, C, E, and G) and mutant (B, D, F, and H) mice. tdT expression identifies CGE-derived (A, B, E, and F) and MGE-derived CIs (C, D, G, and H), respectively.

(I–L) Quantification of the distribution of tdT^+ cell density in the cortex of P2 (I and J) and P7 (K and L) for CGE (I and K) and MGE (J and L) derived CIs.

(M) Summary of changes in density of CGE (closed circles) and MGE (open circles) CIs in *Lhx6* mutant cortices relative to *Lhx6* controls (dotted line) at P2, P7, and P18.

(N–Q) Representative cortical sections from P7 somatosensory cortices showing fate-mapped CIs (tdT^+ in red) and $TUNEL^+$ (green) cells. Yellow arrowheads indicate $TUNEL^+$ fate mapped CIs. In (N) and (O), tdT expression represents MGE-derived CIs, whereas in (P) and (Q), tdT represents CGE-derived CIs.

(R and S) Quantification of $TUNEL^+$ cells for CGE-derived CIs (R; $Sp8^+TUNEL^+$) and MGE-derived CIs (S; tdT^+TUNEL^+) at P2 for control and *Lhx6* mutant cortices.

(T and U) Quantification of $TUNEL^+$ cells for CGE-derived (T) and MGE-derived (U) CIs undergoing apoptosis (tdT^+TUNEL^+) at P7 for control and *Lhx6* and mutant cortices.

See Figure S4 for characterization of proliferation and migration of CI precursors in embryonic *Lhx6* mutant brains. Scale bars are 100 μm.

14.5 (E14.5)–E16.5 embryos. The number of $pH3^+$ and EdU^+ progenitors was similar between the two genotypes, suggesting that the mechanism(s) responsible for the increased representation of CGE-derived CIs in *Lhx6* mutants operates on post-mitotic interneuron precursors at later developmental stages (Figures S3A–S3J). In agreement with this hypothesis, there was no difference in the number of cells expressing Sp8 (a transcription factor expressed by non-MGE-derived CIs; Ma et al., 2012) in E16 control and mutant cortices (Figures S3K–S3N). However, we note that a small proportion of Sp8-expressing cells in mutant cortices is derived from the MGE (Figure S1N). In further support of this idea, the number of fate-mapped MGE- and CGE-derived CIs at P2 (a developmental stage that follows the completion of CI tangential migration but precedes the onset of apoptosis; Miyoshi and Fishell, 2011; Southwell et al., 2012) was similar between control and *Lhx6* mutant mice (Figures 2A–2D, 2I, and 2J).

OPEN
ACCESS
CellPress

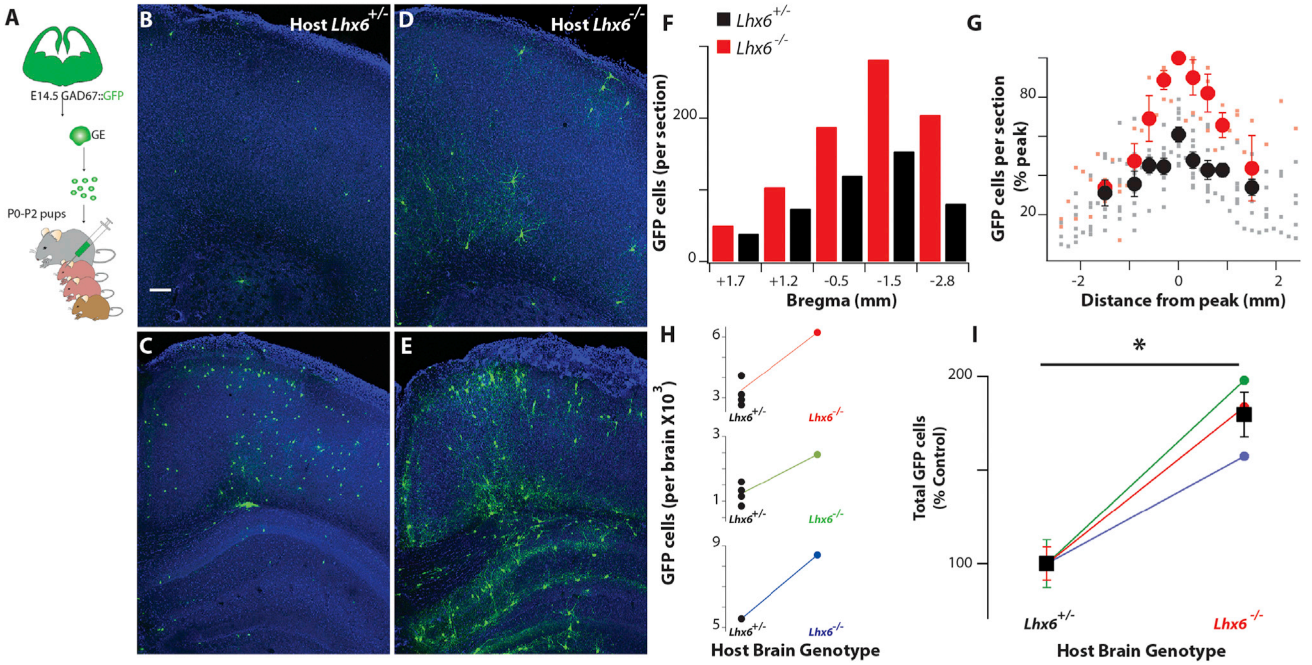

Figure 3. Cellular Microenvironment of *Lhx6* Mutant Forebrain Promotes Grafted CI Progenitor Survival

(A) Schematic representation of the CI progenitor transplantation into the cortex of P0–P2 pups.

(B–E) Coronal brain sections of Lhx6$^{+/-}$ (B and C) and Lhx6$^{-/-}$ (D and E) P16 mice transplanted at P0–P2 with wild-type GFP$^+$ CI progenitors. The sections shown in (B) and (D) are more anterior to those shown in (C) and (E).

(F) Distribution of surviving GFP$^+$ CIs in the cortex of an *Lhx6*$^{+/-}$ (black bars) and an *Lhx6*$^{-/-}$ (red bars) littermate pair of P16 mice grafted with CI precursors at P0–P2 at different rostro-caudal levels.

(G) Distribution of GFP$^+$ cells was similar in all cortices analyzed, with a peak in the presumed injection site. Regardless of the rostro-caudal level quantified, more GFP$^+$ cells were present in *Lhx6* mutant host cortices (red) than in their control littermates (black).

(H and I) Quantification of surviving GFP$^+$ CIs in the entire cortex of *Lhx6*$^{+/-}$ and *Lhx6*$^{-/-}$ P16 mice. In 3 independent experiments (H), the number of surviving GFP$^+$ cells in the *Lhx6* mutant host cortices was higher than that observed in control littermates grafted with the same cell suspension. (I) Values (taken from H) for the mutants (*Lhx6*$^{-/-}$, circles) are normalized to the average value in control littermates (*Lhx6*$^{+/-}$; colored bars represent SEM for individual grafting experiments that correspond to those in H). The number of GFP$^+$ cells found in *Lhx6*$^{-/-}$ was 181% ± 13% higher in comparison with control littermates (p = 0.02, one sample t test, mean 100, n = 3 *Lhx6*$^{-/-}$ and 10 *Lhx6*$^{+/-}$, minimum of 500 cells counted per brain).

See Figure S5 for morphological characterization of wild-type GFP$^+$ CI progenitors grafted in *Lhx6* mutant brains. Scale bars are 100 μm.

In contrast, 5 days later (at P7), we observed a decreased representation of MGE-derived CIs and a reciprocal increase of their CGE-derived counterparts (Figures 2E–2H, 2K, and 2L). The changes in the size of MGE- and CGE-derived CIs populations observed at P7 foreshadow those observed at P18 (Figures 1H and 1I and 2M) and, together, suggest lineage-specific modulation of the apoptotic programs of CIs by the *Lhx6* mutation.

To confirm this, we used terminal deoxynucleotidyl transferase dUTP nick end labeling (TUNEL) staining to quantify the extent of developmental cell death of MGE- and CGE-derived CIs at P2, to obtain an early readout of apoptosis in *Lhx6* mutants, and at P7, when expression of apoptotic markers in CIs is at its highest (Figure 2J; Southwell et al., 2012). TUNEL analysis at P2 revealed a marked increase in apoptosis of MGE-derived CIs (but not CGE-derived CIs) in *Lhx6*-null mutants even before the expected developmental apoptosis program begins (Figures 2R and 2S). A similar analysis in the cortex of *Nkx2.1Cre;Ai14;Lhx6*$^{fl/-}$ mice at P7 also showed a significant increase in the number of tdT$^+$TUNEL$^+$ cells relative to control animals (*Nkx2.1Cre;Ai14;Lhx6*$^{fl/+}$; Figure 2U). However, although CGE-derived CIs do not express *Lhx6*, the number of tdT$^+$TUNEL$^+$ double-positive CIs in the cortex of P7 *Htr3aCre;Ai14;Lhx6*$^{-/-}$ mice was significantly reduced relative to control littermates (*Htr3aCre;Ai14;Lhx6*$^{+/-}$; Figure 2U). We suggest that ablation of *Lhx6* during embryogenesis reduces the viability of MGE-derived CIs from an early stage in development. This reduction in the number of MGE-derived CIs triggers a compensatory and non-cell-autonomous decrease in the rate of developmental cell death in CGE-derived CIs during the normal period of CI apoptosis.

Enhanced Survival of Wild-Type CIs Grafted into the *Lhx6* Mutant Cortex

To explore the possibility that the cortical microenvironment of *Lhx6* mutants can rescue CIs from apoptosis, we transplanted GFP$^+$ CI precursors isolated from the basal forebrain of GAD-GFP E14.5 embryos (Tamamaki et al., 2003) into the pallium of neonatal (P0–P1) *Lhx6*$^{-/-}$ pups and their control (*Lhx6*$^{+/-}$) littermates (Figure 3A). Multiple morphologically mature GFP$^+$ CIs were observed throughout the cortex at P16 in both control

and mutant animals (Figures 3B–3F). No differences were observed in the spatial distribution of grafted CIs in the host cortices (Figures 3F and 3G). However, a subset of grafted CIs in mutant cortices showed striking morphologies, with consistently larger somata and dendritic arbors, a finding that mirrored the unusually large size of endogenous POA-derived Pv^+ CIs observed in *Lhx6* mutant mice (Figure S4).

The majority of grafted CI precursors are eliminated by BAX-dependent apoptosis within 2 weeks of transplantation (Southwell et al., 2012), a feature that recapitulates the timeline of programmed cell death of endogenous CIs. Consistent with this idea, we found that the number of transplanted GFP^+ CIs in the cortex of P16 *Lhx6*$^{-/-}$ mice was higher relative to that in *Lhx6*$^{+/-}$ littermate controls. These results were consistent across three independent experiments, regardless of the number of grafted interneurons (181% ± 13% of control, $p = 0.02$, $n = 3$ litters; Figures 3H and 3I), confirming the notion that the microenvironment of the host brain can modulate the survival of interneurons in the cortex.

Transcriptomic Analysis of Lhx6-Deficient Brains/ Lineages

To provide insight into the mechanisms by which the cortical microenvironment of *Lhx6* mutants controls the survival of grafted CI progenitors, we used RNA sequencing (RNA-seq) to compare the global transcriptome of the forebrain dissected from control (*Lhx6*$^{+/-}$) and mutant (*Lhx6*$^{-/-}$) mice at P15, a stage at which apoptosis of grafted CIs is at its highest. Differential gene expression analysis identified 1,707 genes that were significantly upregulated (977) or downregulated (730) (Figure 4A) in mutant relative to control littermates (summary results are presented in Table S2). In addition to *Lhx6* (which was absent from mutant samples), *Sst* and *Pv* were among the top downregulated genes in *Lhx6* mutants, in agreement with our immunocytochemistry data (Figures S1J and S1K). We also detected increased expression of genes normally expressed in CGE CIs in mutant cortices, such as Vip and Npy. Interestingly, inspection of the list of upregulated genes identified several—including *Bdnf* (Hartmann et al., 2001), *Npas4* (Bloodgood et al., 2013), *Fosb* (Eagle et al., 2015), and *Npy* (Gall et al., 1990)—whose expression is induced by neuronal activity. Gene set enrichment analysis using the hypergeometric test showed that the genes significantly modulated in *Lhx6*-deficient forebrains significantly overlap with genes altered in pyramidal neurons following chronic increases in activity (either through 48 hr inhibition of ionotropic GABA receptors in hippocampal neurons [$p < 10^{-59}$; Yu et al., 2015] or through activation of L-type voltage-dependent Ca^{2+} channels in cortical neurons [$p = 2.3 \times 10^{-3}$; Qiu et al., 2016]). In fact, hierarchical clustering based on the expression of the top 25 genes upregulated by chronic activity clearly distinguished between control and mutant samples (Figure 4B). Finally, the expression of the activity-dependent gene *cfos* (Cohen and Greenberg, 2008) was highly upregulated throughout the cortex of *Lhx6* mutants (Figures 4C and 4D). These transcriptomic results provide a molecular confirmation of increased network activity in the cortex, as would be expected for brains where the development of MGE-derived CIs is compromised (Batista-Brito et al., 2009; Neves et al., 2013).

Next we compared the expression of neuronal activity markers between control and *Lhx6*-deficient cortices at P7, a postnatal stage characterized by the highest rate of interneuron cell death (Southwell et al., 2012). This analysis showed a dramatic upregulation of a number of immediate-early genes, including the activity-regulated cytoskeleton-associated protein *Arc* (Tzingounis and Nicoll, 2006), the early growth response protein *Egr1* (French et al., 2001), and *cfos*, in the cortex of *Lhx6*-deficient mice relative to controls (Figures 4E–4J). Interestingly, overexpression of the activity-dependent markers was observed mostly in the deeper layers of the cortex (Figures 4K and 4L), which also showed the highest increase in the number of CGE-derived CIs (Figures 1E, 1F, and 1L). Our gene expression analysis demonstrates a correlation between increased immediate-early gene expression, which is reflective of enhanced network activity, and enhanced survival of CIs in the cortex of *Lhx6* mutant mice.

Next we compared the expression of immediate-early gene markers specifically in CGE-derived CIs labeled with tdT in *Htr3aCre;Ai14;Lhx6*$^{-/-}$ versus *Htr3aCre;Ai14;Lhx6*$^{+/-}$ mice at P7. First, *cfos* immunostaining showed that the number of $cFos^+tdT^+$ neurons (yellow arrowheads in Figure 4P) was increased in the *Lhx6*-deficient cortex relative to controls, suggesting increases in the activity of CGE CIs (Figures 4O–4Q). To further characterize such changes, we employed RT^2 profiler PCR array technology (Supplemental Experimental Procedures) to compare the expression of a panel of known activity-associated genes in CGE-derived CIs isolated by flow cytometry from the brain of P7 *Htr3aCre;Ai14;Lhx6*$^{-/-}$ and *Htr3aCre;Ai14;Lhx6*$^{+/-}$ mice (Table S3). Among the genes upregulated (>1.5-fold change) in CGE CIs from *Lhx6*-deficient brains were a number of genes associated with increased activity levels, including two members of the EGR family (*Egr2* and *Egr3;* DeSteno and Schmauss, 2008; Li et al., 2007), the neurotrophic factor *bdnf* (Hartmann et al., 2001), as well as genes implicated in growth factor signaling, such as insulin-like growth factor 1 (*Igf1*; Mardinly et al., 2016) and the nerve growth factor receptor (*Ngfr*; Meeker and Williams, 2015). These factors and their receptors play a crucial role in the control of neuronal numbers and dendritic growth. Together, these findings identify increased activity of CIs as a potential mechanism that drives their enhanced survival in hyperactive cortical networks.

Cell-Autonomous Increase in the Activity of CIs Enhances Survival

To directly test whether CI survival is regulated by neuronal activity in a cell-autonomous manner, we transplanted CI precursors expressing designer receptors exclusively activated by designer drugs (DREADDs) and modulated their activity by administering the appropriate ligands (Urban and Roth, 2015). Specifically, the MGE of E14.5 embryos was co-electroporated with a bi-cistronic expression vector encoding the hM3D(Gq) DREADD and red fluorescent protein (RFP) and a control plasmid encoding GFP. Transfected CIs were mechanically dissociated, and the resulting cell suspension was grafted in the cortex of P0–P1 wild-type mice. Because only a fraction of electroporated (GFP^+) neurons co-expressed hM3D(Gq) (RFP^+) (Figures 5A–5E),

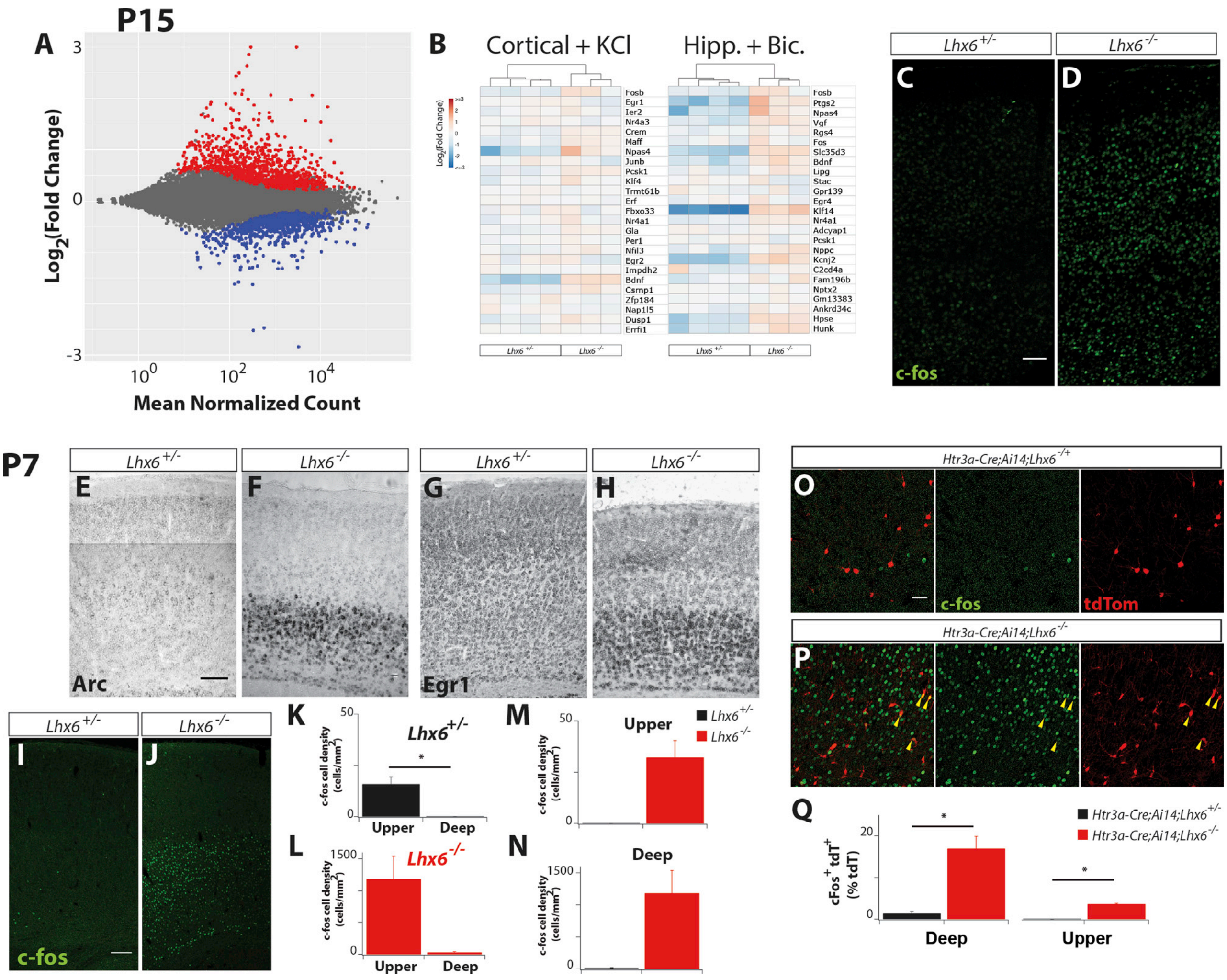

Figure 4. Molecular Analysis of *Lhx6* Mutant Forebrains Reveal Widespread Upregulation of Activity-Dependent Genes

(A) MA plot summarizing the results of the differential expression analysis between *Lhx6*$^{+/-}$ and *Lhx6*$^{-/-}$ P15 forebrains. Significantly upregulated genes are shown in red (977 genes), whereas significantly downregulated genes are shown in blue (730 genes). Significance was set as a false discovery rate of $\leq$ 0.05.

(B) Hierarchical clustering of *Lhx6*$^{+/-}$ and *Lhx6*$^{-/-}$ forebrain samples using activity-regulated genes. Clusters were generated using the expression levels of the 25 most significantly upregulated genes in either cortical cultures treated with KCL + FPL64176 (Qiu et al., 2016) or hippocampal cultures treated with bicuculline (Yu et al., 2015). Genes upregulated by either treatment are similarly upregulated upon deletion of *Lhx6*.

(C and D) Coronal sections from the somatosensory cortex of *Lhx6*$^{+/-}$ (C) and *Lhx6*$^{-/-}$ (D) P15 mice immunostained for cfos (green).

(E–H) *In situ* hybridization of somatosensory cortex sections from *Lhx6*$^{+/-}$ (E and G) and *Lhx6*$^{-/-}$ (F and H) P7 mice with either *Arc* (E and F) or *Egr1* (G and H) riboprobes shows immediately early gene upregulation in the bottom layers of mutant brains.

(I and J) Coronal sections of the somatosensory cortex of *Lhx6*$^{+/-}$ (I) and *Lhx6*$^{-/-}$ (J) P7 mice immunostained for cfos (green); note the upregulation of cfos expression, particularly in the lower half of the cortex.

(K–M) Quantification and distribution of cfos^{+} cell density in upper (M) and deep (N) areas of *Lhx6*$^{+/-}$ (K) and *Lhx6*$^{-/-}$ (L) cortices.

(O and P) High-magnification images of the bottom half of the somatosensory cortex of *Htr3a-Cre;Ai14;Lhx6*$^{+/-}$ (O) and *Htr3a-Cre;Ai14;Lhx6*$^{-/-}$ (P) P7 brains immunostained for cfos (green). Yellow arrowheads show examples of cfos-expressing CGE-derived tdT^{+} cells.

(Q) Quantification of the distribution of cFos+ tdT+ (CGE-derived CIs) in the cortex.

See Table S2 for a summary description of mRNA sequencing results for the genes highlighted in (A) and Table S3 for a summary of results of the RT^2 profiler PCR array for CGE-derived CIs isolated by fluorescence-activated cell sorting (FACS) from P7 cortices. Scale bars are 100 μm for (C)–(J) and 50 μm for (O) and (P).

the GFP^{+}RFP^{-} population served as an internal control for the effect of DREADD ligands. Indeed, administration of the DREADD ligand clozapine-N-oxide (CNO) selectively increased the activity of transfected GFP^{+}RFP^{+} cells (Figure S5). Importantly, CNO treatment (administered twice daily from P14–P17) resulted in an increase in the fraction of GFP^{+}RFP^{+} (yellow arrowheads) relative to GFP^{+}RFP^{-} (white arrows) cells compared with vehicle-administered littermates (Figures 5F–5J), suggesting that enhanced activity is sufficient to protect CIs from programmed cell death in an otherwise normal brain. Our data provide evidence that neuronal activity modulates the number of CIs in the cortex in a cell-autonomous manner.

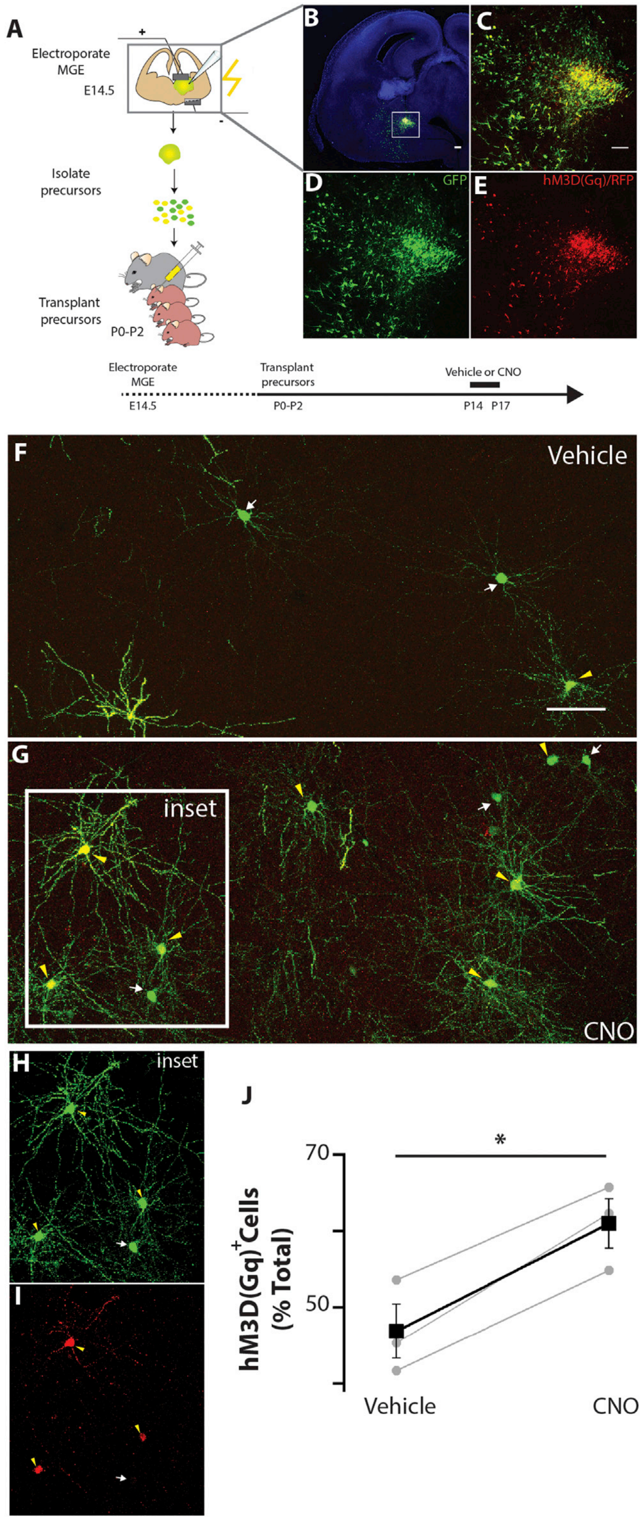

Figure 5. Cell-Autonomous Depolarization of CIs Enhances Their Survival

(A) Schematic representation of the brain acute slice electroporation, grafting, and vehicle/CNO administration protocol. Drug administration was targeted to coincide with the peak of apoptosis of transplanted CI progenitors.

(B–E) Coronal section from an E14.5 embryo brain transfected with the CAG:IRES:GFP (pGFP) and CAG:hM3D(Gq):IRES:RFP (pDREADRFP) plasmids and cultured for 12 hr (B). The boxed area is magnified to show the expression of both fluorescent reporters (C), GFP only (D), and RFP only (E).

(F–I) Representative sections from the somatosensory cortex of P17 mice grafted at P0–P2 with CI precursors transfected with the pGFP and pDREADDRFP plasmids and injected with either vehicle (F) or CNO (G). Yellow arrowheads identify cells expressing both plasmids, whereas green arrows indicate cells expressing GFP only (H and I). The boxed region in (G) is magnified to reveal the expression of GFP (H) and RFP (I).

(J) Quantification of RFP^+ cells found in the forebrain of P17 mice transplanted at P0–P2 (normalized to the total GFP^+ population). RFP^+ (vehicle] = 47% ± 3%, CNO = 61% ± 3%, p = 0.01, Student's paired samples t test, n = 3 vehicle and 3 CNO, minimum of 150 cells counted per brain.

See Figure S6 for analysis of the effects of CNO administration in hM3D(Gq)-expressing cells. Scale bars are 200 μm for (B) and 100 μm for (C)–(I).

DISCUSSION

Distinct physiological mechanisms, collectively referred to as homeostatic plasticity, operate in the nervous system to maintain or restore the balance between excitation and inhibition, even after considerable disruption of network dynamics (Turrigiano, 2012). For such "acute" mechanisms to be effective, it is essential that all physiologically relevant cellular compartments achieve a critical size and correct composition during development. How the output of developmental programs that specify the number and subtypes of neurons matches the functional requirements of mature neuronal circuits remains unclear. Here we provide evidence that modulation of programmed cell death during a critical early postnatal period is a regulatory mechanism that controls, in a homeostatic manner, the number of GABAergic interneurons in the mammalian cortex. Our experiments highlight a critical interplay between the physiological state of the network and its cellular units and suggest a feedback mechanism that fine-tunes the size of the CI population to stabilize brain activity.

Early stages of neural development are often characterized by proliferative expansion of progenitors that create a surplus number of neurons that are later eliminated by apoptosis. For example, the size of motor neuron and sympathetic neuron pools is largely determined during development by the availability of limiting amounts of retrograde pro-survival signals supplied by relevant peripheral targets (Davies, 2003; Oppenheim, 1991). However, the neurotrophic factor paradigm cannot adequately explain the regulation of apoptosis in most regions of the CNS where alternative pathways have been implicated (Dekkers et al., 2013). *In vivo* and *in vitro* studies have demonstrated that survival of cortical PNs is enhanced by network activity and that N-methyl-D-aspartate (NMDA) receptor-mediated synaptic currents modulate rates of apoptosis (Blanquie et al., 2017; Ikonomidou et al., 1999; reviewed by Bell and Hardingham, 2011). In addition, apoptosis of adult-generated neurons, such as olfactory bulb interneurons and dentate gyrus granule cells, can be dramatically influenced by the activity of the mature networks into which they integrate (Bovetti et al., 2009; Corotto et al., 1994; Mu et al., 2015; Petreanu and Alvarez-Buylla, 2002; Rochefort et al., 2002; Tashiro et al., 2006), probably through a cell-autonomous mechanism (Lin et al., 2010). Contrasting these studies, Southwell et al. (2012)

demonstrated recently that, in rodents, a large fraction of CIs (~40%) are eliminated by a program of apoptosis that is intrinsic to this cell lineage. Our genetic lineage tracing experiments in *Lhx6* mutants argue against a rigid and intrinsically determined program of apoptosis of CI progenitors and suggest a considerable degree of developmental plasticity driven ultimately by the physiological state of the network. This view is supported by the preferential survival of either wild-type CI progenitors grafted into the hyperactive cortex of *Lhx6*-deficient animals (Figure 3) or by chemogenetically activated MGE-derived CIs grafted into the cortex of wild-type animals (Figure 5). Together with the modulation of endogenous CI numbers in the *Lhx6* mutant cortex, our findings suggest an overarching mechanism for the control CI number in the pallium during development, whereby inhibitory interneurons monitor the activity of their local environment and adjust the level of apoptosis in a cell-autonomous manner. Interestingly, the enhanced apoptosis of immature CIs observed in response to pharmacological inhibition of NMDA receptors (Roux et al., 2015) suggests that excitatory glutamatergic neurotransmission may play a role in this regulatory pro-survival response. In line with this view, cell autonomous increases in the activity of CIs improves their survival through a calcineurin-mediated pathway (Priya et al., 2018).

Other studies have also shown apparent compensatory forms of plasticity in response to the loss of CI subtypes. Deletion of the *Lhx6*-dependent effector gene *Sox6* in postmitotic immature interneurons was associated with a dramatic decrease in the number of Pv^+ and Sst^+ interneurons but no change in the total number of CIs (Azim et al., 2009; Batista-Brito et al., 2009). Although an increase of NPY^+ interneurons was reported in these studies, the mechanisms that maintain the total number of CIs in *Sox6* mutants remain unclear. Also, conditional inactivation of the orphan nuclear receptor *Nrf1* (*COUP-TFI*) in interneuron progenitors resulted in a decreased number of CR^+ and VIP^+ CIs and a concomitant increase of PV- and NPY-expressing subtypes without affecting the total number of GABAergic interneurons in the cortex (Lodato et al., 2011). Although this compensation was thought to result from enhanced proliferation of CI progenitors, it is possible that changes in apoptosis also contribute to this phenotype. Our data, in agreement with Priya et al. (2018), show that regulation of CI apoptosis is subtype-specific. For example, among the *Lhx6*-independent CGE-generated CIs, only $Reelin^+$ neurons increase in number following *Lhx6* deletion (Figures 1M and S2). The subtype-specific response of CIs to activity is not solely restricted to apoptosis because recent studies have shown that silencing CGE-derived interneurons results in defects in radial migration, cell morphology, and synaptic development of $Reelin^+$ but not VIP^+ CIs (De Marco García et al., 2011, 2015). Although it has also been suggested that MGE-derived CIs are more sensitive to hyperpolarization than CGE-derived CIs (Close et al., 2012), we find that CGE-derived CIs strongly modulate their levels of apoptosis in *Lhx6* mutants (Figure 2T). However, whether this is a result of increases in high network activity (Figures 4E–4L) or by a different molecular pathway still remains to be established. Further analysis of the set of candidate genes we identified in CGE-derived CIs isolated by flow cytometry may help clarify the mechanisms behind the cell-specific modulation of apoptosis in CIs. Our results also shed new light on the many functions of *Lhx6* in the development of MGE-derived CIs. Here, we show that MGE CIs lacking *Lhx6* have increased rates of apoptosis from early post-natal stages (certainly by P2; Figures 2S and 2U), with only a small fraction of CIs undergoing a previously observed change to a CGE fate (Figures S1M and S1N; Vogt et al., 2014). However, it is unclear what role the remaining MGE-derived mutant cells play during development. Our results suggest that the majority of these cells fail to integrate into a functional network, as evidenced by their abnormal accumulation in the cortical margins and high rates of apoptosis.

Our results provide evidence for a simple mechanism that controls the number of inhibitory interneurons in the cortex. We propose that the temporal overlap between developmental programs that dictate the size (and thus the functional output) of the CI complement and the emerging activity of cortical networks allows for the engagement and cross-regulation of the two processes until an optimal activity set point is attained. Several preclinical models of CI-based cell therapies have been established for the treatment of epileptic seizures (Alvarez Dolado and Broccoli, 2011; Southwell et al., 2014; Tyson and Anderson, 2014). Our present data argue that increased activity levels in the host brain, typically observed in epileptic encephalopathy mouse models (Batista-Brito et al., 2009; Hedrich et al., 2014), or increased activity in transplanted CIs will provide favorable conditions for the survival of grafted CI progenitors. Characterizing the pro-survival patterns of neuronal activity and identifying the CI subtypes best suited for transplantation may improve the effectiveness of these nascent therapies.

EXPERIMENTAL PROCEDURES

Animals

All procedures involving mice were approved by the ethical review panel at King's College London (KCL) and National Institute for Medical Research in accordance with the United Kingdom Animals (Scientific Procedures) Act (1986). A conditional *Lhx6* allele (*Lhx6*^{fl}) was generated via homologous recombination using a targeting construct in which loxP sites were placed in non-coding regions just 5′ to coding exon 1b and 3′ to coding exon 3 (Figure S1A). The Tg(Nkx2-1-cre)1Wdr (Mouse Genome Informatics [MGI]:3761164, shortened here as *Nkx2.1-Cre*; Kessaris et al., 2006), Slc32a1$^{tm2(cre)Lowl}$ (MGI:5141270, shortened here as *VGat-Cre*; Vong et al., 2011), Tg(Htr3a-cre)NO152Gsat (MGI:5435492, shortened here as *Htr3a-Cre*, generated by The Gene Expression Nervous System Atlas [GENSAT] Project, The Rockefeller University, New York), and Gt(ROSA)26Sor$^{tm14(CAG-tdTomato)Hze}$ (MGI:3809524, shortened here as Ai14; Madisen et al., 2010), Lhx6^{tm2Vpa} (MGI:3702518, shortened here as *Lhx6*$^{-}$; Liodis et al., 2007), and *Lhx6*^{fl} animals were maintained on a mixed background and genotyped as described previously. Both male and female mice were used in all experiments.

GE Cell Transplantations

Both medial and caudal ganglionic eminences were dissected from E14.5 Gad1$^{tm1.1Tama}$ (MGI:3590301, shortened here as *GAD67-GFP*; Tamamaki et al., 2003) heterozygote embryos, dissociated as previously described (Du et al., 2008), and the resulting cell suspension was grafted into the cortices of control (*Lhx6*$^{+/-}$) and mutant (*Lhx6*$^{-/-}$) neonatal pups (P0–P1). One single injection was made into the cortex of each pup. The same needle was used for all injections to pups of the same litter, and between injections, the needle was inspected to verify that the same cell suspension volume was injected. Grafted animals were transcardially perfused at P16. Only litters containing at least one mutant and one control mouse were analyzed, and values for mutants were normalized to the average number found in the control littermates, injected with the same cell suspension.

MGE Electroporation and Cell Transplantations

Ex vivo electroporation of MGE in embryonic brain slices (E14) was conducted as described previously (Stühmer et al., 2002). Twelve hours after electroporation of a mixture of pCAGGS-hM3D(Gq)-IRES (internal ribosome entry site)-RFP and pCAGGS-IRES-GFP plasmids, the MGE regions with the strongest GFP signal were dissociated as described previously (Du et al., 2008). The resulting cell suspension was grafted into neonatal (P0–P1) cortices of wild-type mice (6 injections per brain/3 per hemisphere) as described above. Cohorts of littermates grafted with the same cell suspension were divided in two groups: one group was injected intraperitoneally twice per day (every 12 hr) with 1 mg/kg CNO (Tocris Bioscience) (diluted in vehicle: 0.5% DMSO containing saline), whereas a control group was injected with vehicle only from P14 until P17 (one injection only at P17). Mice where then transcardially perfused within 1 hr from the last injection, and dissected brains were processed for immunohistochemistry. Only experiments where GFP^+ cells were identified in at least one animal from each group were analyzed.

Statistical Methods

All mean, SE, and statistical tests were calculated using standard statistical routines in Igor Pro or Excel. n is taken as the number of animals. In Table S1, we present detailed information of the statistical tests used to assess significance.

DATA AND SOFTWARE AVAILABILITY

The accession number for the RNA-seq results reported in this paper is GEO: GSE108161.

SUPPLEMENTAL INFORMATION

Supplemental Information includes Supplemental Experimental Procedures, five figures, and three tables and can be found with this article online at https://doi.org/10.1016/j.celrep.2018.01.064.

ACKNOWLEDGMENTS

This work was supported by ERC Starter Grant 282047, Wellcome Trust Investigator Award 095589/Z/11/Z, an FP7 EC DESIRE grant, and a Lister Institute prize (to J.B.). Work in V.P.'s laboratory is supported by the BBSRC (BB/L022974/1), the UK Medical Research Council (MRC), and the Francis Crick Institute (which receives funding from the MRC, Cancer Research UK, and the Wellcome Trust).

AUTHOR CONTRIBUTIONS

M.D., G.N., J.B., and V.P. conceived the experiments. M.D. and G.N. performed most experiments and analyzed the data. A.R. analyzed the RNA-seq data. P.L. created and characterized molecularly the *Lhx6*$^{-}$ and *Lhx6*^{fl} alleles. S.K. performed *in utero* electroporations of hM3D(Gq) expression plasmids and analyzed the data described in Figure S5. M.D., G.N., J.B., and V.P. wrote the paper with input from all the authors.

DECLARATION OF INTERESTS

The authors declare no competing interests.

Received: September 27, 2017
Revised: December 18, 2017
Accepted: January 22, 2018
Published: February 13, 2018

REFERENCES

Alvarez Dolado, M., and Broccoli, V. (2011). GABAergic neuronal precursor grafting: implications in brain regeneration and plasticity. Neural Plast. *2011*, 384216.

Azim, E., Jabaudon, D., Fame, R.M., and Macklis, J.D. (2009). SOX6 controls dorsal progenitor identity and interneuron diversity during neocortical development. Nat. Neurosci. *12*, 1238–1247.

Bartolini, G., Ciceri, G., and Marín, O. (2013). Integration of GABAergic interneurons into cortical cell assemblies: lessons from embryos and adults. Neuron *79*, 849–864.

Batista-Brito, R., Rossignol, E., Hjerling-Leffler, J., Denaxa, M., Wegner, M., Lefebvre, V., Pachnis, V., and Fishell, G. (2009). The cell-intrinsic requirement of Sox6 for cortical interneuron development. Neuron *63*, 466–481.

Bell, K.F., and Hardingham, G.E. (2011). The influence of synaptic activity on neuronal health. Curr. Opin. Neurobiol. *21*, 299–305.

Blanquie, O., Yang, J.W., Kilb, W., Sharopov, S., Sinning, A., and Luhmann, H.J. (2017). Electrical activity controls area-specific expression of neuronal apoptosis in the mouse developing cerebral cortex. eLife *6*, e27696.

Bloodgood, B.L., Sharma, N., Browne, H.A., Trepman, A.Z., and Greenberg, M.E. (2013). The activity-dependent transcription factor NPAS4 regulates domain-specific inhibition. Nature *503*, 121–125.

Bovetti, S., Veyrac, A., Peretto, P., Fasolo, A., and De Marchis, S. (2009). Olfactory enrichment influences adult neurogenesis modulating GAD67 and plasticity-related molecules expression in newborn cells of the olfactory bulb. PLoS ONE *4*, e6359.

Close, J., Xu, H., De Marco García, N., Batista-Brito, R., Rossignol, E., Rudy, B., and Fishell, G. (2012). Satb1 is an activity-modulated transcription factor required for the terminal differentiation and connectivity of medial ganglionic eminence-derived cortical interneurons. J. Neurosci. *32*, 17690–17705.

Cohen, S., and Greenberg, M.E. (2008). Communication between the synapse and the nucleus in neuronal development, plasticity, and disease. Annu. Rev. Cell Dev. Biol. *24*, 183–209.

Corotto, F.S., Henegar, J.R., and Maruniak, J.A. (1994). Odor deprivation leads to reduced neurogenesis and reduced neuronal survival in the olfactory bulb of the adult mouse. Neuroscience *61*, 739–744.

Davies, A.M. (2003). Regulation of neuronal survival and death by extracellular signals during development. EMBO J. *22*, 2537–2545.

De Marco García, N.V., Karayannis, T., and Fishell, G. (2011). Neuronal activity is required for the development of specific cortical interneuron subtypes. Nature *472*, 351–355.

De Marco García, N.V., Priya, R., Tuncdemir, S.N., Fishell, G., and Karayannis, T. (2015). Sensory inputs control the integration of neurogliaform interneurons into cortical circuits. Nat. Neurosci. *18*, 393–401.

Dekkers, M.P., Nikoletopoulou, V., and Barde, Y.A. (2013). Cell biology in neuroscience: Death of developing neurons: new insights and implications for connectivity. J. Cell Biol. *203*, 385–393.

DeSteno, D.A., and Schmauss, C. (2008). Induction of early growth response gene 2 expression in the forebrain of mice performing an attention-set-shifting task. Neuroscience *152*, 417–428.

Du, T., Xu, Q., Ocbina, P.J., and Anderson, S.A. (2008). NKX2.1 specifies cortical interneuron fate by activating Lhx6. Development *135*, 1559–1567.

Eagle, A.L., Gajewski, P.A., Yang, M., Kechner, M.E., Al Masraf, B.S., Kennedy, P.J., Wang, H., Mazei-Robison, M.S., and Robison, A.J. (2015). Experience-Dependent Induction of Hippocampal ΔFosB Controls Learning. J. Neurosci. *35*, 13773–13783.

Fishell, G., and Rudy, B. (2011). Mechanisms of inhibition within the telencephalon: "where the wild things are". Annu. Rev. Neurosci. *34*, 535–567.

Fogarty, M., Grist, M., Gelman, D., Marín, O., Pachnis, V., and Kessaris, N. (2007). Spatial genetic patterning of the embryonic neuroepithelium generates GABAergic interneuron diversity in the adult cortex. J. Neurosci. *27*, 10935–10946.

French, P.J., O'Connor, V., Jones, M.W., Davis, S., Errington, M.L., Voss, K., Truchet, B., Wotjak, C., Stean, T., Doyère, V., et al. (2001). Subfield-specific immediate early gene expression associated with hippocampal long-term potentiation in vivo. Eur. J. Neurosci. *13*, 968–976.

Gall, C., Lauterborn, J., Isackson, P., and White, J. (1990). Seizures, neuropeptide regulation, and mRNA expression in the hippocampus. Prog. Brain Res. *83*, 371–390.

Gelman, D., Griveau, A., Dehorter, N., Teissier, A., Varela, C., Pla, R., Pierani, A., and Marín, O. (2011). A wide diversity of cortical GABAergic interneurons derives from the embryonic preoptic area. J. Neurosci. *31*, 16570–16580.

Hartmann, M., Heumann, R., and Lessmann, V. (2001). Synaptic secretion of BDNF after high-frequency stimulation of glutamatergic synapses. EMBO J. *20*, 5887–5897.

Hedrich, U.B., Liautard, C., Kirschenbaum, D., Pofahl, M., Lavigne, J., Liu, Y., Theiss, S., Slotta, J., Escayg, A., Dihné, M., et al. (2014). Impaired action potential initiation in GABAergic interneurons causes hyperexcitable networks in an epileptic mouse model carrying a human Na(V)1.1 mutation. J. Neurosci. *34*, 14874–14889.

Ikonomidou, C., Bosch, F., Miksa, M., Bittigau, P., Vöckler, J., Dikranian, K., Tenkova, T.I., Stefovska, V., Turski, L., and Olney, J.W. (1999). Blockade of NMDA receptors and apoptotic neurodegeneration in the developing brain. Science *283*, 70–74.

Kepecs, A., and Fishell, G. (2014). Interneuron cell types are fit to function. Nature *505*, 318–326.

Kessaris, N., Fogarty, M., Iannarelli, P., Grist, M., Wegner, M., and Richardson, W.D. (2006). Competing waves of oligodendrocytes in the forebrain and postnatal elimination of an embryonic lineage. Nat. Neurosci. *9*, 173–179.

Lee, S., Hjerling-Leffler, J., Zagha, E., Fishell, G., and Rudy, B. (2010). The largest group of superficial neocortical GABAergic interneurons expresses ionotropic serotonin receptors. J. Neurosci. *30*, 16796–16808.

Li, L., Yun, S.H., Keblesh, J., Trommer, B.L., Xiong, H., Radulovic, J., and Tourtellotte, W.G. (2007). Egr3, a synaptic activity regulated transcription factor that is essential for learning and memory. Mol. Cell. Neurosci. *35*, 76–88.

Lin, C.W., Sim, S., Ainsworth, A., Okada, M., Kelsch, W., and Lois, C. (2010). Genetically increased cell-intrinsic excitability enhances neuronal integration into adult brain circuits. Neuron *65*, 32–39.

Liodis, P., Denaxa, M., Grigoriou, M., Akufo-Addo, C., Yanagawa, Y., and Pachnis, V. (2007). Lhx6 activity is required for the normal migration and specification of cortical interneuron subtypes. J. Neurosci. *27*, 3078–3089.

Lodato, S., Tomassy, G.S., De Leonibus, E., Uzcategui, Y.G., Andolfi, G., Armentano, M., Touzot, A., Gaztelu, J.M., Arlotta, P., Menendez de la Prida, L., and Studer, M. (2011). Loss of COUP-TFI alters the balance between caudal ganglionic eminence- and medial ganglionic eminence-derived cortical interneurons and results in resistance to epilepsy. J. Neurosci. *31*, 4650–4662.

Ma, T., Zhang, Q., Cai, Y., You, Y., Rubenstein, J.L., and Yang, Z. (2012). A subpopulation of dorsal lateral/caudal ganglionic eminence-derived neocortical interneurons expresses the transcription factor Sp8. Cereb. Cortex *22*, 2120–2130.

Madisen, L., Zwingman, T.A., Sunkin, S.M., Oh, S.W., Zariwala, H.A., Gu, H., Ng, L.L., Palmiter, R.D., Hawrylycz, M.J., Jones, A.R., et al. (2010). A robust and high-throughput Cre reporting and characterization system for the whole mouse brain. Nat. Neurosci. *13*, 133–140.

Mardinly, A.R., Spiegel, I., Patrizi, A., Centofante, E., Bazinet, J.E., Tzeng, C.P., Mandel-Brehm, C., Harmin, D.A., Adesnik, H., Fagiolini, M., and Greenberg, M.E. (2016). Sensory experience regulates cortical inhibition by inducing IGF1 in VIP neurons. Nature *531*, 371–375.

Marín, O. (2012). Interneuron dysfunction in psychiatric disorders. Nat. Rev. Neurosci. *13*, 107–120.

Marín, O., and Rubenstein, J.L. (2001). A long, remarkable journey: tangential migration in the telencephalon. Nat. Rev. Neurosci. *2*, 780–790.

Meeker, R.B., and Williams, K.S. (2015). The p75 neurotrophin receptor: at the crossroad of neural repair and death. Neural Regen. Res. *10*, 721–725.

Miyoshi, G., and Fishell, G. (2011). GABAergic interneuron lineages selectively sort into specific cortical layers during early postnatal development. Cereb. Cortex *21*, 845–852.

Mu, Y., Zhao, C., Toni, N., Yao, J., and Gage, F.H. (2015). Distinct roles of NMDA receptors at different stages of granule cell development in the adult brain. eLife *4*, e07871.

Neves, G., Shah, M.M., Liodis, P., Achimastou, A., Denaxa, M., Roalfe, G., Sesay, A., Walker, M.C., and Pachnis, V. (2013). The LIM homeodomain protein Lhx6 regulates maturation of interneurons and network excitability in the mammalian cortex. Cereb. Cortex *23*, 1811–1823.

Oppenheim, R.W. (1991). Cell death during development of the nervous system. Annu. Rev. Neurosci. *14*, 453–501.

Petreanu, L., and Alvarez-Buylla, A. (2002). Maturation and death of adult-born olfactory bulb granule neurons: role of olfaction. J. Neurosci. *22*, 6106–6113.

Qiu, J., McQueen, J., Bilican, B., Dando, O., Magnani, D., Punovuori, K., Selvaraj, B.T., Livesey, M., Haghi, G., Heron, S., et al. (2016). Evidence for evolutionary divergence of activity-dependent gene expression in developing neurons. eLife *5*, e20337.

Priya, R., Paredes, M.F., Karayannis, T., Yusuf, N., Liu, X., Jaglin, X., Graef, I., Alvarez-Buylla, A., and Fishell, G. (2018). Activity regulates cell death within cortical interneurons through a calcineurin-dependent mechanism. Cell Rep. *22*, this issue, 1695–1709.

Rochefort, C., Gheusi, G., Vincent, J.D., and Lledo, P.M. (2002). Enriched odor exposure increases the number of newborn neurons in the adult olfactory bulb and improves odor memory. J. Neurosci. *22*, 2679–2689.

Roux, C., Aligny, C., Lesueur, C., Girault, V., Brunel, V., Ramdani, Y., Genty, D., Driouich, A., Laquerrière, A., Marret, S., et al. (2015). NMDA receptor blockade in the developing cortex induces autophagy-mediated death of immature cortical GABAergic interneurons: An ex vivo and in vivo study in Gad67-GFP mice. Exp. Neurol. *267*, 177–193.

Rubenstein, J.L., and Merzenich, M.M. (2003). Model of autism: increased ratio of excitation/inhibition in key neural systems. Genes Brain Behav. *2*, 255–267.

Southwell, D.G., Paredes, M.F., Galvao, R.P., Jones, D.L., Froemke, R.C., Sebe, J.Y., Alfaro-Cervello, C., Tang, Y., Garcia-Verdugo, J.M., Rubenstein, J.L., et al. (2012). Intrinsically determined cell death of developing cortical interneurons. Nature *491*, 109–113.

Southwell, D.G., Nicholas, C.R., Basbaum, A.I., Stryker, M.P., Kriegstein, A.R., Rubenstein, J.L., and Alvarez-Buylla, A. (2014). Interneurons from embryonic development to cell-based therapy. Science *344*, 1240622.

Stühmer, T., Puelles, L., Ekker, M., and Rubenstein, J.L. (2002). Expression from a Dlx gene enhancer marks adult mouse cortical GABAergic neurons. Cereb. Cortex *12*, 75–85.

Tamamaki, N., Yanagawa, Y., Tomioka, R., Miyazaki, J., Obata, K., and Kaneko, T. (2003). Green fluorescent protein expression and colocalization with calretinin, parvalbumin, and somatostatin in the GAD67-GFP knock-in mouse. J. Comp. Neurol. *467*, 60–79.

Tashiro, A., Sandler, V.M., Toni, N., Zhao, C., and Gage, F.H. (2006). NMDA-receptor-mediated, cell-specific integration of new neurons in adult dentate gyrus. Nature *442*, 929–933.

Turrigiano, G. (2012). Homeostatic synaptic plasticity: local and global mechanisms for stabilizing neuronal function. Cold Spring Harb. Perspect. Biol. *4*, a005736.

Tyson, J.A., and Anderson, S.A. (2014). GABAergic interneuron transplants to study development and treat disease. Trends Neurosci. *37*, 169–177.

Tzingounis, A.V., and Nicoll, R.A. (2006). Arc/Arg3.1: linking gene expression to synaptic plasticity and memory. Neuron *52*, 403–407.

Urban, D.J., and Roth, B.L. (2015). DREADDs (designer receptors exclusively activated by designer drugs): chemogenetic tools with therapeutic utility. Annu. Rev. Pharmacol. Toxicol. *55*, 399–417.

Vogt, D., Hunt, R.F., Mandal, S., Sandberg, M., Silberberg, S.N., Nagasawa, T., Yang, Z., Baraban, S.C., and Rubenstein, J.L. (2014). Lhx6 directly

regulates Arx and CXCR7 to determine cortical interneuron fate and laminar position. Neuron *82*, 350–364.

Vong, L., Ye, C., Yang, Z., Choi, B., Chua, S., Jr., and Lowell, B.B. (2011). Leptin action on GABAergic neurons prevents obesity and reduces inhibitory tone to POMC neurons. Neuron *71*, 142–154.

Wonders, C.P., and Anderson, S.A. (2006). The origin and specification of cortical interneurons. Nat. Rev. Neurosci. *7*, 687–696.

Yamaguchi, Y., and Miura, M. (2015). Programmed cell death in neurodevelopment. Dev. Cell *32*, 478–490.

Yu, H., Su, Y., Shin, J., Zhong, C., Guo, J.U., Weng, Y.L., Gao, F., Geschwind, D.H., Coppola, G., Ming, G.L., and Song, H. (2015). Tet3 regulates synaptic transmission and homeostatic plasticity via DNA oxidation and repair. Nat. Neurosci. *18*, 836–843.

Zhao, Y., Flandin, P., Long, J.E., Cuesta, M.D., Westphal, H., and Rubenstein, J.L. (2008). Distinct molecular pathways for development of telencephalic interneuron subtypes revealed through analysis of Lhx6 mutants. J. Comp. Neurol. *510*, 79–99.

Cell Reports, Volume *22*

Supplemental Information

Modulation of Apoptosis Controls Inhibitory Interneuron Number in the Cortex

Myrto Denaxa, Guilherme Neves, Adam Rabinowitz, Sarah Kemlo, Petros Liodis, Juan Burrone, and Vassilis Pachnis

Supplemental Experimental Procedures:

Lhx6 conditional mice and genotyping: A conditional *Lhx6* allele was generated via homologous recombination using a targeting construct in which loxP sites were placed in non-coding regions just 5' to coding exon 1b, and 3' to coding exon 3 (Figure S1 A). The 5' homology is made up from a 3.1 kb *XbaI–Sac*I fragment containing the 5' upstream region and the first exon (1a) of *Lhx6*, whereas the 3' homology is made up from a 5.4 kb *ApaI–NheI* fragment. For the *Lhx6* targeting vector, the 2 kb genomic fragment between the homology regions is replaced by a 4 kb cassette, containing the following: (1) the 1b, 2 and 3 coding exons flanked by loxP sites (2) the neomycin resistance gene under the control of the phosphoglycerate kinase (PGK) promoter (PGK-Neo) flanked by FRT sites. Targeting constructs were linearized and electroporated into E14Tg2A embryonic stem (ES) cells. Targeted clones were identified and analyzed in detail by Southern blotting using the 5' and 3' external probes indicated in Figure S1B. Germline transmission of the mutant alleles was achieved using standard protocols. The phenotypic analysis presented was performed on animals from which the PGK-Neo cassette was removed by crossing founder $Lhx6^{Fl/+}$ animals with the *Tg(ACTFLPe)9205Dym* transgenic line (MGI:2448985, Rodriguez et al., 2000). Deletion was confirmed using Southern blotting analysis. For the maintenance of the $Lhx6^{Fl}$ colony, we performed PCR using the following primers: F:5'-CTCGAGTGCTCCGTGTGTC-3', R:5'-GGAGGCCCAAAGTTAGAACC-3'.

Animals: Animals were bred and housed in accordance with the United Kingdom Animals (Scientific Procedures) Act (1986). The *Tg(Nkx2-1-cre)1Wdr* (MGI:3761164, shortened here as *Nkx2.1Cre,* Kessaris et al., 2006), $Slc32a1^{tm2(cre)Lowl}$ (MGI:5141270, shortened here as *VGatCre*, Vong et al., 2011), *Tg(Htr3a-cre)NO152Gsat* (MGI:5435492, shortened here as *Htr3aCre*, generated by The Gene Expression Nervous System Atlas –GENSAT - Project, The Rockefeller University - New York), $Gt(ROSA)26Sor^{tm14(CAG\text{-}tdTomato)Hze}$ (MGI:3809524, shortened here as *Ai14*, Madisen et al., 2010). $Lhx6^{tm2Vpa}$ (MGI:3702518, shortened here as $Lhx6^{-}$, Liodis et al., 2007) and $Lhx6^{fl}$ animals were maintained on a mixed background and genotyped as described previously. To generate productive crosses, $Cre\text{-}positive;Lhx6^{+/-}$ males were mated with $Lhx6^{fl/fl};Ai14$ or $Lhx6^{+/-};Ai14$ females and the resulting littermates were analyzed.

Illumina RNA-Seq library preparation, sequencing and analysis: Illumina RNA-Seq libraries were made with 1 µg of total RNA according to the manufacturer's protocol. The only deviation from the protocol was to use the e-gel clone well system (Invitrogen) for fragment size selection. Total RNA was extracted from the forebrain of P15 mice. Each sample was composed of two animals. Four samples for $Lhx6^{+/-}$ and three samples for $Lhx6^{-/-}$ mouse brains were collected and further analysed. 36 base pair single end sequencing was undertaken using an Illumina GA IIx DNA sequencer. FASTQ reads were trimmed with CutAdapt (http://journal.embnet.org/index.php/embnetjournal/article/view/200/479) to remove adapter contamination and low quality sequence. Reads were aligned to the Ensembl GRCm38.84 version of the mouse transcriptome using Bowtie2 (Langmead and Salzberg, 2012). Expected gene counts were extracted from the alignments using RSEM (Li and Dewey, 2011). Differential expression analysis was then perfomed using DESeq2 package (Love et al., 2014). Significance was assigned to genes with an adjusted p-value of <= 0.05. Summary information on differentially epressed genes is provided in Table S2. Significance enrichment of differentially expressed genes with gene lists obtained from other studies were identified using the hypergeometric test. To correct for multiple-hypothesis-testing a false-discovery rate was calculated from the resultant p-values. Heatmaps: The log2-fold-change expression, of each gene in each sample, was calculated relative to the mean expression of the gene across all samples. Hierarchical clustering of the samples was performed on the euclidean distances between the log2-fold-change values for each sample.

Quantification of CGE-derived CI transcripts: For quantification of transcripts in CGE-derived CIs, P7 brains were dissociated (papain dissociation system, Worthington, Lorne) and tdT^{+} cells were isolated by flow cytometry (FACS ARIAII, Becton-Dickinson; typically, a yield of 60,000 cells). Total RNA was purified (RNeasy microKit, Qiagen) and subjected to reverse transcription (RT^2 First Strand, Qiagen) and quantitative PCR according to manufacturer's instructions (RT^2 Profiler PCR plasticity pathway-focused gene expression array, Qiagen). Results presented in Table S3 represent were obtained from two $Htr3aCre;Ai14;Lhx6^{-/-}$ and two $Htr3aCre;Ai14;Lhx6^{+/-}$ samples.

Expression constructs: Full length cDNA for hM3D(Gq) from plasmid pAAV-hSyn-HA-hM3D(Gq)-IRES-mCitrine (Addgene, 50463), was cloned into a modified pCAGGS-IRES-RFP vector (a gift from Francois Guillemot - CRICK), resulting in pCAGGS-hM3D(Gq)-IRES-RFP. The pCAGGS-IRES-GFP was a gift from James Briscoe (CRICK).

GE cell transplantations: Both medial and caudal ganglionic eminences were dissected from E14.5 $Gad1^{tm1.1Tama}$ (MGI:3590301, shortened here as GAD67::GFP, Tamamaki et al., 2003) heterozygote embryos,

dissociated as previously described (Du et al., 2008), and the resulting cell suspension was grafted into the cortices of *Lhx6* control and mutant neonatal pups (P0-P1). One single injection has been performed into the cortex of each pup, by using the micro-injector unit set-up from the VEVO injection system (VisualSonics). Each injection (69 nl) has been performed at a slow injection rate (23nL/sec) with needles according to the recommended dimensions of the manufacturer. The same needle has been used for all injections to pups of the same litter, and between each injection the needle has been inspected to verify the same cell suspension volume was injected. Grafted animals were transcardially perfused at P16, and dissected brains were processed for immunohistochemistry. Only litters containing at least one *Lhx6*$^{-/-}$ (mutant) and one *Lhx6*$^{+/-}$ (control) mouse were analysed, and values for mutants were normalized to the average number found in the control littermates, injected with the same cell suspension.

<u>*MGE electroporation and cell transplantations:*</u> *Ex vivo* electroporation of MGE in embryonic brain slices (E14) was conducted as described previously (Stuhmer et al., 2002). Twelve hours after electroporation of a mixture of pCAGGS-hM3D(Gq)-IRES-RFP and pCAGGS-IRES-GFP plasmids, MGE regions with the strongest GFP signal were dissociated as previously described (Du et al., 2008). The resulting cell suspension was grafted into neonatal (P0-P1) cortices of wild type mice (6 injections per brain/3 per hemisphere) as described above. Cohorts of littermates grafted with the same cell suspension were divided in two groups: one group was injected intraperitoneally twice per day (every 12 hrs) with 1mg/kg CNO (Tocris Bioscience) (diluted in vehicle - 0.5% DMSO containing saline); while a control group was injected with vehicle only, from P14 until P17 (one injection only at P17). Mice where then transcardially perfused within 1 hr from the last injection, and dissected brains were processed for immunohistochemistry. Only experiments where GFP^{+} cells were identified in at least one animal from each group were analysed.

<u>*In utero elecroporations: I*</u>*n utero* electroporation was carried out using a protocol adapted from (Saito and Nakatsuji, 2001). Briefly, timed-pregnant wild type CD-1 female mice were anesthetized with a mix of oxygen-isoflurane before the abdomen was opened and the uterine horns exposed. The DNA solution was injected into the lateral ventricle of E14.5 embryos using a glass micropipette. Approximately 0.5 microliters of a solution containing Tris-HCl (10 mM), ethylenediaminetetraacetic acid (EDTA, 1 mM), Fast green dye (0.5% w/v) and a mixture (3:1 molar ratio) of pCAGGS-hM3D(Gq)-IRES-RFP and pCAGGS-IRES-GFP plasmids (total DNA concentration ≈ 1 mg/ml) was injected. Five square electric pulses (40 V, 50 ms) were passed at 1 s intervals using a square-wave electroporator (CUY21EDIT, NEPA GENE Co.). At P21 a group of mice received an intraperitoneal injection of 1mg/kg CNO (Tocris Bioscience) (diluted in vehicle - 0.5% DMSO containing saline) and were transcardially perfused and processed for immunohistochemichestry with GFP, RFP and cfos antibodies. A group of control mice did not receive CNO injections.

<u>*Immunostaining:*</u> For immunostaining on brain sections from P16 mice or older, animals were transcardially perfused with 4% PFA and brains were post-fixed overnight (O/N). Vibratome (60 or 100 μm) sections were permeabilized with PBT [0.5% Triton X-100 in PBS (0.5% PBT)] for 1 hr at room temperature (RT), blocked in 10% FCS in PBT (0.3% Triton X-100 in PBS; 2 hrs; RT), and incubated with primary antibodies diluted in blocking solution at 4°C, O/N. After 3 washes with PBT, sections were incubated with secondary antibodies diluted in blocking solution at RT for 2 hrs, washed in PBT, and mounted using Vectashield (Vector) medium. For immunostaining on brain sections from embryos (E14.5, E16.5) or P2/P7 mouse pups, dissected brains were fixed in 4% PFA in PBS at 4°C, O/N. Cryostat sections (14 μm) were permeabilized in 0.1% Triton X-100 in PBS (0.1%PBT) for 5 min, and processed as above. The following antibodies were used: rabbit polyclonal anti-cFos (Calbiochem; 1/10.000), mouse monoclonal anti-cFOS (Santa-Cruz, sc-166940; 1/500), rabbit polyclonal anti-GFP (Invitrogen; 1/1000), rat monoclonal anti-GFP (Invitrogen; 1/1000), mouse monoclonal anti-GFP (Invitrogen; 1/1000), rabbit polyclonal anti-Lhx6 (Lavdas et al., 1999; 1/250–1000), rabbit polyclonal anti-Parvalbumin (Swant, PV25; 1/1000), goat polyclonal anti-Parvalbumin (Swant, PV213; 1/1000), rabbit anti-PH3 (Millipore, 05-636; 1/200), rabbit polyclonal anti-RFP (Abcam; 1/500), mouse monoclonal anti-Reelin (Millipore, MAB5364; 1/500), rabbit polyclonal anti-Sox6 (Abcam, AB30455; 1/4000), goat polyclonal anti-Sp8 (Santa-Cruz, sc-104661; 1/1000), rat monoclonal anti-SST (Millipore, MAB354; 1/1000), rabbit polyclonal anti-VIP (Immunostar, 20077; 1/1000). Secondary antibodies used were as follows: Alexa Fluor 488-conjugated donkey anti-mouse, anti-rat, anti-goat and anti-rabbit and Alexa Fluor 568-conjugated donkey anti-mouse, anti-rat, anti-goat and anti-rabbit (all from Invitrogen; all 1/500).

<u>*In situ hybridization histochemistry (ISHH):*</u> ISHH was carried out essentially as described (Schaeren-Wiemers and Gerfin-Moser, 1993). The Arc-specific and Egr1-specific riboprobes were a gift from Dr. Tahebayashi.

<u>*EdU (5-ethynyl-2'-deoxyuridine) injection/staining*</u>: A stock solution of 10mg/ml EdU (Invitrogen, E10187) was prepared in DPBS (Life technologies, 14190-094). Pregnant females were injected intraperitoneally with 3μl/g weight of each mouse. To study the fraction of cells in the S-phase of the cell cycle, pregnant females were

injected 1 hour prior to embryo harvest. Brain cryosections from E14.5 or E16.5 embryos were first processed for immunohistaining (PH3) and then EdU detection according to the manufacturer's protocol.

*Tunel (terminal deoxynucleotidyl transferase-mediated dUTP nick end-labelling) assay***:** In order to detect cells that are undergoing apoptotic cell death, we used the ApopTag Green *In Situ* Apoptosis Detection Kit (Invitrogen, S7111) and followed manufacturer's instructions. Briefly, immunostained sections were fixed for 10 minutes with 4% PFA, washed with PBS and post-fixed further with an EtOH/Acetic Acid (2:1) mix. Upon equilibration with the appropriate buffer, sections were incubated with TdT enzyme mix for 1hour at 37°C. The reaction was then stopped with STOP buffer, sections were washed and slides were incubated with fluorescein conjugated anti-DIG mix in a dark humidified chamber, for 30 minutes at room temperature. Slides were then washed thoroughly with PBS and mounted with Vectashield-DAPI mounting medium.

Image analysis: Cell counting for all post-natal stages was performed from coronal sections in the cortical region. Images were acquired using a confocal microscope (×20 magnification), taking care for all pairwise comparisons to be acquired in the same imaging session with the same acquisition settings. Cells were manually identified in columnar regions spanning the pial-white matter extent of the cortex across different bregma levels between +2 and -3 mm (as defined in Paxinos et al., 2001) or equivalent regions in the early post-natal stages (shown in Figures 2A-2L). Bregma regions were closely matched for each pairwise comparison. No obvious changes in the reported changes were detected between different bregma regions (e.g. results in Figures 1H-1P are similar to Figure S2). tdT^{+} cell numbers in Figures 1G-1I, S2A and S2D were divided by the total surface area counted (measured in ImageJ) to obtain cell densities (expressed as cells per mm^{2}). For layer distribution analysis (Figure 1J- 1L), the cortical column was divided in the different cortical layers, identified by DAPI nuclear staining. Numbers for each layer are expressed as a fraction of all the cells counted. Some measurements were normalized to the average density observed for control animals in each pairwise comparison. For analysis of specific CI subpopulations (Figures 1M-1P, S2B, S2C, S2E and S2F, separate channels were acquired and markers were initially assessed independently, and were later combined to assess co-localization using the ImageJ software.. Essentially similar methods were used for analysis of apoptosis of fate mapped CI populations (Figures 2S-2U) and characterization of the MGE CI population in *Lhx6* conditional mutants (Figures S1I-S1N), except that cell numbers were normalized to the size of the tdT^{+} population. For Figures 1G-1P analysis was restricted to the primary somatosensory cortex (as defined in Paxinos et al., 2001), while for Figure S2 countings were performed in the primary motor cortex. For Figures 2I-2M, and S1G-S1M countings were performed for at least 4 different matched bregma levels and measurements were averaged to obtain cell densities for each animal. For analysis of survival of grafted GAD67::GFP^{+} CIs (Figure 3I), cells present in the cortex in all sections were counted. Numbers for each litter (independently shown in Figure 3 H) were normalized to the average number found in the *Lhx6*$^{+/-}$ animals, a minimum of 500 cells were counted per brain. For analysis of grafted cell dispersion (Figure 3G), cell counts in each section (for all littermates transplanted with that same cell suspension) to the maximum number of cells observed in the the litter (always a mutant section), We then sorted the sections for each brain by distance (in the anterior-posterior axis) to the section with maximum observed cell count for that brain. For analysis of survival of grafted CIs transfected with hM3D(Gq) expressing constructs (Figure 5J) GFP^{+} cells present in the cortex in all sections were counted and assessed for RFP expression. Numbers of double positive cells (RFP^{+}GFP^{+}) were expressed as a fraction of total GFP^{+} population for each brain, a minimum of 150 cells were counted per brain. In the graph lines connect markers corresponding to animals grafted with the same cell suspension and given different treatments (vehicle or CNO). For analyses of soma size (Figure S4), an oval shaped region of interest (ROI) was drawn just inside the soma region of cells that were judged to be in focus. The area size of the ROIs was measured. Area sizes were measured from at least three different brains in matched bregma levels. For analysis of nuclear cfos expression (Figure S5) images were acquired at 40X magnification. The cfos channel was analysed independently, by drawing oval shaped ROIs inside the nuclear region of all nuclei present in the image, at the Z slice with highest fluorescent intensity. Average fluorescent intensity was measured for all ROIs in both the cfos channel and the RFP channel. Measurements in the cfos channel were divided in two groups based on RFP intensity. All cfos intensity measurements were normalized to the median intensity of the group with RFP expression below threshold. All imaging and analysis were done by an experimenter blind to the experimental condition. All values are presented as mean ± standard error of the mean. All results presented in Figures are presented in the Table S1, accompanied by the statistical test used to assess significance.

Supplemental References:

Du, T., Xu, Q., Ocbina, P.J., and Anderson, S.A. (2008). NKX2.1 specifies cortical interneuron fate by activating Lhx6. Development 135, 1559-1567.

Kessaris, N., Fogarty, M., Iannarelli, P., Grist, M., Wegner, M., and Richardson, W.D. (2006). Competing waves of oligodendrocytes in the forebrain and postnatal elimination of an embryonic lineage. Nat Neurosci 9, 173-179.

Langmead, B., and Salzberg, S.L. (2012). Fast gapped-read alignment with Bowtie 2. Nat Methods 9, 357-359.

Lavdas, A.A., Grigoriou, M., Pachnis, V., and Parnavelas, J.G. (1999). The medial ganglionic eminence gives rise to a population of early neurons in the developing cerebral cortex [In Process Citation]. J Neurosci 19, 7881-7888.

Li, B., and Dewey, C.N. (2011). RSEM: accurate transcript quantification from RNA-Seq data with or without a reference genome. BMC Bioinformatics 12, 323.

Liodis, P., Denaxa, M., Grigoriou, M., Akufo-Addo, C., Yanagawa, Y., and Pachnis, V. (2007). Lhx6 activity is required for the normal migration and specification of cortical interneuron subtypes. J Neurosci 27, 3078-3089.

Love, M.I., Huber, W., and Anders, S. (2014). Moderated estimation of fold change and dispersion for RNA-seq data with DESeq2. Genome Biol 15, 550.

Madisen, L., Zwingman, T.A., Sunkin, S.M., Oh, S.W., Zariwala, H.A., Gu, H., Ng, L.L., Palmiter, R.D., Hawrylycz, M.J., Jones, A.R., *et al.* (2010). A robust and high-throughput Cre reporting and characterization system for the whole mouse brain. Nat Neurosci 13, 133-140.

Paxinos, G., Franklin, K.B.J., and Franklin, K.B.J. (2001). The mouse brain in stereotaxic coordinates, 2nd edn (San Diego: Academic Press).

Rodriguez, C.I., Buchholz, F., Galloway, J., Sequerra, R., Kasper, J., Ayala, R., Stewart, A.F., and Dymecki, S.M. (2000). High-efficiency deleter mice show that FLPe is an alternative to Cre-loxP. Nat Genet 25, 139-140.

Saito, T., and Nakatsuji, N. (2001). Efficient gene transfer into the embryonic mouse brain using in vivo electroporation. Dev Biol 240, 237-246.

Schaeren-Wiemers, N., and Gerfin-Moser, A. (1993). A single protocol to detect transcripts of various types and expression levels in neural tissue and cultured cells: in situ hybridization using digoxigenin-labelled cRNA probes. Histochemistry 100, 431-440.

Stuhmer, T., Puelles, L., Ekker, M., and Rubenstein, J.L. (2002). Expression from a Dlx gene enhancer marks adult mouse cortical GABAergic neurons. Cereb Cortex 12, 75-85.

Tamamaki, N., Yanagawa, Y., Tomioka, R., Miyazaki, J., Obata, K., and Kaneko, T. (2003). Green fluorescent protein expression and colocalization with calretinin, parvalbumin, and somatostatin in the GAD67-GFP knock-in mouse. J Comp Neurol 467, 60-79.

Vong, L., Ye, C., Yang, Z., Choi, B., Chua, S., Jr., and Lowell, B.B. (2011). Leptin action on GABAergic neurons prevents obesity and reduces inhibitory tone to POMC neurons. Neuron 71, 142-154.

Figure S1

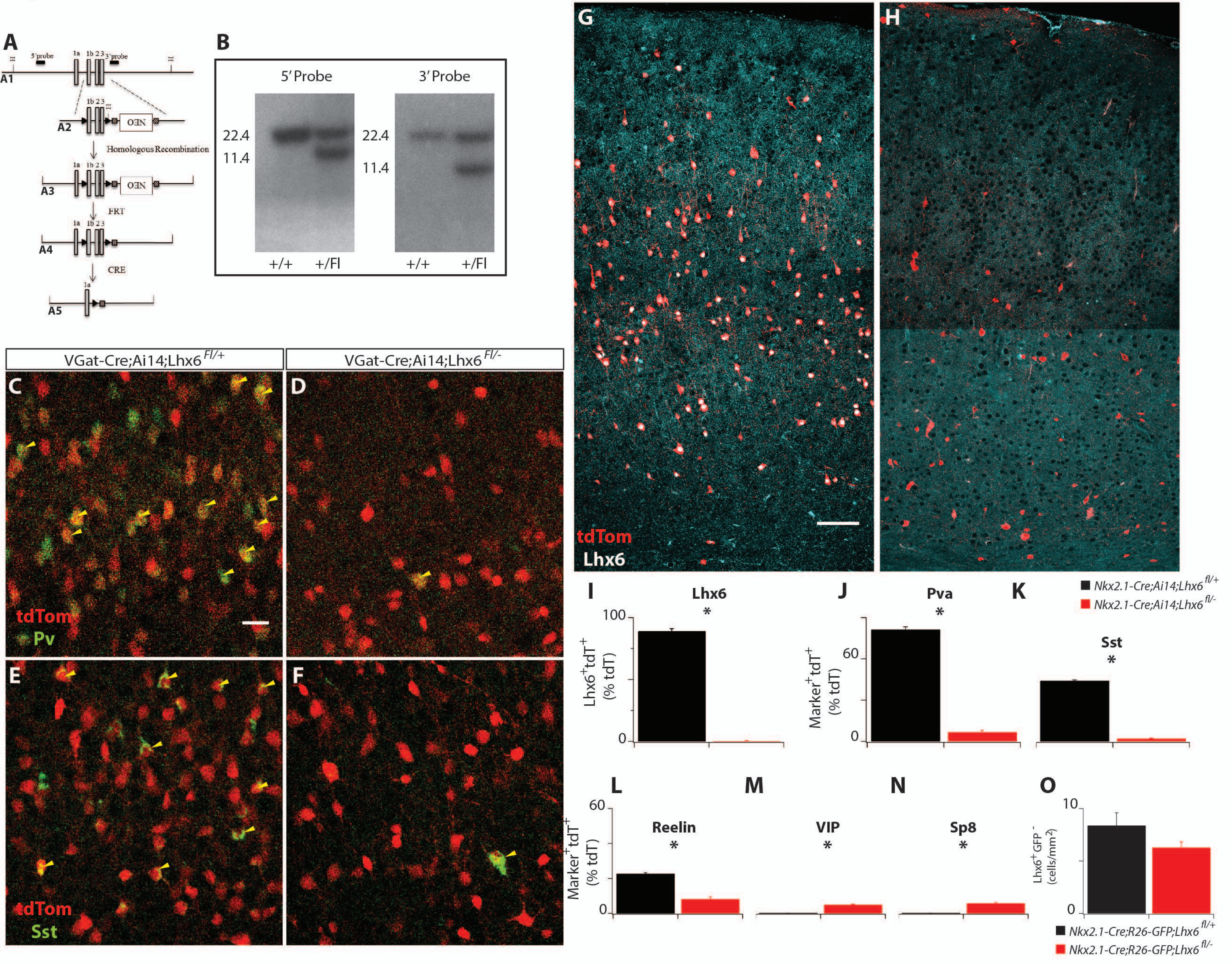

A
5'probe
3'probe
1a
1b
2 3
H
A1
A2
NEO
Homologous Recombination
A3
FRT
A4
CRE
A5
B
5' Probe
3' Probe
22.4
11.4
+/+
+/Fl
VGat-Cre;Ai14;Lhx6 Fl/+
VGat-Cre;Ai14;Lhx6 Fl/-
C
D
E
F
tdTom
Pv
Sst
Nkx2.1-Cre;Ai14;Lhx6 Fl/+
Nkx2.1-Cre;Ai14;Lhx6 Fl/-
G
H
Lhx6
I
J
K
Pva
Sst
Lhx6+tdT+ (% tdT)
Marker+tdT+ (% tdT)
100
60
0
*
Nkx2.1-Cre;Ai14;Lhx6 fl/+
Nkx2.1-Cre;Ai14;Lhx6 fl/-
L
M
N
O
Reelin
VIP
Sp8
10
Lhx6+GFP- (cells/mm2)
Nkx2.1-Cre;R26-GFP;Lhx6 fl/+
Nkx2.1-Cre;R26-GFP;Lhx6 fl/-

Figure S1: Generation and characterization of a conditional *Lhx6* allele (**A**) Schematic representation of the strategy used to generate the *Lhx6* ^{fl} allele by homologous recombination. A1 shows the targeted region of the wild-type *Lhx6* locus. Exons 1a, 1b (generated by alternative splicing), 2 and 3 as well as the probes used for Southern blot analysis are indicated. H, HindIII. A2 shows the targeting construct including exons 1b, 2 and 3 flanked by loxP sites and PGK-Neo positive selection cassette flanked by FRT sites. The targeted *Lhx6* allele following homologous recombination and removal of the PGK-Neo cassette (by FlpE-induced recombination of the FRT sites) is shown in A3 and A4, respectively. A5, following CRE-mediated recombination exons 1b, 2 and 3 are deleted. (**B**) Southern Blot analysis of HindIII digested genomic DNA from wild type (*Lhx6*$^{+/+}$) and *Lhx6*$^{fl/+}$ animals hybridized with 5' and 3' homology probes verifying the generation of the *Lhx6*^{fl} allele. (**C,F**) Coronal sections from the somatosensory cortex of P18 control *VGat-Cre;Ai14;Lhx6*$^{+/fl}$ (Ctrl; C,E) or mutant *VGat-Cre;Ai14;Lhx6*$^{fl/-}$ (Mut; **D,F**) littermates were immunostained with polyclonal antibodies against Pv (**C,D**) or Sst (**E,F**). Note a large fraction of CIs (tdT^{+}, in red) express Pv and Sst (arrowheads) in control, but not in mutant sections. (**G,H**) Cryosections from the somatosensory cortex of P18 control *Nkx2.1-Cre;Ai14;Lhx6*$^{+/fl}$ (Ctrl; **G**) or mutant *Nkx2.1-Cre;Ai14;Lhx6*$^{fl/-}$ (Mut; **H**) littermates were immunostained with a polyclonal Lhx6 antibody. The *Lhx6*^{fl} allele was recombined with the *Nkx2.1::Cre* transgene and recombination was followed using the Cre-dependent Ai14 (tdT) reporter. Lhx6 expression (blue) is undetectable in MGE CIs (red) in *Lhx6*$^{fl/-}$ mice. (**I**) Quantification of Lhx6^{+} MGE-derived CIs in Ctrl and Mut mice. (**J-N**) Quantification of the co-localization of MGE and CGE markers in Ctrl and Mut mice. Note the dramatic reduction of MGE specific CI markers and the appearance of residual expression of CGE-specific markers. (**O**) Quantification of Lhx6^{+} not expressing GFP (reporter of Cre recombination) in Ctrl and Mut mice. Scale bars: 50 microns (**C-F**), Data expressed as mean ± SEM. Statistical significance evaluated using Student's t-test, * denotes $p < 0.05$.

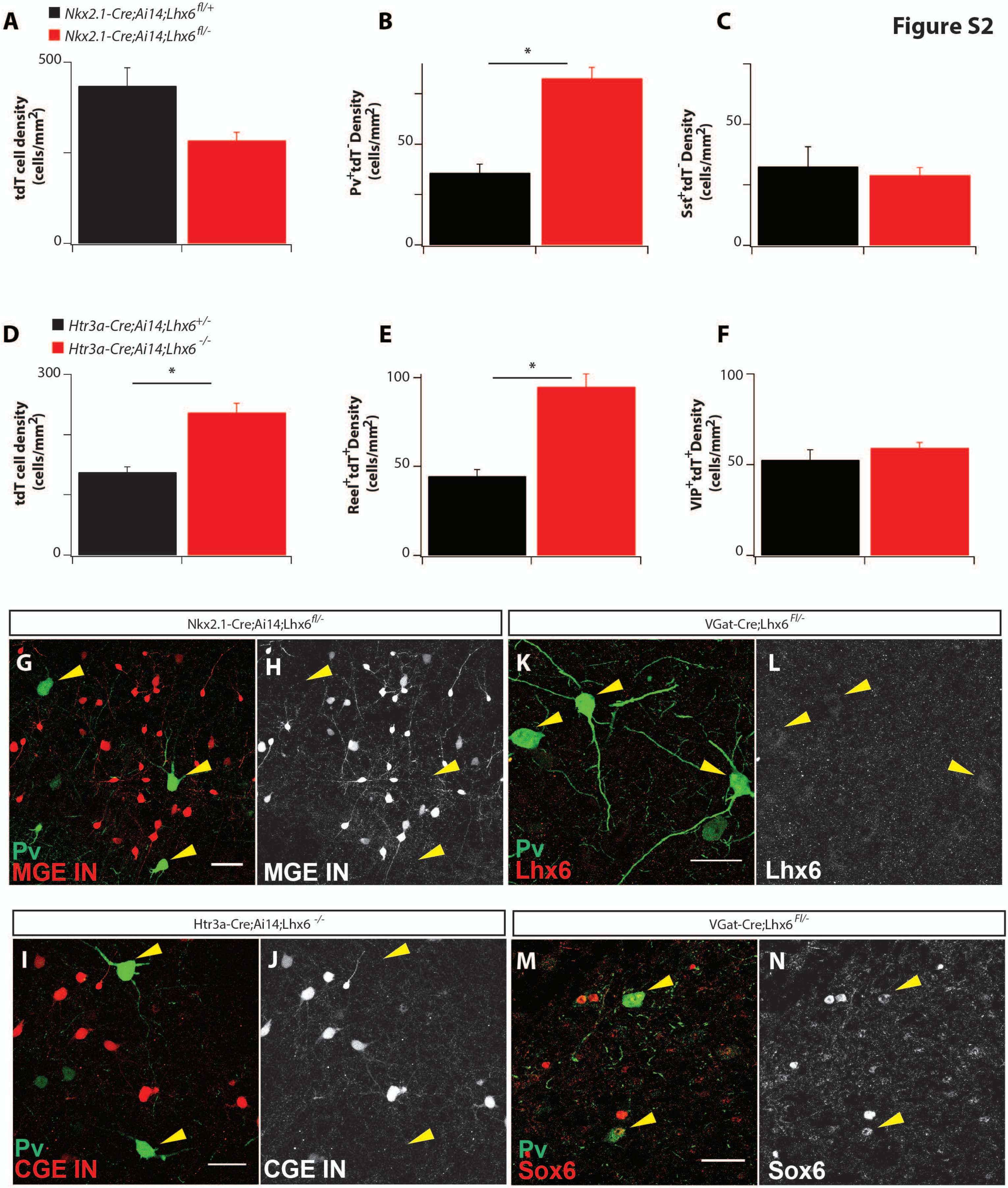
Figure S2
A
Nkx2.1-Cre;Ai14;Lhx6 fl/+
Nkx2.1-Cre;Ai14;Lhx6 fl/-
tdT cell density (cells/mm2)
500
0
B
Pv+tdT- Density (cells/mm2)
50
0
*
C
Sst+tdT- Density (cells/mm2)
50
0
D
Htr3a-Cre;Ai14;Lhx6+/-
Htr3a-Cre;Ai14;Lhx6 -/-
tdT cell density (cells/mm2)
300
0
*
E
Reel+tdT+Density (cells/mm2)
100
50
0
*
F
VIP+tdT+Density (cells/mm2)
100
50
0
Nkx2.1-Cre;Ai14;Lhx6fl/-
G
Pv
MGE IN
H
MGE IN
VGat-Cre;Lhx6 Fl/-
K
Pv
Lhx6
L
Lhx6
Htr3a-Cre;Ai14;Lhx6 -/-
I
Pv
CGE IN
J
CGE IN
VGat-Cre;Lhx6 Fl/-
M
Pv
Sox6
N
Sox6

Figure S2: Characterisation of CIs in mature (P18) Lhx6 control and mutant brains, related to Figure 1. Quantification of CI subtypes in the motor cortex of *Lhx6*$^{fl/+}$ (Ctrl) and *Lhx6*$^{fl/-}$ (Mut) mice. The data in panels **A-F** are from the same animals used for Figure 1. Specifically, **A, B, C, D, E** and **F** correspond respectively to panels **H, O, P, I, M** and **N** of Figure 1. Note that the changes observed in motor cortex are similar to those observed in the somatosensory cortex. Pv expressing cells in *Lhx6* null mutants do not derive from the MGE (**G-H**) or the CGE (**I**-**J**). (**G-J**) Coronal sections from the cortex of P18 *Nkx2.1-Cre;Ai14;Lhx6*$^{fl/-}$ (**G**) and *Htr3a-Cre;Ai14;Lhx6*$^{-/-}$ (**I**) mice immunostained for Pv (green). CIs derived from the MGE (**G**) or CGE (**I**) are tdT^{+} (red). In panels **H** and **J**, which correspond to **G** and **I** respectively, the lineage tracer is shown in white. It has been shown that a fraction of MGE derived CIs do not express Cre in the Nkx2.1-Cre line used in this study (Kessaris et al., 2006). However, Pv expressing in *Lhx6* mutants do not express Lhx6 (E-F), but express Sox6 at low levels, suggesting they originate in the POA (Gelman et al., 2011). (**K-N**) Coronal sections from P18 *Lhx6*$^{-/-}$ mice double immunostained for Pv (green) and Lhx6 (red in **K**) and Sox6 (red in **M**). In panels **L** and **N**, which correspond to **K** and **M** respectively, the Lhx6 and Sox6 signal is shown in white. Yellow arrowheads indicate Pv-expressing cells. Scale bars: 50 microns.

Figure S3

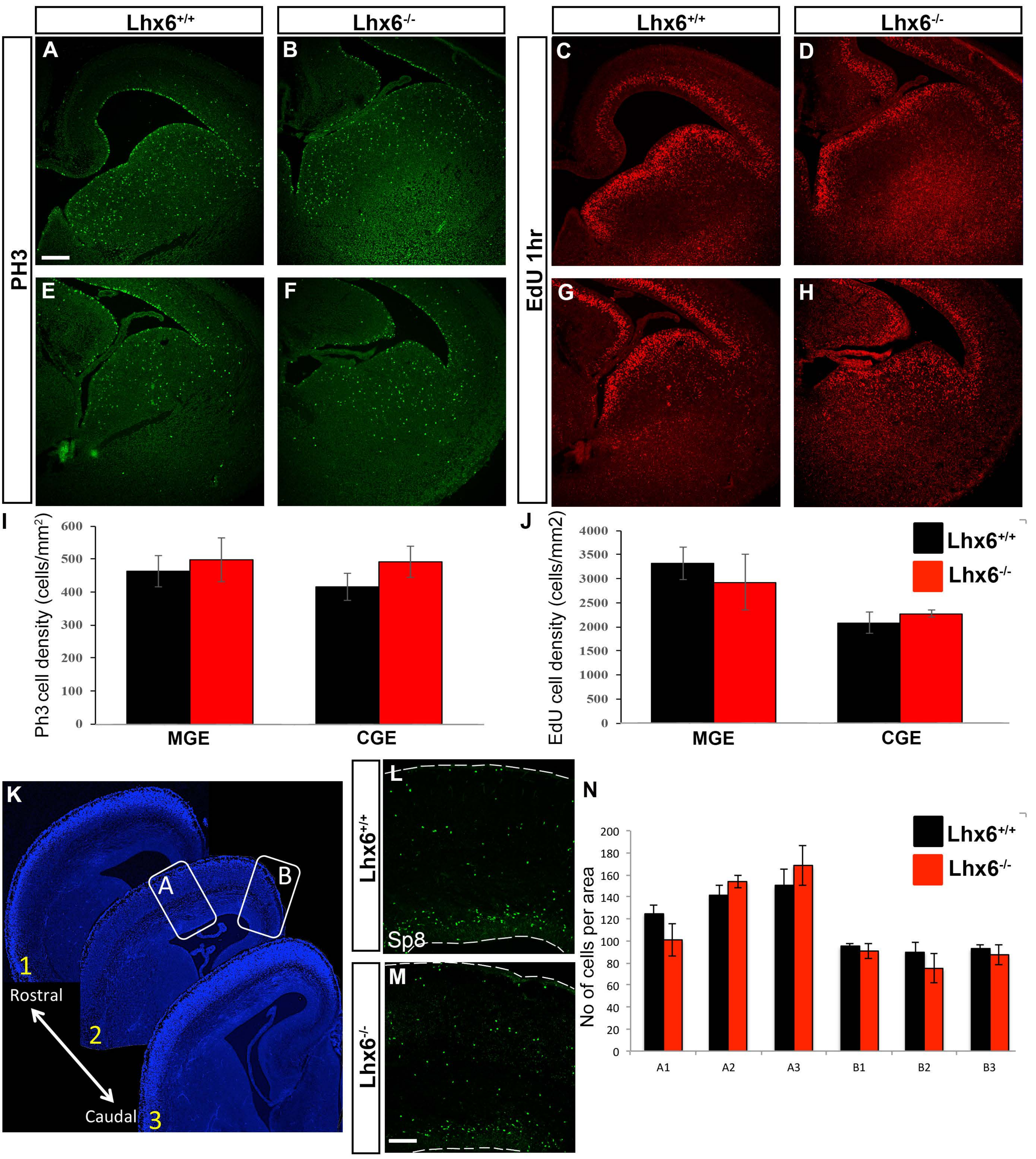

Lhx6+/+
Lhx6-/-
Lhx6+/+
Lhx6-/-
PH3
EdU 1hr
A
B
C
D
E
F
G
H
I
J
Ph3 cell density (cells/mm2)
600
500
400
300
200
100
0
MGE
CGE
EdU cell density (cells/mm2)
4000
3500
3000
2500
2000
1500
1000
500
0
Lhx6+/+
Lhx6-/-
K
A
B
1
2
3
Rostral
Caudal
L
M
Sp8
Lhx6+/+
Lhx6-/-
N
No of cells per area
200
180
160
140
120
100
80
60
40
20
0
A1
A2
A3
B1
B2
B3
Lhx6+/+
Lhx6-/-

Figure S3: No changes in proliferation or migration of CI precursors in *Lhx6* mutant embryos, related to Figure 2. (**A,B,E,F**) Coronal brain sections from E14.5 wild-type (**A,E**) and *Lhx6* mutant (**B**,**F**) mouse embryos immunostained for pH3. Sections shown in **A** and **B** are from the MGE level while sections shown in **E** and **F** are from the CGE level. (**C,D,G,H**) The set of sections shown in **A,B,E** and **F** imaged for EdU. (**I-J**) Quantification of results shown in (**A-H**). (**K**) DAPI staining of 3 different sections across the rostro-caudal axis of an E16.5 wild-type mouse brain, representing the areas analysed for Sp8 immunostaining. Boxes indicate the two areas (A and B) along the latero-medial axis used for $Sp8^+$ CI quantification shown in **N**. (**L, M**) $Sp8^+$ migrating interneurons (green) in cortical sections of the A2 brain area (**K**) from wild-type (**L**) and *Lhx6* mutant (**M**) E16.5 embryos. (**N**) Quantification of $Sp8^+$ CIs along different regions of $Lhx6^{+/+}$ (black) and $Lhx6^{-/-}$ (red) E16.5 mouse brains. Scale bars: 200 microns (A-H), 100 microns (L-M).

Figure S4

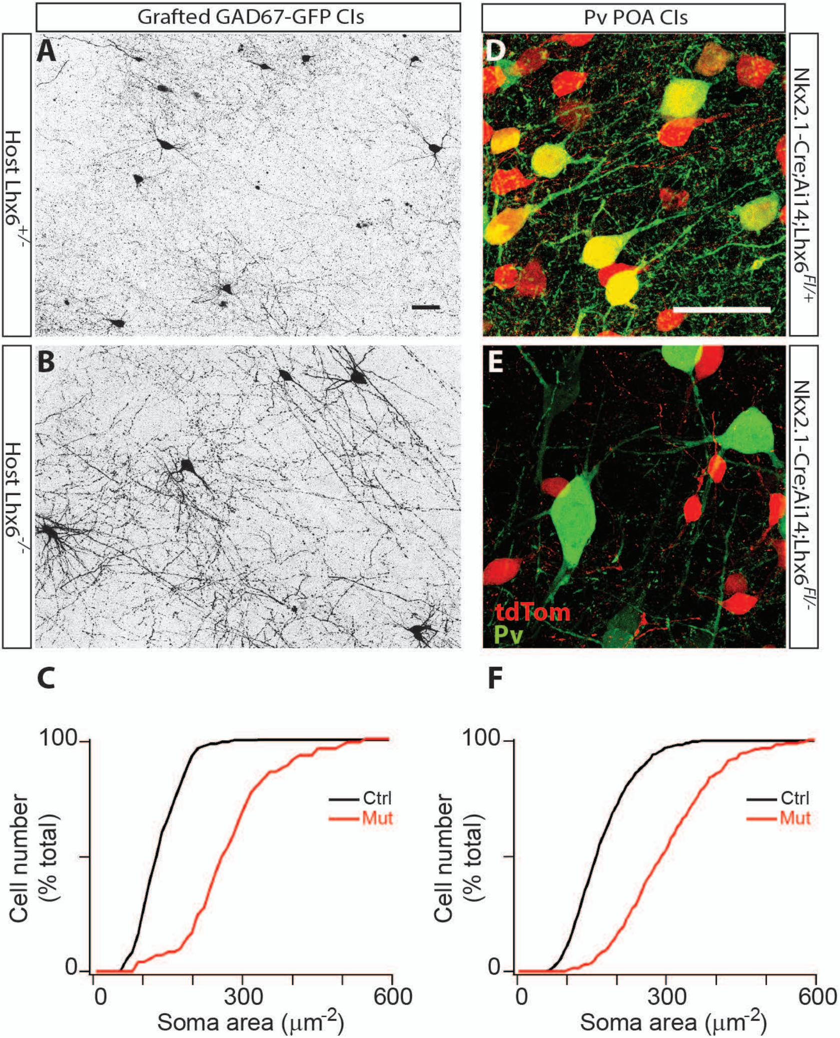

Grafted GAD67-GFP CIs
Pv POA CIs
A
B
C
D
E
F
Host Lhx6+/-
Host Lhx6-/-
Nkx2.1-Cre;Ai14;Lhx6Fl/+
Nkx2.1-Cre;Ai14;Lhx6Fl/-
tdTom
Pv
100
0
0
300
600
Cell number (% total)
Soma area (μm-2)
Ctrl
Mut

Figure S4: Large soma area observed in both grafted CIs and POA derived CIs in *Lhx6* mutant brains, Related to Figure 3. (**A-B**) Coronal sections from the somatosensory cortex of P16 wild type (**A**) and *Lhx6* mutant (**B**) mice grafted with wild type GAD67:GFP CI progenitors. Grafted CI morphology is revealed by GFP expression (converted to greyscale). (**D-E**) Coronal sections from the somatosensory cortex of P18 *Nkx2.1-Cre;Ai14;Lhx6*$^{fl/+-}$ (**D**) and *Nkx2.1-Cre;Ai14;Lhx6*$^{fl/-}$ (**E**) immunostained for Pv (green). MGE derived CIs can be identified by tdT expression (red). Scale bars: 50 microns. (**C,F**) Quantification of soma size. Cumulative histograms shown in **C** and **F** correspond to the animals shown in **A/B** and **D/E**, respectively. Black: Ctrl; Red: Mut.

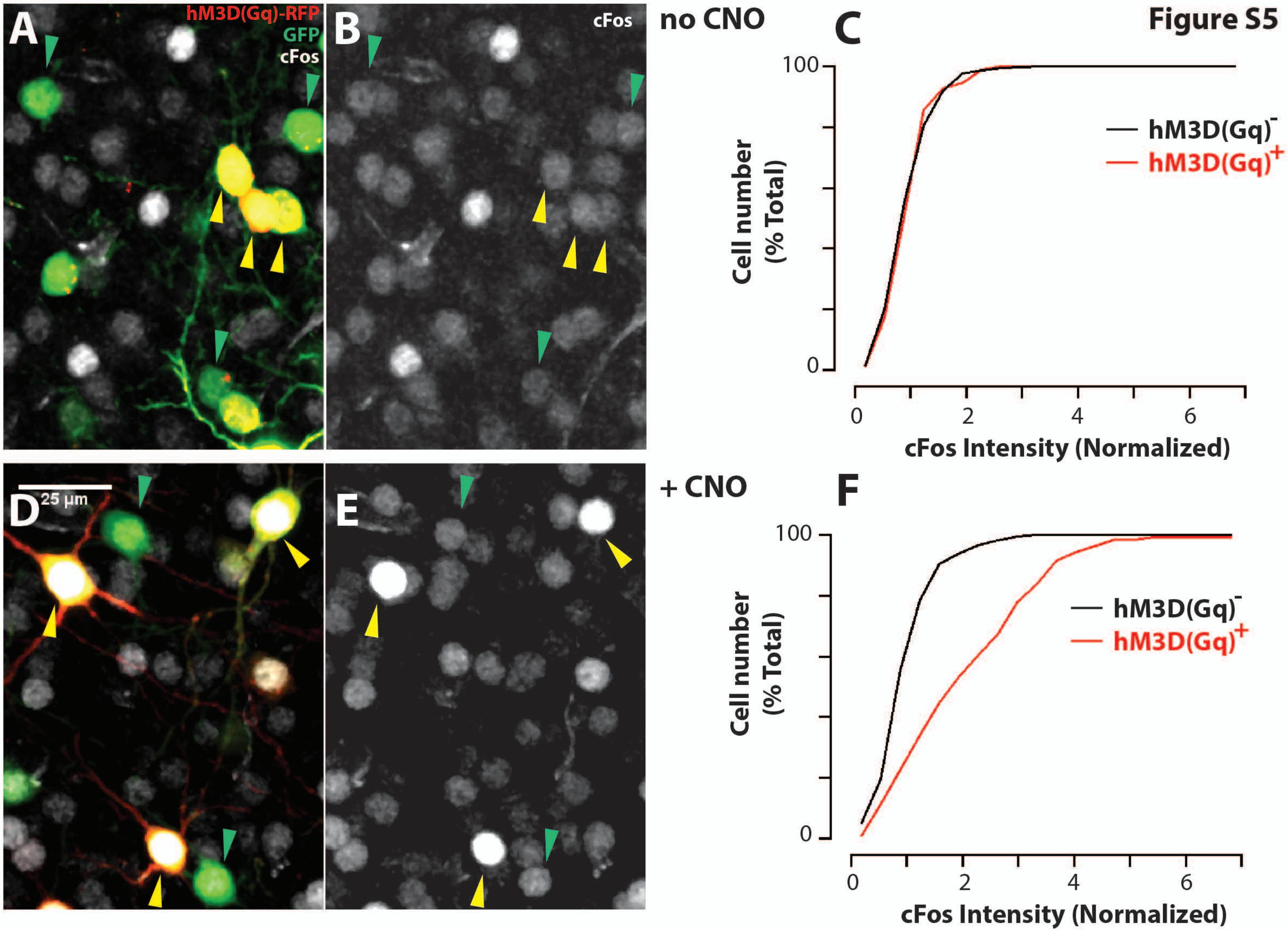

Figure S5
A
hM3D(Gq)-RFP
GFP
cFos
B
cFos
no CNO
C
100
0
hM3D(Gq)-
hM3D(Gq)+
Cell number (% Total)
0
2
4
6
cFos Intensity (Normalized)
D
25 µm
E
+ CNO
F
100
0
hM3D(Gq)-
hM3D(Gq)+
Cell number (% Total)
0
2
4
6
cFos Intensity (Normalized)

Figure S5: **Nuclear c-fos expression increases in hM3D(Gq) expressing CIs following CNO administration, Related to Figure 5**. (**A,B,D,E**) Confocal micrographs of coronal sections from the brain of mice transplanted at P0-P2 with CI precursors transfected with CAG:hM3D(Gq):IRES:RFP and CAG:IRES:GFP expression plasmids and immunostained for GFP (green), RFP (red) and c-fos (white). Sections are from vehicle only (**A**) or CNO (**D**) treated animals. Panels B and E are identical to those in A and D respectively, but show only the c-fos signal (white). (**C,F**) Cumulative histogram of normalized c-fos intensity in nuclei from cells that are either negative (black) or positive (red) for RFP (hM3D(Gq)). For CNO treated animals (**F**) the mean c-fos intensity of DREADD-Gq Positive nuclei was 2.17 ± 0.11 (n = 141 cells) while for DREADD-Gq Negative nuclei it was only 1.11 ± 0.04 (n = 209 cells, 3 mice ; significant by Wilcoxon rank test p<10-10). In contrast, for untreated animals (**C**) the mean cfos intensity of DREADD-Gq Positive nuclei was 1.1 ± 0.04 (n = 111 cells) and for DREADD-Gq Negative nuclei was 1.07 ± 0.02 (n = 338 cells, 3 mice ; not significant by Wilcoxon rank test p=0.5). Scale bars: 25 microns.

Table S1

Fate Mapping (Figures 1, 2, S1, S2)

**Cell Type**	**Average Control**	**SEM Control**	**Average Mutant**	**SEM Mutant**	**p**	**Test Used**
MGE CI SSC (cells/mm^2)	471	34	308	23	**0.02**	Student's t test
CGE CI SSC (cells/mm^2)	165	12	305	35	**0.01**	Student's t test
pan CI SSC (cells/mm^2)	650	31	631	9	0.6	Student's t test
MGE CI Layer I (% total)	5.3	0.6	20.4	1.3	**0.002**	Student's t test
MGE CI Layer II/III (% total)	18.3	3.5	10.1	1.9	0.12	Student's t test
MGE CI Layer IV (% total)	33	0.9	21	1.2	**0.001**	Student's t test
MGE CI Layer V (% total)	35.2	1.8	22.4	1.2	**0.006**	Student's t test
MGE CI Layer VI (% total)	13.9	2	28	0.9	**0.01**	Student's t test
CGE CI Layer I (% total)	14.4	1.8	13.4	2.1	0.7	Student's t test
CGE CI Layer II/III (% total)	60.2	3.6	35.5	2.9	**0.007**	Student's t test
CGE CI Layer IV (% total)	11.9	0.4	11.8	0.3	0.7	Student's t test
CGE CI Layer V (% total)	4.8	1	9.7	1.2	**0.03**	Student's t test
CGE CI Layer VI (% total)	8.7	2.8	29.7	1.3	**0.007**	Student's t test
pan CI Layer I (% total)	7.2	1.1	14.2	0.9	**0.01**	Student's t test
pan CI Layer II/III (% total)	48.3	1.3	24.6	1.8	**0.0006**	Student's t test
pan CI Layer IV (% total)	17.9	1.5	12.6	0.8	0.05	Student's t test
pan CI Layer V (% total)	16.1	1	18.1	1.8	0.4	Student's t test
pan CI Layer VI (% total)	10.5	0.8	30.4	2.2	**0.006**	Student's t test
Reelin$^+$ CGE$^+$ SSC (cells/mm^2)	49.9	5.7	84.3	7	**0.02**	Student's t test
VIP$^+$ CGE$^+$ SSC (cells/mm^2)	43	5	41.2	2.7	0.7	Student's t test

Pv^+ MGE^- SSC (cells/mm^2)	29.7	0.4	69.2	3.8	**0.009**	Student's t test
Sst^+ MGE^- SSC (cells/mm^2)	28	2.5	24.3	2.6	0.4	Student's t test
P2 MGE CI (cells/mm^2)	742	3	745	29	0.9	Student's t test
P2 CGE CI (cells/mm^2)	200	4.8	177.3	14	0.2	Student's t test
P7 MGE CI (cells/mm^2)	390	21	278	15	**0.01**	Student's t test
P7 CGE CI (cells/mm^2)	113	10	174	3.8	**0.02**	Student's t test
P2 $TUNEL^+MGE^+$ (% MGE^+)	0.22	0.04	0.93	0.09	0.07	Student's t test
P2 $TUNEL^+Sp8^+$ (% $Sp8^+$)	0.15	0.08	0.26	0.02	0.07	Student's t test
P7 $TUNEL^+MGE^+$ (% MGE^+)	3.1	0.4	7.6	1.3	**0.01**	Student's t test
P7 $TUNEL^+CGE^+$ (% CGE^+)	2	0.1	0.7	0.1	**0.0006**	Student's t test
MGE CI MC (cells/mm^2)	436	49	285	22	0.07	Student's t test
CGE CI MC (cells/mm^2)	138	9	237	15	**0.002**	Student's t test
$Reelin^+$ CGE^+ MC (cells/mm^2)	44.7	3.5	94.9	7.1	**0.009**	Student's t test
VIP^+ CGE^+ MC (cells/mm^2)	52.8	5.3	59.4	2.8	0.5	Student's t test
Pv^+ MGE^- MC (cells/mm^2)	36	4.1	82.8	5.3	**0.003**	Student's t test
Sst^+ MGE^- MC (cells/mm^2)	32.6	8.1	29.1	3	0.7	Student's t test
$Lhx6^+MGE^+$ (% MGE^+)	89.3	4.9	0.5	0.3	**0.003**	Student's t test
Pv^+MGE^+ (% MGE^+)	54.2	1.7	4.7	0.5	**0.001**	Student's t test
Sst^+MGE^+ (% MGE^+)	29	1.1	0.3	0.2	**0.001**	Student's t test
$Reelin^+MGE^+$ (% MGE^+)	22.9	1	8	1.9	**0.005**	Student's t test
VIP^+MGE^+ (% MGE^+)	0	0	5.1	0.4	**0.005**	Student's t test
$Sp8^+MGE^+$ (% MGE^+)	0	0	5.8	1	**0.03**	Student's t test

Grafting GAD67:GFP CIs (Figure 3)

Individual	Average Control Host	SEM Control Host	Mutant Host	N animals Control	n Cells Control	n Cells Mutant
Experiment 1 (% Control Average)	100	13	198	4	4933	2441
Experiment 2 (% Control Average)	100	9	184	5	16927	6223
Experiment 3 (% Control)	100		157	1	5434	8554
**Average**	**Average Mutant Host**	**SEM Mutant Host**	**p**	**Test Used**		
(% Control Average)	181	13	**0.02**	Student's t test single sample (mean 100)		

Grafting hM3D(Gq) expressing CI (Figure 5)

	Average Vehicle	SEM Vehicle	Average CNO	SEM CNO	p	test
RFP^+ (% GFP^+)	47	3	61	3	**0.01**	Student's t test (paired samples)
**Individual Experiments**	**Fraction Vehicle**	**Fraction CNO**	**N Cells Vehicle**	**N Cells CNO**		
Experiment 1 RFP^+ (% GFP^+)	53.63	65.76	330	444		
Experiment 2 RFP^+ (% GFP^+)	45.34	62.34	397	308		
Experiment 3 RFP^+ (% GFP^+)	41.67	54.86	228	175		

Soma Cell Size (Figure S4)

Cell Type	Average Control	SEM Control	Average Mutant	SEM Mutant	p	Test	n Cells Control	n Cells Mutant
Pv^+ MGE^- (μm^2)	171	2	300	7	**$<10^{-10}$**	Wilcoxon rank	701	199
	**Average Control Host**	**SEM Control Host**	**Average Mutant Host**	**SEM Mutant Host**	**p**	**Test**	**n Cells Control Host**	**n Cells Mutant Host**
Grafted CI (μm^2)	138	4	275	11	**$<10^{-10}$**	Wilcoxon rank	119	72

Table S1, Related to all Figures: Summary of statistical analysis.

Table S3:

**Up-regulated genes**	**$2^{(-DDCT)}$**	**Down-regulated genes**	**$2^{(-DDCT)}$**
Adam10	1.003	***Adcy1***	0.904
Adcy8	1.309	***Akt1***	0.809
Arc	1.257	***Camk2a***	0.768
Bdnf	**2.070**	***Camk2g***	0.889
Dlg4	1.053	***Cdh2***	0.750
Egr1	1.158	***Cebpb***	0.500
Egr2	**2.942**	***Cnr1***	0.865
Egr3	**1.983**	***Creb1***	0.766
Fos	1.172	***Crem***	**0.419**
Gabra5	1.447	***Egr4***	0.927
Grin1	1.086	***Ephb2***	0.919
Grin2a	1.104	***Gnai1***	0.783
Grin2d	1.024	***Gria1***	0.666
Grm4	1.341	***Gria2***	0.733
Igf1	**2.334**	***Gria3***	0.730
Kif17	1.437	***Gria4***	0.800
Mmp9	**1.522**	***Grin2b***	0.806
Nfkb1	1.243	***Grin2c***	0.556
Ngfr	**3.418**	***Grip1***	0.912
Nptx2	1.081	***Grm1***	0.597
Pick1	1.082	***Grm2***	**0.300**
Prkca	1.140	***Grm3***	0.546
Prkcg	1.189	***Grm5***	0.832
Prkg1	**5.387**	***Grm7***	0.759
Rab3a	1.176	***Grm8***	0.947
Rgs2	1.029	***Homer1***	0.740
Rheb	1.353	***Inhba***	0.701
Synpo	**2.349**	***Jun***	0.634
		Junb	0.845

		Klf10	0.685
		Mapk1	0.837
		Ncam1	0.758
		Ngf	0.694
		Nos1	0.968
		Nr4a1	0.626
		Ntf3	**0.221**
		Ntrk2	0.699
		Pcdh8	0.969
		Pim1	**0.469**
		Plat	0.781
		Pclg1	0.749
		Ppp1ca	0.916
		Ppp1cc	0.792
		Ppp2ca	0.910
		Ppp3ca	0.796
		Rela	0.827
		Reln	0.807
		Sirt1	**0.461**
		Srf	0.872
		Timp1	0.519
		Ywhaq	0.949

Table S3: Expression changes of activity associated genes in *Htr3aCre;Ai14;Lhx6*$^{-/-}$ compared to *Htr3aCre;Ai14;Lhx6*$^{+/-}$ mice at P7, Related to Figure 4. List of genes analysed (see also Supplemental Experimental Procedures). Red highlighted genes: transcript up-regulation >1.5, green highlighted genes: transcript down-regulation <0.5, orange highlighted genes: up-regulation of transcripts has been previously observed by RNA *in situ* hybridization (Arc, Egr1; Figures 4 E-H), while in the case of *cfos* increased protein levels have been observed by immunohistochemistry (Figures 4 I-Q).
